# The influence of single nucleotide polymorphisms of *NOD2* or *CD14* on the risk of *Mycobacterium tuberculosis* diseases: a systematic review

**DOI:** 10.1186/s13643-021-01729-y

**Published:** 2021-06-09

**Authors:** Juan M. Cubillos-Angulo, Catarina D. Fernandes, Davi N. Araújo, Cristinna A. Carmo, María B. Arriaga, Bruno B. Andrade

**Affiliations:** 1 Instituto Gonçalo Moniz, Fundação Oswaldo Cruz, Salvador, Bahia Brazil; 2 Faculdade de Medicina, Universidade Federal da Bahia, Salvador, Bahia Brazil; 3 Multinational Organization Network Sponsoring Translational and Epidemiological Research (MONSTER) Initiative, Salvador, Bahia Brazil; 4 Curso de Medicina, Universidade Salvador (UNIFACS), Laureate Universities, Salvador, Bahia Brazil; 5 Curso de Medicina, Faculdade de Tecnologia e Ciências (FTC), Salvador, Bahia Brazil; 6 Curso de Medicina, Escola Bahiana de Medicina e Saúde Pública, Salvador, Bahia Brazil; 7 Division of Infectious Diseases, Department of Medicine, Vanderbilt University School of Medicine, Nashville, TN USA; 8 Wellcome Centre for Infectious Disease Research in Africa, Institute of Infectious Disease and Molecular Medicine, University of Cape Town, Cape Town, South Africa

**Keywords:** NOD2, CD14, Single nucleotide polymorphism, Tuberculosis

## Abstract

**Background:**

Tuberculosis (TB) is still one of the leading causes of death worldwide. Genetic studies have pointed to the relevance of the *NOD2* and *CD14* polymorphic alleles in association with the risk of diseases caused by *Mycobacterium tuberculosis* (*Mtb*) infection.

**Methods:**

A systematic review was performed on PubMed, EMBASE, Scientific Electronic Library Online (SciELO), and Literatura Latino-Americana e do Caribe em Ciências da Saúde (Lilacs) to examine the association between single nucleotide polymorphisms (SNP) and risk of *Mtb* diseases. Study quality was evaluated using the Newcastle-Ottawa Quality Scale (NOQS), and the linkage disequilibrium was calculated for all SNPs using a webtool (Package LDpop).

**Results:**

Thirteen studies matched the selection criteria. Of those, 9 investigated *CD14* SNPs, and 6 reported a significant association between the T allele and TT genotypes of the rs2569190 SNP and increased risk of *Mtb* diseases. The genotype CC was found to be protective against TB disease. Furthermore, in two studies, the *CD14* rs2569191 SNP with the G allele was significantly associated with increased risk of *Mtb diseases*. Four studies reported data uncovering the relationship between *NOD2* SNPs and risk of *Mtb* diseases, with two reporting significant associations of rs1861759 and rs7194886 and higher risk *of Mtb* diseases in a Chinese Han population. Paradoxically, minor allele carriers (CG or GG) of rs2066842 and rs2066844 *NOD2* SNPs were associated with lower risk of *Mtb* diseases in African Americans.

**Conclusions:**

The *CD14* rs2569190 and rs2569191 polymorphisms may influence risk *of Mtb* diseases depending on the allele. Furthermore, there is significant association between *NOD2* SNPs rs1861759 and rs7194886 and augmented risk *of Mtb* diseases, especially in persons of Chinese ethnicity. The referred polymorphisms of *CD14* and *NOD2* genes likely play an important role in risk *of Mtb* diseases and pathology and may be affected by ethnicity.

**Systematic review registration:**

CRD42020186523

**Supplementary Information:**

The online version contains supplementary material available at 10.1186/s13643-021-01729-y.

## Background

Tuberculosis (TB) is one of the 10 leading causes of death around the world [1]. Approximately 1.7 billion people are infected by *Mycobacterium tuberculosis* (*Mtb*) worldwide [1]. The occurrence of this infection at different rates across countries and ethnicities indicates that genetic determinants may underlay the risk of developing diseases caused by *Mtb* infection such as pulmonary or extrapulmonary TB (referred hereafter as *Mtb* diseases) [2]. Work to date has highlighted notable gaps in factors that influence the risk *of Mtb* diseases [3]. For example, the associations of host genetic factors with *Mtb* infection have not been validated in multiple populations, and some study findings are inconsistent [3].

The immune system has a fundamental role in response to *Mtb* [4]. Thus, it is expected that polymorphisms in immune-related genes may directly affect the capacity of a host exposed to Mtb to control infection. Indeed, many studies have reported relationships between SNPs of immune-related genes and risk *of Mtb* diseases, such as the association between SNPs in *TLR4* [5], *TNFA* [6], and increased risk of active TB among highly exposed individuals. In addition to these genes, the nucleotide-binding oligomerization Domain-Containing protein 2 (*NOD2*) and Cluster Differentiation antigen 14 (*CD14*) genes are frequently studied in this setting, as these genes account for proteins that act in the recognition of mycobacterial molecular patterns and lead to immune activation against Mtb [7, 8]. While prior studies reported on the role of *NOD2* and *CD14*, many have disparate results, and often are restricted to certain populations [9].

The *CD14* gene codifies a glycosylphosphatidylinositol-anchored surface molecule present on the surface of monocytes, macrophages, and polymorphonuclear leucocytes, which functions as a key pattern recognition receptor (PRR) protein in innate immunity. CD14 plays a role in mediating signals from Toll-like receptors (TLRs) that recognize Mtb [10]. Additionally, *CD14* is critical to mounting an adequate innate response to aerogenic infection with Mtb [11]. Several studies have investigated whether risk *of Mtb* diseases is influenced by polymorphisms of this gene, though results have been inconsistent and inconclusive [4, 12]. For that reason, it remains difficult to determine the role of CD14 on risk *of Mtb* diseases in different populations, as studies with distinct ethnicities have conflicting results.

*NOD2* is expressed in numerous cell types of the immune system, including macrophages, neutrophils, and eosinophils [13, 14]. It encodes a specialized protein that functions as an intracellular PRR of peptidoglycan through the recognition of muramyl dipeptide (MDP), a motif common to all bacteria [15], with a stimulating signal towards activation of immune responses [16]. When *NOD2* is activated by specific substances produced by bacteria, it turns on a protein complex named nuclear factor kappa-B (NFkB), resulting in transcription of pro-inflammatory mediators [17]. As such, there is mounting evidence that deregulation of *NOD2* signaling causes or contributes to a variety of human diseases, including asthma [18], cancer [19], inflammatory bowel disease [20], and TB [21]. Of note, studies have reported conflicting results on the relationship between *NOD2* SNPs and TB infection, finding mutations in the *NOD2* gene that may lead to both the increased and decreased risk *of Mtb* diseases [22]. Notwithstanding, like studies of *CD14* SNPs, most results diverge depending on the investigated population, leaving several knowledge gaps for a complete understanding of these relationships.

The present study aimed to evaluate work published to date on the influence of polymorphisms of the abovementioned PRRs on risk *of Mtb* diseases. We performed a systematic review to evaluate the association between all reported polymorphisms of *CD14* and *NOD2* and occurrence of *Mtb* diseases, and how such association may differ in distinct ethnic populations.

## Methods

### Study aim

We performed a systematic review on the influence of *CD14* and *NOD2* SNPs on the risk *of Mtb* diseases following the Preferred Reporting Items for Systematic Reviews and Meta-Analyses (PRISMA) recommendations.

### Literature search

A systematic search was conducted between June 01, 2019, and June 25, 2020, by two independent researchers (the authors JMC-A and DNA) in the following databases: PubMed, EMBASE, Scientific Electronic Library Online (SciELO), and Literatura Latino-Americana e do Caribe em Ciências da Saúde (Lilacs). The keywords used in the search were ‘*Mycobacterium tuberculosis*’, ‘tuberculosis’; ‘*CD14*’ or ‘*NOD2*’; and ‘polymorphism’, ‘SNPs’ or ‘genetic polymorphism’ with various combinations. The exact search strategy per database and the number of hits per database are illustrated in the Additional File 1. Every original research article found in the search that was in English, Spanish, or Portuguese was considered, with no restriction on the publication date. Reviews, letters to the editor, and comments were not included but were sources of additional references that did not appear in the first search.

### Selection of studies

**Fig. 1:**
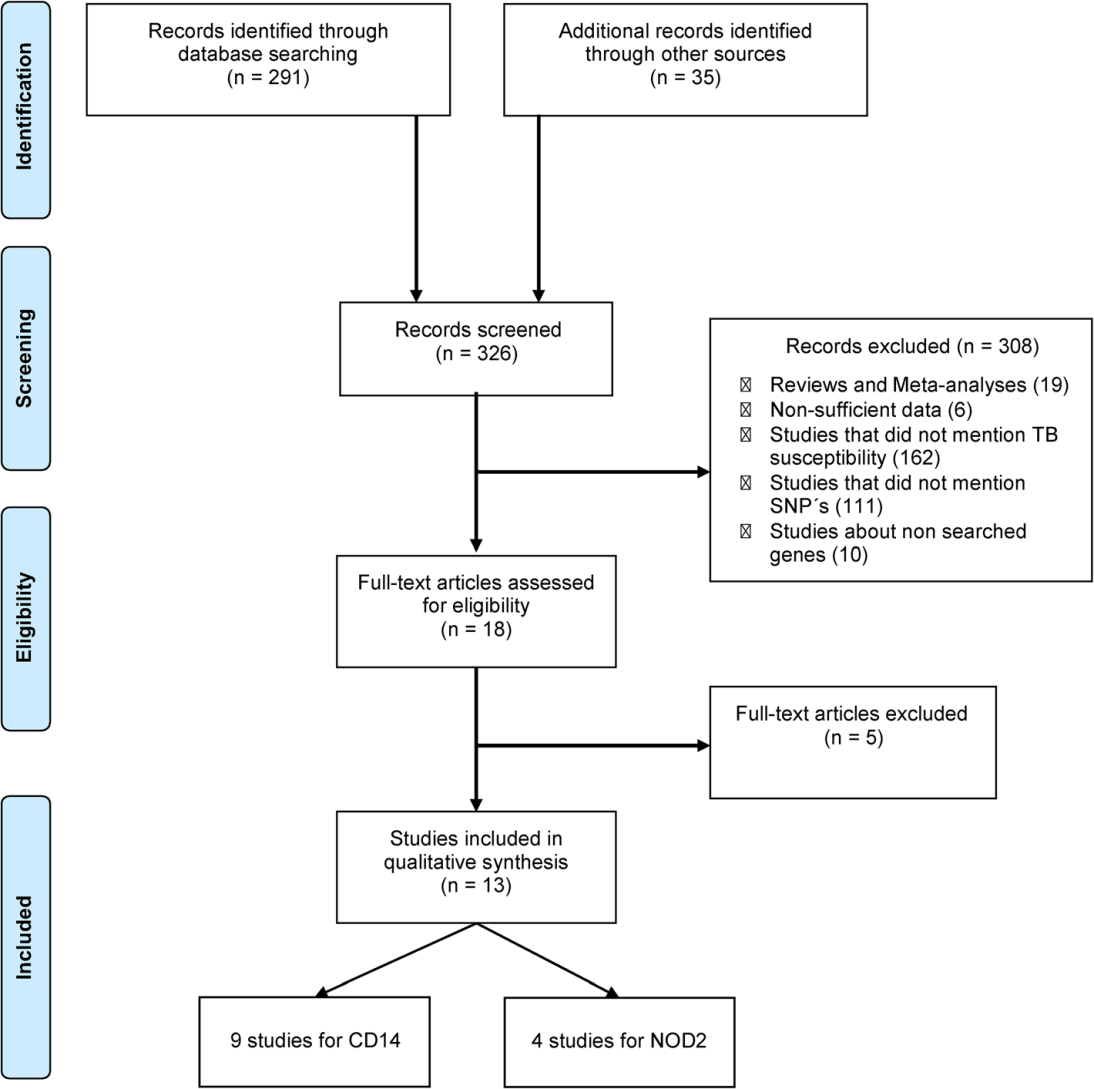
Flowchart of the study selection process study characteristics. The characteristics of the included studies are summarized in Table 1. All the studies (n=13) had a case-control design, with nine publications evaluating *CD14* gene polymorphisms, and four studies analyzing *NOD2* polymorphisms

Initially, titles and abstracts were reviewed and analyzed for eligibility (Fig. 1). Thereafter, all the eligible articles were fully read. These two steps were performed by two independent reviewers (JMC-A and DNA). The inclusion criteria were (1) the main subject of the article must have been the genetic influence on TB and (2) the study must have been related to a SNP in *CD14* and/or *NOD2* genes. Articles that did not mention TB susceptibility or risk *of Mtb* diseases, polymorphism in the genes indicated above, had non-sufficient data, reviews, meta-analyses, animal model studies, letters to the editor, or which were clearly not related to the theme were excluded.

### Data extraction

Data extraction was performed individually by two researchers (DNA and CDF), and discrepancies between them were resolved by a third reviewer (JMC-A). All the information on important variables, publication date, methods, results, and conclusions of the included articles were registered in tables built in Microsoft Excel, made by two different researchers. Lastly, the content of those Excel tables was checked by a third reviewer (JMC-A), attesting the registry compliance.

### Quality assessment

The quality assessment of each individual study was further performed according to the Newcastle-Ottawa Quality Scale (NOQS) [34] (Table 2), which measures the quality of a study based on three aspects: selection (maximum, 4 stars), comparability (maximum, 2 stars), and exposure (maximum, 3 stars). Thus, in the processing of the article quality analysis, a maximum of 9 stars could be obtained. Publication with a total score of 0–3 was classified as low quality, 4–6 as moderate quality, and ≥7 as high quality.

### Linkage disequilibrium

Linkage disequilibrium coefficients were calculated and reported in only three studies [24, 30, 32]. In order to examine the overall profile of the linkage disequilibrium of the SNPs reported in our systematic review, we calculated the linkage disequilibrium for all SNPs of *CD14* and *NOD2* using the Package LDpop [35], establishing an R^2^ cutoff of ≥ 0.8. LDpop takes as input two dbSNP reference SNP numbers and a selection of desired populations from the 1000 Genomes Project which includes sequencing data for 2504 individuals in 26 ancestral populations which are divided into 5 “super populations” [35]. In this approach, we used for the linkage disequilibrium the R^2^ values for all individuals that had reported information for the SNPs.

## Results

### Selection of articles

Our primary search identified a total of 326 articles (Fig. 1). Through the study selection process, 13 articles met the inclusion criteria and were included in the systematic review [4, 12, 23–33]. The majority of the studies evaluated *CD14* gene polymorphisms (n= 9), whereas four studies analyzed *NOD2* polymorphisms. All of the selected studies adopted the case-control design, in which the case was defined as patients with tuberculosis, whether pulmonary, extrapulmonary, or both, and controls were defined as individuals not infected with Mtb. The majority of articles investigated the relationship between presence of polymorphisms and the risk *of Mtb* diseases (n= 7), whereas five studies tested association of SNPs with pulmonary and other forms of TB, and one assessed the relationship with spinal TB (Table 1).

**Table 1:** Characteristics of included studies

Author/year	Country	Study design	Ethnicity	Gene/studied SNP	TB diagnosis	Sample size	Type of study (Susceptibility/resistance to ***Mtb*** diseases or risk of ***Mtb*** diseases)	Type of control	TB screening controls (Tuberculin skin test (TST) or Interferon -gamma Release Assay (IGRA) )	Inclusion and exclusion criteria cases	Inclusion and exclusion criteria controls	Outcomes
Zheng et al., 2018 [23]	China	Case control	Chinese Han	**CD14** rs2569190 C>T	Spinal	240 TB and 150 controls; N= 390	Risk of *Mtb* diseases	Healthy controls	Not reported	Patients diagnosed with extra-pulmonary TB, who tested negative for PTB. Every included patient is free of comorbidities. The spinal TB patients with comorbid disorders or other complications, such as rheumatoid arthritis, congenital cervical anomalies, trauma, prior spinal cervical surgery, HIV-positive, or ankylosing spondylitis were excluded from the present study.	Age- and sex-matched healthy subjects were enrolled as controls.	The frequency of the rs2569190 T allele was significantly higher in spinal TB patients than in controls (OR=1.97, 95% CI= 1.24–3.42) (p<0.01), and the frequency of the CT+TT genotypes (OR=2.10, 95% CI= 1.09–3.85) was also significantly higher in spinal TB patients than in controls (p<0.05).
Xue et al., 2012 [24]	China	Case control	Chinese Han	**CD14** rs2915863 G>A; rs3138078 T>G; rs2569190 C>T; rs2569191 A>G; rs3138076 T>C; rs5744454 T>G, rs5744455 G>T	Pulmonary	318 TB and 380 controls; N= 698	Risk of *Mtb* diseases	The control group was unrelated blood donors with no history of TB or other immune diseases.	Not reported	Patients with PTB confirmed by clinical, radiological, and bacteriological investigation. Patients were excluded if they tested positive for HIV or if they were undergoing immunosuppressive agents.	Healthy individuals, with no history of TB or immune diseases.	The G allele of rs2915863 (OR=1.41, 95% CI=1.12–1.76), G allele of rs3138078 (OR=1.77, 95% CI=1.39–2.24), G allele of rs2569191 (OR=1.78, 95% CI=1.43–2.22), and T allele of rs2569190 (OR=1.73, 95% CI=1.40–2.15, respectively) were significantly associated with TB. rs3138076, rs5744454, and rs5744455, there were no statistically relevant associations
Zhao et al., 2012 [25]	China	Case control	Chinese Han	**CD14** rs2569190 C>T; rs2569191 G>A	Pulmonary and extrapulmonary TB	432 TB and 404 controls; N= 836	Risk of *Mtb* diseases	The control group comprised unrelated blood donors with no history of TB or other immune diseases.	Not reported	Patients were undergoing standard TB treatment at the TB clinic of the Sixth Hospital of Shaoxing and Hangzhou Red Cross Hospital between October 2005 and October 2009. They were excluded if HIV+ or were taking immunosuppressive agents.	Healthy, unrelated blood donors with no history of TB or other immune diseases. All control subjects were from the same ethnic population and geographical origin and were living in the same region as the patients with TB.	Both the frequency of allele T in the rs2569190 (OR= 1.4, 95% CI = 1.148–1.708) and allele G in the rs2569191 (OR = 1.512, 95% CI = 1.236–1.849) were significantly more frequent in cases than in controls and were also significantly associated with TB. The frequencies of genotypes CT and CC in the rs2569190 (OR = 0.46 and 0.63, respectively; 95% CI = 0.34–0.63 and 0.42–0.93), as well as the frequencies of genotypes AG and AA in the rs2569191 (OR = 0.60 and 0.44, respectively; 95% CI = 0.44–0.83 and 0.29–0.65) were lower in cases than in controls and were also protective against the disease.
Alavi-Naini et al., 2012 [26]	Iran	Case control	Persian, Balouch, and Afghan Iranians	**CD14** rs2569190 C>T	Pulmonary	120 TB and 131 controls; N= 251	Risk of *Mtb* diseases	The control group was healthy subjects with absence of clinical symptoms and signs suggestive of active pulmonary TB and normal chest X-ray.	Not reported	Culture-positive PTB patients were included. They had no other comorbidities such as myocardial infarction, septic shock, liver cirrhosis, or pancreatitis.	Healthy subjects matched for age, sex, and ethnicity. The inclusion criteria were absence of clinical symptoms or signs for active TB and normal chest X-ray, no medical history of TB or other infectious or autoimmune diseases.	The frequency of the rs2569190 T allele was 57% in TB patients and 44% in controls and was significantly different (p < 0.002). The risk of *Mtb* diseases was 2.3-fold greater in individuals with the T-allele (CT + TT) than in those without (OR= 2.3, 95% CI= 1.2–4.3, p = 0.006).
Rosas-Taraco et al., 2007 [4]	Mexico	Case control	White and Mestizo Mexican	**CD14** rs2569190 C>T	Pulmonary	111 TB and 174 controls; N = 285	Risk of *Mtb* diseases	Healthy individuals as control subjects	Not reported	All patients had active pulmonary TB diagnosed on the basis of clinical findings and smear or culture positive for PTB. Also, 67 were household contacts who were or were not genetically related to the patients. All participants were negative for HIV and diabetes and not treated with steroids or immunosuppressive agents.	114 healthy individuals. All of them were Mexican older than 18 years.	The frequency of the rs2569190 homozygous TT genotype was highest in patients with pulmonary TB (OR= 3.37, 95% CI= 1.58–7.19 p=<0.002). The frequency of the rs2569190 allele T had a significantly higher risk for the development of pulmonary TB (OR= 2.267; 95% CI= 1.5–3.3).
Ayaslioglu et al., 2012 [27]	Turkey	Case control	Turkish	**CD14** rs2569190 C>T	Pulmonary and extrapulmonary TB	88 TB and 116 controls; N = 204	Risk of *Mtb* diseases	Control group was selected from the adult population who had no underlying comorbidity and no diagnosis of tuberculosis.	Tuberculin skin test (TST)	Subjects who had a diagnosis of tuberculosis; age ≥16 years; and consented to be included into the study. Patients who had infectious diseases in the last 6 weeks, had significant chronic immunosuppressive systemic diseases, was pregnant, or HIV+ were excluded.	Subjects with no known diseases. Patients who had infectious diseases in the last 6 weeks, had significant chronic immunosuppressive systemic diseases, was pregnant, or HIV+ were excluded.	There was no significant difference in terms of genotype distribution between patients with tuberculosis and controls.
Kang et al., 2009 [28]	South Korea	Case control	Korean	**CD14** rs2569190 C>T	Pulmonary and extrapulmonary TB	274 TB and 422 controls; N = 696	Risk of *Mtb* diseases	A control group consisting of 422 healthy blood donors with normal chest X-ray and without respiratory symptoms and signs	Tuberculin skin test (TST)	Patients with confirmed tuberculosis were enrolled from Seoul National University Hospital in Korea. Patients with a positive HIV test were excluded.	A group of healthy blood donors with normal chest X-ray and without respiratory symptoms and signs were recruited from medical students and employees of Seoul National University College of Medicine/Seoul National University Hospital.	The frequency of the rs2569190 T allele was higher in tuberculosis patients than in healthy controls (64% vs. 57%; p = 0.01), and rs2569190 TT genotypes (OR= 1.60; 95% CI, 1.01–2.54) were over-represented among tuberculosis patients (43% vs. 32%; p = 0.016).
Druszczyñska et al., 2006 [29]	Poland	Case control	Caucasian Polish	**CD14** rs2569190 C>T	Pulmonary TB	126 TB and 122 controls; N = 248	Risk of *Mtb* diseases	Healthy volunteers who had no past history of TB	Not reported	Not reported	Not reported	No association was found between the rs2569190 and the presence of TB.
Pacheco et al., 2004 [12]	Colombia	Case control	Caucasian and Mestizo Colombian	**CD14** rs2569190 C>T	Pulmonary and extrapulmonary TB	267 TB and 112 controls; N = 379	Risk of *Mtb* diseases	Healthy control individuals were recruited	Tuberculin skin test (TST)	Patients were recruited from different health units in the metropolitan area of Medellin, Colombia. Individuals, who were HIV+, or with a history of cancer, autoimmune, metabolic, or endocrine diseases, as well as pregnant women, were excluded from the study.	Tuberculin-positive healthy control individuals were recruited from the Facultad de Medicina at the Universidad de Antioquia, and the institutions from where the patients were recruited.	No association was found between the allele and genotype frequencies and the presence of TB or between the different forms of the disease.
Hall et al., 2015 [30]	Uganda	Case control	USA	**NOD2** rs6500328 A>G, rs2111234 G>A and rs17313265 C>T	TB^a^	240 TB 595 controls; N= 835	Risk of *Mtb* diseases	Healthy household contacts without active disease were included in the control group	Tuberculin skin test (TST)	Analysis was gathered from two phases of a household contact study conducted in Kampala, Uganda. Subjects from the Household Contact Study were enrolled from 1995 to 1999. Individuals who presented at the study clinic with active culture-positive pulmonary TB were enrolled as index cases.	Analysis was gathered from two phases of a household contact study conducted in Kampala, Uganda. Subjects from the Household Contact Study were enrolled from 1995 to 1999. Healthy household contacts underwent a follow-up evaluation every 3 months for the first 6 months and were enrolled as control	rs17313265 association with TB in adults (examination of age-specific effects with TB) (OR= 2.82, 95% CI= 1.05–7.53). rs6500328 (OR= 2.44, 95% CI=1.01–5.88) and rs2111234 (OR= 1.56 95% CI= 1.07–2.28) showed a nominal association with resistance to *Mycobacterium tuberculosis* (Mtb) infection.
Zhao et al., 2012 [31]	China	Case control	Chinese Han, Uygur and Kazak	**NOD2** rs1861759 T>G	Pulmonary	425 TB and 380 controls; N=805	Risk of *Mtb* diseases	Healthy controls were HIV negative and none was known to present any autoimmune, chronic inflammatory or any other disease conditions.	Not reported	Han population 219 PTB and 215 healthy controls; For the Uygur population 86 PTB patients and 72 controls; for the Kazak 120 PTB patients and 93 healthy controls. The patients were diagnosed based on the TST test, chest X-ray, or sputum smear culture results.	They were HIV-negative patients and controls without any auto immune, chronic inflammatory, or any other disease condition. All selected patients had no mixed descendants within 3 generations.	By comparing the TG genotype frequencies of rs1861759 in the Han population, a significant difference was observed between the patients with TB and the healthy controls (OR= 2.16; 95% CI= 1.31–3.58; p= 0.0023).
Pan et al., 2012 [32]	China	Case control	Chinese Han	**NOD2** rs7194886 C>T and rs9302752 T>C	Pulmonary	1043 TB and 808 controls; N= 1851	Risk of *Mtb* diseases	The controls were selected from a pool of individuals who participated in the local community-based health examination programs. None of controls had a history of active tuberculosis and/or a malignancy	Not reported	Patients older than 15 years, Han Chinese. They were divided in 3 groups: clinical symptoms of TB, sputum smear positive, and bacteriologically confirmed TB. 234 of 1043 TB patients were sputum smear negative.	Every control was older than 15 years, without history of TB and/or a malignancy.	The individuals carrying the CT/TT genotype of rs7194886 had an increased risk of pulmonary tuberculosis (OR= 1.35, 95% CI= 1.05–1.72). Allele frequency analysis found that variant allele T of rs7194886 (OR= 1.25, 95% CI= 1.00–1.57) was associated with an increased risk of tuberculosis. Haplotype rs9302752 C–rs7194886 T was associated with an increased risk of being sputum culture-positive tuberculosis (p = 0.039).
Austin et al., 2008 [33]	USA	Case control	African Americans	**NOD2** rs2066842 C>T rs2066844 C>T, and rs5743278 C>G	Pulmonary and extrapulmonary TB	377 TB and 187 controls; N = 564	Risk of *Mtb* diseases	Control subjects for this study were recruited without a history of TB, autoimmune disease, or other infectious diseases	Tuberculin skin test (TST)	All cases patients were HIV negative and had their ethnicities determined by self-indication. TB diagnosis was given based on bacille culture (286/312 PTB cases and 33/43 of EPTB); in these negative patients, diagnosis was based on clinical manifestations, chest X-ray, and clinical improvement to antimycobacterial treatment.	African Americans without history of TB, autoimmune disease, or other diseases.	Minor allele carriers (heterozygous and homozygous) of rs2066842 (OR= 0.55, 95% CI=0.32–0.94, p= 0.02) and rs2066844 (OR= 0.27, 95% CI= 0.08–0.88; p= 0.01) presented decreased risks for TB disease. Conversely, the minor allele carrier (heterozygous) of rs2066844 (OR= 2.16, 95% CI= 1.10–4.72; p= 0.03) showed an increased risk for TB disease.

Abbreviations: *NOD2* nucleotide-binding oligomerization domain-containing protein 2, *CD14* Cluster Differentiation antigen 14, *TB* tuberculosis, *PTB* pulmonary tuberculosis, *EPTB* extrapulmonary tuberculosis, *OR* odds ratio, *CI* confidence intervals, *USA* United States of America

^a^Did not specify the TB type

In this systematic review, data on 4054 TB patients were examined, whereas 3993 individuals were identified as controls. The median sample size (IQR) per study was 267 (123–401) and 187 (127–413) for TB patients and healthy controls, respectively. The detailed characteristics of each study are shown in Table 1.

**Fig. 2:**
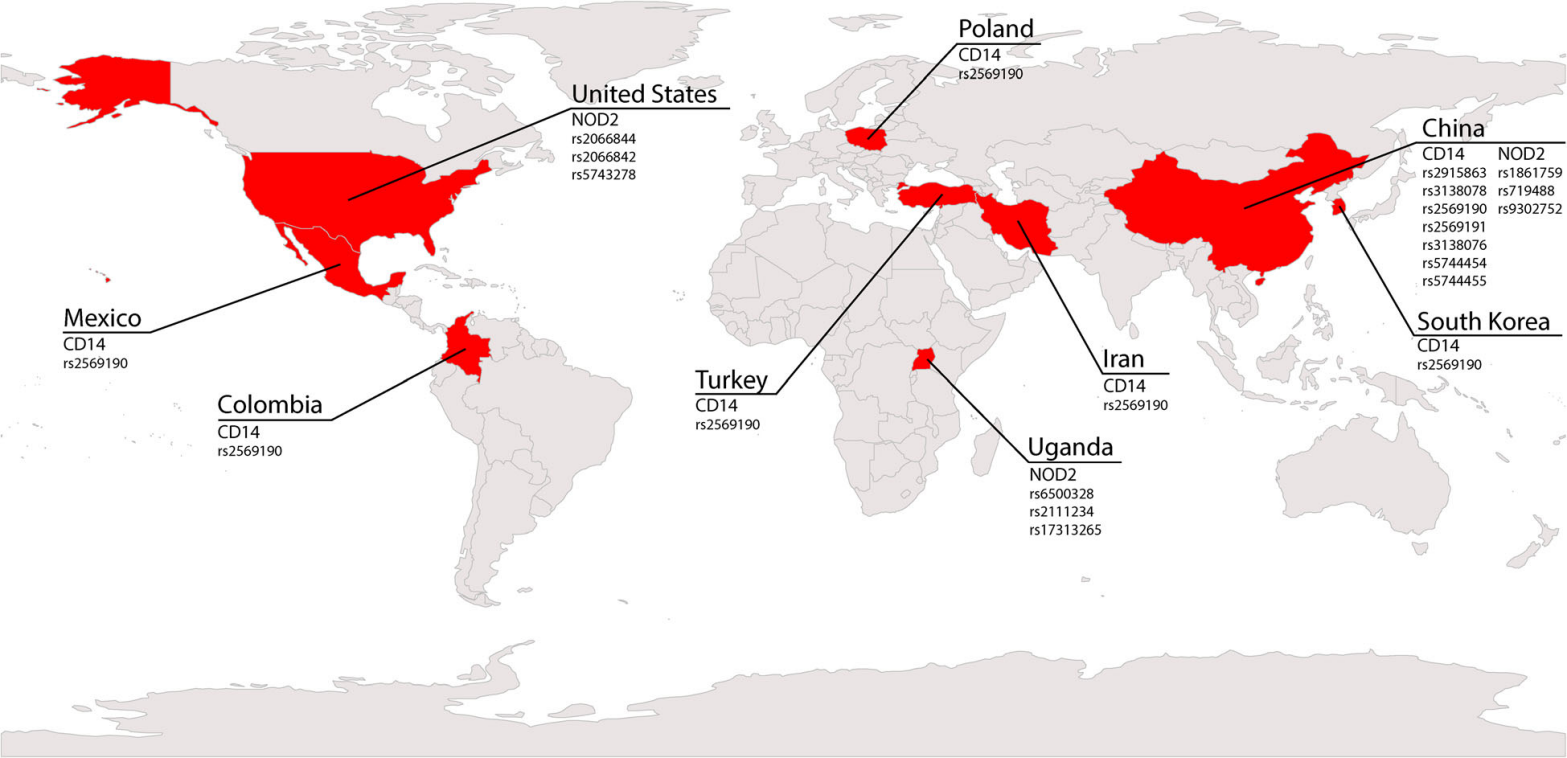
Geographical distribution of CD14 and NOD2 SNPs associated with risk of *Mtb* diseases. Studies originated from nine different countries. Most studies were from Asia (n=8), with China leading as study site (n=5) followed by Iran (n= 1), Turkey (n= 1), and South Korea (n= 1). Three studies were performed in the American continent: one in the USA, one in Mexico, and one in Colombia. Only one study was set in Europe (Poland), and one was performed in Africa (Uganda)

As observed in Fig. 2, most studies originated from Asia (n= 8) [23–28, 31, 32], with China leading as the most frequent study site (n= 5) [23–25, 31, 32], followed by Turkey (n= 1) [27], Iran (n= 1) [26], and South Korea (n= 1) [28]. The American continent also contributed to studies (n= 3): 1 in the USA [33], 1 in Mexico [4], and 1 in Colombia [12]. Only one study was set in Africa, specifically in Uganda [30], and Europe was represented by one study from Poland [29]. It is also possible to visualize in Fig. 2 that the *CD14* polymorphisms were studied in diverse populations from various ethnicities, including Mexican, Colombian, Polish, Turkish, Iranian, South Korean, and Chinese. In contrast, the *NOD2* polymorphisms were studied in 3 restricted populations: North Americans, Chinese, and Ugandan.

### Quality assessment and sensitivity analysis

The quality scores of the studies, assessing the risk of bias, are displayed in Table 2. All the studies were of moderate quality (Table 2). Of note, 5 studies [12, 27, 28, 30, 33] were clear about the procedures used to test the control populations (persons without TB infection/exposure), affecting the quality score with regard to comparability between the experimental groups (Table 2).

**Table 2:** Quality assessment of studies included in the systematic review by Newcastle-Ottawa Scale

N°	Source	Selection				Comparability		Exposure			Overall score^**a**^
		1	2	3	4	5A	5B	6	7	8	
**1**	Zhao et al. [25]	*	*	0	0	*	NA	*	*	NA	5
**2**	Kang et al. [28]	*	*	*	0	*	NA	*	*	NA	6
**3**	Alavi-Naini et al. [26]	*	*	0	0	*	NA	*	*	NA	5
**4**	Zhao et al. [31]	*	*	*	0	*	NA	*	*	NA	6
**5**	Rosas-Taraco et al. [4]	*	*	*	0	*	NA	*	*	NA	6
**6**	Pacheco et al. [12]	*	*	*	0	*	NA	*	*	NA	6
**7**	Austin et al. [33]	*	*	0	0	*	NA	*	*	NA	5
**8**	Zheng et al. [23]	*	*	0	0	*	NA	*	*	NA	5
**9**	Xue et al. [24]	*	*	*	0	*	NA	*	*	NA	6
**10**	Hall et al. [30]	*	*	*	0	*	NA	*	0	NA	5
**11**	Pan et al. [32]	*	0	*	0	*	NA	*	*	NA	5
**12**	Ayaslioglu et al. [27]	*	*	*	0	*	NA	*	*	NA	6
**13**	Druszczyñska et al. [29]	*	0	0	0	*	NA	*	*	NA	4

*Abbreviations*: *NA* not applicable

Star (*) indicates the score given to the study according to the NOS quality assessment scale

^a^Determined by the total number of stars assigned to study: 0–3 stars = poor; 4–6 stars = moderate; ≥7 stars = good quality

We performed sensitivity analysis with five studies [12, 27, 28, 30, 33] that specifically mentioned use of tuberculin skin test to exclude TB in the control groups. The five studies investigated different SNPs, and only 3 studies [12, 27, 28] reported results from the same SNP (rs2569190). A summary of the observations is illustrated in Additional File 2. Hence, a sensitivity analysis could not be performed for the other SNPs.

### *CD14* polymorphisms and risk *of Mtb* diseases

In the present review, *CD14* polymorphisms were the most frequently studied in the context of the risk of *Mtb* diseases. In total, 9 studies reported potential associations, presenting data for different populations. The sample sizes in total were 1976 TB cases and 2011 controls. The studies reported data on 7 *CD14* SNPs: rs2569190 [4, 12, 23, 24, 26–29, 31], rs2569191 [24, 31], rs3138078 [24], rs2915863 [24], rs3138076 [24], rs5744455 [24], and rs5744454 [24].

#### Linkage disequilibrium

The study of Xue et al. [24] was the only one that evaluated the magnitude of linkage disequilibrium and found that rs2569191 and rs2569190 were in high linkage disequilibrium (R^2^ = 0.90). We next examined effects of linkage disequilibrium and SNP-SNP interactions in the CD14 gene with the Package LDpop as described in the “Methods” section [35]. The SNPs rs2569190-rs2569191 (R^2^ = 0.9715), rs3138078-rs3138076 (R^2^ = 0.9924), rs3138078-5744454 (R^2^ = 0.9924), and rs3138076-rs5744454 (R^2^ = 1) were in high linkage disequilibrium and represented a haplotype block. The tables with all values of linkage disequilibrium for all SNPs can be found in supplementary information Additional File 3.

The findings on linkage disequilibrium are described below narratively for each SNP of CD14 identified in the search from here onwards as subsections. Likewise, given the heterogeneity in populations and SNPs, only a narrative description was feasible.

#### SNP rs2569190

Of the 9 publications which investigated this gene locus, six studies reported a significant association between the T allele of SNP rs2569190 and higher odds of TB [4, 23, 24, 26, 28, 31].

Zhao et al. identified the T allele as the major allele of the cases that was higher in TB cases compared to healthy controls (63.53% vs 55.44%, respectively) [31]. Hence, the frequencies of the allele C in the rs2569190 polymorphism were lower in TB cases than in controls suggesting that CT and CC genotypes are likely protective against TB (OR = 0.46 and 0.63, respectively) [31]. Similarly, Alavi-Naini et al. observed that the risk *of Mtb* diseases was greater in individuals with the T-allele (CT and TT) than in those without, finding that the T allele was more common in TB patients (57%) than in controls (44%) [26]. Moreover, in this same study, the C allele in homozygosis was a protective factor in a sub-analysis of Iranian subjects, with an OR of 0.44 (95% CI 0.23–0.83; p= 0.006) [26].

Zheng et al. [23] found that the frequency of the rs2569190 T allele was significantly higher in spinal TB patients compared to healthy controls (57.5% v 44%; p<0.01), demonstrating that those with TT and CT genotypes was more frequent in spinal TB patients than in healthy controls (85% vs 44.17%; p<0.05) [23]. In contrast, Rosas-Taraco et al. [4] found that the most frequent allele of the rs2569190 was allele C in all study population, but the highest frequency of the rs2569190 T allele was 71% in household contacts of TB index cases who developed active TB, and 60% in those with pulmonary TB. In contrast, this allele was present in only 40% the healthy controls and 39.2% in the household contacts without TB (p < .0001) [4].

Xue et al. [24] observed allele T as the common allele of rs2569190 in the study population [24]. The TT genotype of rs2569190 was significantly associated with increased risk of *Mtb* diseases, present in 46% in patients diagnosed with pulmonary TB and 30% in healthy controls (p<0.001) [24]. Finally, Kang et al. [28] identified that the TT genotypes increased the risk *of Mtb* diseases and was significantly more frequent in TB patients than in healthy controls (43% vs 32%; p = 0.016) [28].

Notably, two articles, by Ayaslioglu et al. [27] and Pacheco et al. [12], described more reliably “control” groups; in such studies, the authors found no statistically significant difference between the presence of SNP rs2569190 and increased TB risk. Moreover, another study [29] also did not find any evidence of a significant association between SNP rs2569190 and TB development. Furthermore, no association was found between the *CD14*-159C/T polymorphism and TB clinical severity in studies that evaluated Turkish [27], Caucasian Polish [29], or White and Mestizo Colombian patients [12]. In the Turkish study performed by Ayaslioglu et al. [27], one hypothesis for the lack of association was the small sample size (88 TB cases and 116 controls). While investigating Caucasian Polish individuals, Druszczyñska et al. [29] did not specify whether TB patients and controls were from the same region, which could possibly account for the lack of association.

#### SNP rs2569191

Additional investigations evaluating the CD14 SNP rs2569191 revealed significant associations with odds of TB in 2 distinct publications from China [24, 31]. In one of these studies [24], individuals with the GG genotype of A-1145G were more likely to present with TB (p<0.001), and this genotype was more common in the patients diagnosed with pulmonary TB (46%) than the healthy controls (28%). The second investigation [31] suggested that the frequencies of genotypes AG and AA were lower in TB cases compared to healthy controls [AG=181 (41.90%) vs 174 (47.41%) and AA= 65 (15.05%) vs 85 (23.16%) respectively], arguing for a protective role against TB and not found significance with the GG genotype. Both studies reported that the G allele of A-1145G was more prevalent in TB cases than in controls, indicating an increased risk *of Mtb* diseases.

#### Other SNPs

*CD14* SNPs (rs3138078, rs2915863, rs2569192, rs3138076, rs5744455, and rs5744454) were evaluated by only one study [24], finding the following alleles to be significantly associated with TB: G allele of rs2915863 and the G allele of rs3138078. For the SNPs rs2569192, rs3138076, rs5744455, and rs5744454, there were no statistically relevant associations

### *NOD2* polymorphisms and risk *of Mtb* diseases

In regard to the *NOD2* gene, the results from the studies were diverse, with SNPs associated with either an increased or decreased risk *of Mtb* diseases in each of the study populations based on ethnicity, age group, or biological sex. In such studies, a total of 2085 TB cases and 2347 controls were investigated. These studies were performed in different countries including China, Uganda, and North America, with the last being focused on African Americans. Two publications reported data on *NOD2* SNPs in China [25, 32], with a total of 2651 individuals.

#### Linkage disequilibrium

The study of Hall et al. [30] used the linkage disequilibrium for selected SNPs with linkage disequilibrium R2 ≥ 0.8. The other study that used linkage disequilibrium was Pan et al. [32]. In such investigation, the selected haplotype blocks where haplotype rs9302752C–rs7194886T (block 1) which was associated with an increased risk of being a case of sputum culture-positive tuberculosis. We next examined the effects of linkage disequilibrium and SNP-SNP interactions in the NOD2 gene with the Package LDpop [35]. Here, we found that no reported SNP was in high linkage disequilibrium. The tables with all values of linkage disequilibrium for all SNPs can be found in supplementary information Additional File 4.

Hereafter, the findings on linkage disequilibrium will be reported narratively for each NOD2 SNP identified in the search as subsections. Once again, given the heterogeneity in populations and SNPs, only a narrative description was feasible.

#### SNP rs1861759

Zhao et al. [25] showed that in the Han population, the T allele frequency of the SNP rs1861759 in the patient group and in the control group was the most common. The TG genotype in rs1861759 SNP was substantially associated with TB (p = 0.0023) in the Han population with a frequency of 53 (24.2%) in pulmonary TB patients and 28 (13%) in healthy controls. Interestingly, the same study did not identify this relationship between the TG genotype and TB in Chinese participants of Kazak or Uygur ancestry.

#### SNP rs7194886

A different study by Xue et al. investigating Han Chinese individuals [24] found that the frequency of the rs7194886 T allele was associated with risk *of Mtb* diseases (p=0.042), but allele C was the most common in all study subjects. Patients who presented CT or TT genotypes of rs7194886 were more likely to present with TB when compared to individuals with the CC genotype (p=0.018). The genotype CT was more frequent in the pulmonary TB case compared with uninfected controls (CT= 271 [26.03%] vs 179 [22.43%]). Furthermore, the study found a higher frequency of rs7194886 polymorphism in either smokers (p=0.019) or men (p=0.014) [28].

#### SNP rs9302752

In the same study [32], the haplotype rs9302752 C–rs7194886 T was linked with a higher risk of presenting with sputum culture-positive TB (p = 0.039).

#### SNPs rs2066842, rs2066844, and rs5743278

The study by Austin et al., which predominantly recruited African Americans in the USA [33], found C allele of the SNPs rs2066842, rs2066844, and rs5743278 in all case patients and control subjects. This study reported an association between NOD2 SNPs rs2066842, rs2066844, and rs5743278 and odds of TB. The study participants who were carriers of the CC genotype in SNPs rs2066842 (p=0.02) and rs2066844 (p=0.01) were less likely to have TB. The frequencies of the CC genotype (340 vs 156) in rs2066842 and in rs2066844 (372 vs 178) were higher in patients with TB compared with control subjects. Moreover, individuals who were heterozygous (CG) in rs5743278 were more frequent in those with TB compared to the control group (39 vs 10) and exhibited an increased chance of having TB (p=0.03).

#### *SNPs* rs17313265, *rs6500328*, *and rs2111234*

The study by Hall et al. in the adult African population from Uganda [30] reported that presence of the SNP rs17313265 was associated with increased risk *of Mtb* diseases (OR= 2.82, 95% CI= 1.05, 7.53; p= 0.0052). Notably, increased frequency of the rs6500328 and rs2111234 SNPs was found in those without TB, suggesting that such SNPs may protect against Mtb infection, with OR of 2.44 (95% CI= 1.01. 5.88; p= 0.047) and 1.56 (95% CI= 1.07, 2.28, p=0.020), respectively.

## Discussion

The molecular basis of immune response to infectious diseases is an indispensable approach to understand how gene regulation ultimately may impact clinical outcomes. In recent decades, a great deal of research has sought to identify associations between genetic polymorphisms and risk *of Mtb* diseases [36, 37]. Most of the target loci are PRRs contributing to control of mycobacterial diseases, especially TB [38]. *CD14* and *NOD2* are considered to be key PRRs in the innate immune system [9]. In this systematic review, a variety of studies were identified evaluating seven *CD14* SNPs and four evaluating nine *NOD2* SNPs, finding several genetic variants associated with Mtb infection.

We identified studies that found significant association between the T allele of *CD14* SNP rs2569190 and increased risk *of Mtb* diseases in different ethnic groups. This polymorphism is located in the promoter region of the *CD14* gene [9], and the T allele seems to act as a negative regulator of in vitro T-cell proliferation and decreased production of cytokines, including interferon-y (IFN-γ) [28]. IFN-γ is an essential cytokine for the control of mycobacterial infection [39], and therefore, rs2569190 may produce an environment that favors development of TB. Interestingly, other studies found this SNP associated with ischemic stroke [40], cardiovascular disease [41], and asthma [42], indicating that these polymorphisms could actually lead to a more profound alteration in immune responses that may affect a large number of clinical conditions.

Another important association of *CD14* polymorphism with TB was described in this review, involving the SNP rs2569191. In this setting, the G allele of A-1145G was more prevalent in TB cases than in controls in two Chinese studies. An interesting link between such SNP and circulating concentrations of IgE has been proposed. In a study performed in patients with asthma, the authors identified heightened IgE concentrations in those who had the G allele of A-1145G [43]. Moreover, other studies identified IgE as a marker of Mtb infection, in which pre-treatment levels of serum total IgE concentrations in TB patients were significantly higher than in healthy individuals; such levels decreased after successful antitubercular treatment [44, 45]. It is possible that the polymorphism rs2569191 plays an important role in TB infection which may be related to IgE concentrations. Future studies in other ethnic groups are warranted to directly test this hypothesis. Interestingly, the two SNPs rs2569191 and rs2569190 are found to be in linkage disequilibrium [46]. For this reason, future studies will need to determine whether these SNPs influence risk *of Mtb* diseases individually when solely associated.

*NOD2* is one of the most well-studied genes in the context of the innate immune response against microbial pathogens [47]. This gene accounts for a cytoplasmic receptor belonging to the NOD-like receptor family [48] and is known to participate in the induction of inflammation during Mtb infection [49]. In experimental conditions, Mtb recognition is *NOD2*-dependent [50]. Mice genetically deficient in *NOD2* are shown to be more susceptible to TB [51]. Here, we found two studies [25, 32] in the Chinese population that reported three different polymorphisms (rs1861759, rs7194886, and rs9302752) associated with increased risk *of Mtb* diseases. The rs1861759 (synonymous variant) TG genotype is associated with increased risk *of Mtb* diseases [25]. Individuals with the rs7194886 CT or TT genotype are more likely to develop TB [32]. The rs9302752 C allele has been linked to a higher risk of being sputum culture-positive TB. To our knowledge, these three polymorphisms do not appear in other TB studies. Curiously, such SNPs have been also related to increased risk of leprosy [52].

In an African-American study [33], it was observed that the three *NOD2* polymorphisms exhibit impact on risk *of Mtb* diseases. The polymorphism rs2066844 represents missense mutations whose variants are located in the C-terminal region and cause defective production of proinflammatory mediators [53]. The rs2066842 is a missense variant but, when presented alone, does not alter gene function [54]. Importantly, the two polymorphisms in our systematic review appear to protect against TB when in the presence of allele C [33]. In contrast, the presence of the T allele in such mutations was associated with increased risk *of Mtb* diseases. The association between the presence of the T allele and augmented pathology has been described for other diseases such as Crohn’s disease [55] and gastric cancer [56]. The genotyping heterozygous (CG) of rs5743278 has been linked to higher risk of *Mtb* diseases [55]. It is possible that the rs5743278 SNP causes an amino acid change from a low hydrophobic arginine to a highly hydrophobic tryptophan, modifying the stability of the *NOD2* structure or its ability to properly interact with Mtb ligands [55]. Finally, one study in a population from Uganda described the *NOD2* rs17313265 polymorphism associated with increased risk *of Mtb* diseases in adults whereas the SNPs rs6500328 and rs2111234 exhibited decreased risk *of Mtb* diseases [30]. Furthermore, these three polymorphisms have not been reported in other studies with TB patients. These findings indicate that NOD2 polymorphisms may be dramatically affected by ethnicity and/or ancestry. This scenario reinforces the need of additional investigations performed in a variety of ethnic populations, particularly when study design uses family-based control subjects to eliminate the bias in stratification of the populations.

To our knowledge, the present study is the first systematic review to explore the relation of all *CD14* and *NOD2* SNP polymorphisms with the risk *of Mtb* diseases in different ethnicities. A strength of our study was the comprehensive search strategy, which used detailed inclusion and exclusion criteria. Moreover, methodological quality was assessed in duplicate using the Newcastle-Ottawa scale [34], which reduced the subjectivity of the selection of studies and allowed for precise evaluation of the risk of bias in several domains. There was no report categorized as low quality, resulting in a review with reduced risk of bias. Another important strength is that the inclusion of the SNPs was not limited to one specific locus or ethnicity, allowing the study to observe different loci associated with risk *of Mtb* diseases or not in various populations.

Our study has some limitations. It was not possible to perform a meta-analysis and, consequently, a sensitivity analysis, mainly because a considerable part of the SNP polymorphisms appeared in only one study at a time. Only two SNPs, rs2569191 which was seen in two studies and rs2569190 which was reported in nine publications were consistently investigated. However, the need for a study to compile and critically revise these results in different ethnicities reinforces the importance of our work, regardless of quantitative analyses. Another limitation was the moderate quality of those studies included in the systematic review. One reason for this quality is the ambiguous concept of the control groups reported in most of the studies where the precise screening method to categorize these groups was not specifically described. To circumvent such concern, we have contacted the authors individually but obtained replies from just two articles. Only 5 of the 13 included studies reported active TB screening of asymptomatic individuals with either tuberculin skin test (TST) or interferon-gamma releasing assay (IGRA). This imprecision in the definition of control groups may limit the full interpretation of the study results. Our study highlights the need of studies standardizing description of the control groups to more accurately delineate the associations between SNPs and risk *of Mtb* diseases. Other determinants for the moderate quality were the small sample size of some studies and lack of clarity regarding description of the tools/approaches used to choose the SNPs, such as the examination of the linkage disequilibrium.

This review identified multiple studies that determined an association of the minor allele T at position chr5:140633331 of rs2569190 CD14 polymorphism with increased risk *of Mtb* diseases in persons from different ethnicities. In addition, the *CD14* SNP rs2569191 and the *NOD2* SNPs rs1861759 and rs7194886 are shown here to be associated with a high risk *of Mtb* diseases in the Chinese population. In contrast, genotypes CG or GG of rs2066842 and rs2066844 were at low risk *of Mtb* diseases in African Americans. Since such genes account for key molecules of the immune system, the referred polymorphisms of *CD14* and *NOD2* genes likely play an important role in TB physiopathology. These results add knowledge to the field by reinforcing the genetic influence on the risk *of Mtb* diseases. Such knowledge, if validated by larger studies, may help development of tools for assessment of the risk *of Mtb* diseases and hopefully predict clinical outcomes in precision medicine approaches.

## Supplementary Information

**Additional file 1.** Table with the exact search strategy per database and the number of hits per database.

**Additional file 2.** Table of the five studies that evaluated the control groups with the tuberculin skin test.

**Additional file 3.** Tables describing the frequencies and the linkage disequilibrium of the seven CD14 SNPs.

**Additional file 4.** Tables depicting the frequencies and the linkage disequilibrium of the nine NOD2 SNPs.

## Data Availability

All data used in the present study were retrieved from the publications used in the systematic review and are publicly available.

## Abbreviations

MONSTER: Multinational Organization Network Sponsoring Translational and Epidemiological Research
UNIFACS: Universidade Salvador
FTC: Faculdade de Tecnologia e Ciências
TB: Tuberculosis
SNP: Single nucleotide polymorphisms
NOQS: Newcastle-Ottawa Quality Scale
Mtb: *Mycobacterium tuberculosis*
*NOD2*: Domain containing protein 2
CD14: Cluster differentiation antigen 14
PRR: Pattern recognition receptor
TLRs: Toll-like receptors
MDP: Muramyl dipeptide
NFkB: Nuclear factor kappa-B
PRISMA: Preferred Reporting Items for Systematic Reviews and Meta-Analyses
SciELO: Scientific Electronic Library Online
Lilacs: Literatura Latino-Americana e do Caribe em Ciências da Saúde
IQR: Median sample size
OR: Odds ratio
IFN-γ: Interferon-y
CNPq: *Conselho Nacional de Desenvolvimento Científico e Tecnológico*
OAS-PAEC: Organization of American States - Partnerships Program for Education and Training
CAPES: *Coordenação de Aperfeiçoamento de Pessoal de Nível Superior*—Brazil
FAPESB: *Fundação de Amparo à Pesquisa da Bahia*
PTB: Pulmonary tuberculosis
EPTB: Extrapulmonary tuberculosis
CI: Confidence intervals
TST: Tuberculin skin test
IGRA: Interferon-gamma releasing assay
USA: United States of America
NA: Not applicable

## References

[1] WHO. WHO: Global tuberculosis report 2019: WHO; 2019.

[2] Zhai W, Wu F, Zhang Y, Fu Y, Liu Z. The immune escape mechanisms of Mycobacterium tuberculosis. Int J Mol Sci. 2019;20(2):340. 10.3390/ijms20020340 PMC6359177 30650615

[3] van Tong H, Velavan TP, Thye T, Meyer CG (2017). Human genetic factors in tuberculosis: an update. Trop Med Int Health.

[4] Rosas-Taraco AG, Revol A, Salinas-Carmona MC, Rendon A, Caballero-Olin G, Arce-Mendoza AY (2007). CD14 C(-159)T polymorphism is a risk factor for development of pulmonary tuberculosis. J Infect Dis.

[5] Fouad NA, Saeed AM, Mahedy AW (2019). Toll like receptor-4 gene polymorphism and susceptibility to pulmonary tuberculosis. Egypt J Immunol.

[6] Yi YX, Han JB, Zhao L, Fang Y, Zhang YF, Zhou GY (2015). Tumor necrosis factor alpha gene polymorphism contributes to pulmonary tuberculosis susceptibility: evidence from a meta-analysis. Int J Clin Exp Med.

[7] Kleinnijenhuis J, Joosten LA, van de Veerdonk FL, Savage N, van Crevel R, Kullberg BJ, van der Ven A, Ottenhoff TH, Dinarello CA, van der Meer JW (2009). Transcriptional and inflammasome-mediated pathways for the induction of IL-1beta production by Mycobacterium tuberculosis. Eur J Immunol.

[8] Elass E, Coddeville B, Guerardel Y, Kremer L, Maes E, Mazurier J, Legrand D (2007). Identification by surface plasmon resonance of the mycobacterial lipomannan and lipoarabinomannan domains involved in binding to CD14 and LPS-binding protein. FEBS Lett.

[9] Azad AK, Sadee W, Schlesinger LS (2012). Innate immune gene polymorphisms in tuberculosis. Infect Immun.

[10] Bernardo J, Billingslea AM, Blumenthal RL, Seetoo KF, Simons ER, Fenton MJ (1998). Differential responses of human mononuclear phagocytes to mycobacterial lipoarabinomannans: role of CD14 and the mannose receptor. Infect Immun.

[11] Reiling N, Holscher C, Fehrenbach A, Kroger S, Kirschning CJ, Goyert S, Ehlers S (2002). Cutting edge: Toll-like receptor (TLR)2- and TLR4-mediated pathogen recognition in resistance to airborne infection with Mycobacterium tuberculosis. J Immunol.

[12] Pacheco E, Fonseca C, Montes C, Zabaleta J, Garcia LF, Arias MA (2004). CD14 gene promoter polymorphism in different clinical forms of tuberculosis. FEMS Immunol Med Microbiol.

[13] Jiao D, Wong CK, Qiu HN, Dong J, Cai Z, Chu M, Hon KL, Tsang MS, Lam CW (2016). NOD2 and TLR2 ligands trigger the activation of basophils and eosinophils by interacting with dermal fibroblasts in atopic dermatitis-like skin inflammation. Cell Mol Immunol.

[14] Landes MB, Rajaram MV, Nguyen H, Schlesinger LS (2015). Role for NOD2 in Mycobacterium tuberculosis-induced iNOS expression and NO production in human macrophages. J Leukoc Biol.

[15] Girardin SE, Boneca IG, Viala J, Chamaillard M, Labigne A, Thomas G, Philpott DJ, Sansonetti PJ (2003). Nod2 is a general sensor of peptidoglycan through muramyl dipeptide (MDP) detection. J Biol Chem.

[16] Genetics Home Reference [https://ghr.nlm.nih.gov/.] Accessed 3 Jan 2021.

[17] Bai X, Feldman NE, Chmura K, Ovrutsky AR, Su WL, Griffin L, Pyeon D, McGibney MT, Strand MJ, Numata M (2013). Inhibition of nuclear factor-kappa B activation decreases survival of Mycobacterium tuberculosis in human macrophages. PLoS One.

[18] Cai X, Xu Q, Zhou C, Zhou L, Dai W, Ji G (2019). The association of nucleotide-binding oligomerization domain 2 gene polymorphisms with the risk of asthma in the Chinese Han population. Mol Genet Genomic Med.

[19] Branquinho D, Freire P, Sofia C (2016). NOD2 mutations and colorectal cancer - where do we stand?. World J Gastrointest Surg.

[20] Sidiq T, Yoshihama S, Downs I, Kobayashi KS (2016). Nod2: a critical regulator of ileal microbiota and Crohn’s disease. Front Immunol.

[21] Ferwerda G, Girardin SE, Kullberg BJ, Le Bourhis L, de Jong DJ, Langenberg DM, van Crevel R, Adema GJ, Ottenhoff TH, Van der Meer JW (2005). NOD2 and toll-like receptors are nonredundant recognition systems of Mycobacterium tuberculosis. PLoS Pathog.

[22] Aravindan PP (2019). Host genetics and tuberculosis: theory of genetic polymorphism and tuberculosis. Lung India.

[23] Zheng M, Shi S, Wei W, Zheng Q, Wang Y, Ying X, et al. Correlation between MBL2/CD14/TNF-alpha gene polymorphisms and susceptibility to spinal tuberculosis in Chinese population. Biosci Rep. 2018;38(1):BSR20171140. 10.1042/BSR20171140 PMC5794501 29298876

[24] Xue Y, Zhao ZQ, Chen F, Zhang L, Li GD, Ma KW, Bai XF, Zuo YJ (2012). Polymorphisms in the promoter of the CD14 gene and their associations with susceptibility to pulmonary tuberculosis. Tissue Antigens.

[25] Zhao M, Jiang F, Zhang W, Li F, Wei L, Liu J, Xue Y, Deng X, Wu F, Zhang L, Zhang X, Zhang Y, Fan D, Sun X, Jiang T, Li JC (2012). A novel single nucleotide polymorphism within the NOD2 gene is associated with pulmonary tuberculosis in the Chinese Han, Uygur and Kazak populations. BMC Infect Dis.

[26] Alavi-Naini R, Salimi S, Sharifi-Mood B, Davoodikia AA, Moody B, Naghavi A (2012). Association between the CD14 gene C-159T polymorphism and serum soluble CD14 with pulmonary tuberculosis. Int J Tuberc Lung Dis.

[27] Ayaslioglu E, Kalpaklioglu F, Kavut AB, Erturk A, Capan N, Birben E (2013). The role of CD14 gene promoter polymorphism in tuberculosis susceptibility. J Microbiol Immunol Infect.

[28] Kang YA, Lee HW, Kim YW, Han SK, Shim YS, Yim JJ (2009). Association between the -159C/T CD14 gene polymorphism and tuberculosis in a Korean population. FEMS Immunol Med Microbiol.

[29] Druszczynska M, Strapagiel D, Kwiatkowska S, Kowalewicz-Kulbat M, Rozalska B, Chmiela M, Rudnicka W (2006). Tuberculosis bacilli still posing a threat. Polymorphism of genes regulating anti-mycobacterial properties of macrophages. Pol J Microbiol.

[30] Hall NB, Igo RP, Malone LL, Truitt B, Schnell A, Tao L, Okware B, Nsereko M, Chervenak K, Lancioni C (2015). Polymorphisms in TICAM2 and IL1B are associated with TB. Genes Immun.

[31] Zhao MY, Xue Y, Zhao ZQ, Li FJ, Fan DP, Wei LL, Sun XJ, Zhang X, Wang XC, Zhang YX, Li JC (2012). Association of CD14 G(-1145)A and C(-159)T polymorphisms with reduced risk for tuberculosis in a Chinese Han population. Genet Mol Res.

[32] Pan H, Dai Y, Tang S, Wang J (2012). Polymorphisms of NOD2 and the risk of tuberculosis: a validation study in the Chinese population. Int J Immunogenet.

[33] Austin CM, Ma X, Graviss EA (2008). Common nonsynonymous polymorphisms in the NOD2 gene are associated with resistance or susceptibility to tuberculosis disease in African Americans. J Infect Dis.

[34] Deeks JJ, Dinnes J, D'Amico R, Sowden AJ, Sakarovitch C, Song F, Petticrew M, Altman DG, International Stroke Trial Collaborative G, European Carotid Surgery Trial Collaborative G (2003). Evaluating non-randomised intervention studies. Health Technol Assess.

[35] Alexander TA, Machiela MJ (2020). LDpop: an interactive online tool to calculate and visualize geographic LD patterns. BMC Bioinformatics.

[36] Shen W, Xiao L, Li Y, Zhou D, Zhang W (2020). Association between polymorphisms in mannose-binding lectin 2 gene with pulmonary tuberculosis susceptibility. Hereditas.

[37] Wu S, Liu X, Chen L, Wang Y, Zhang M, Wang M, et al. Polymorphisms of TLR2, TLR4 and TOLLIP and tuberculosis in two independent studies. Biosci Rep. 2018;38(1):BSR20171140. 10.1042/BSR20193141 PMC7403954 32648572

[38] Yim JJ, Selvaraj P (2010). Genetic susceptibility in tuberculosis. Respirology.

[39] Jurcev-Savicevic A, Katalinic-Jankovic V, Mise K, Gudelj I (2012). The role of interferon-gamma release assay in tuberculosis control. Arh Hig Rada Toksikol.

[40] Wu YQ, Cheng SY, Xu XC, Li WC (2019). Association between CD14 rs2569190 C>T polymorphism and ischemic stroke susceptibility: a meta-analysis based on 5,277 subjects. Neuropsychiatr Dis Treat.

[41] Xu JJ, Liu KQ, Ying ZM, Zhu XW, Xu XJ, Zhao PP, Bai WY, Qiu MC, Zhang XW, Zheng HF (2019). Effect of CD14 polymorphisms on the risk of cardiovascular disease: evidence from a meta-analysis. Lipids Health Dis.

[42] Nieto-Fontarigo JJ, Salgado FJ, San-Jose ME, Cruz MJ, Casas-Fernandez A, Gomez-Conde MJ, Valdes-Cuadrado L, Garcia-Gonzalez MA, Arias P, Nogueira M (2018). The CD14 (-159 C/T) SNP is associated with sCD14 levels and allergic asthma, but not with CD14 expression on monocytes. Sci Rep.

[43] Vercelli D (2010). Gene-environment interactions in asthma and allergy: the end of the beginning?. Curr Opin Allergy Clin Immunol.

[44] Adams JF, Scholvinck EH, Gie RP, Potter PC, Beyers N, Beyers AD (1999). Decline in total serum IgE after treatment for tuberculosis. Lancet.

[45] Ohrui T, Zayasu K, Sato E, Matsui T, Sekizawa K, Sasaki H (2000). Pulmonary tuberculosis and serum IgE. Clin Exp Immunol.

[46] Munthe-Kaas MC, Torjussen TM, Gervin K, Lodrup Carlsen KC, Carlsen KH, Granum B, Hjorthaug HS, Undlien D, Lyle R (2010). CD14 polymorphisms and serum CD14 levels through childhood: a role for gene methylation?. J Allergy Clin Immunol.

[47] Schenk M, Mahapatra S, Le P, Kim HJ, Choi AW, Brennan PJ, Belisle JT, Modlin RL (2016). Human NOD2 recognizes structurally unique muramyl dipeptides from Mycobacterium leprae. Infect Immun.

[48] Toledo Pinto TG, Batista-Silva LR, Medeiros RCA, Lara FA, Moraes MO (2018). Type I interferons, autophagy and host metabolism in leprosy. Front Immunol.

[49] Franchi L, Park JH, Shaw MH, Marina-Garcia N, Chen G, Kim YG, Nunez G (2008). Intracellular NOD-like receptors in innate immunity, infection and disease. Cell Microbiol.

[50] Behr MA, Divangahi M (2015). Freund’s adjuvant, NOD2 and mycobacteria. Curr Opin Microbiol.

[51] Divangahi M, Mostowy S, Coulombe F, Kozak R, Guillot L, Veyrier F, Kobayashi KS, Flavell RA, Gros P, Behr MA (2008). NOD2-deficient mice have impaired resistance to Mycobacterium tuberculosis infection through defective innate and adaptive immunity. J Immunol.

[52] Zhang X, Yuan Z, Ji J, Li H, Xue F (2016). Network or regression-based methods for disease discrimination: a comparison study. BMC Med Res Methodol.

[53] Lesage S, Zouali H, Cezard JP, Colombel JF, Belaiche J, Almer S, Tysk C, O'Morain C, Gassull M, Binder V (2002). CARD15/NOD2 mutational analysis and genotype-phenotype correlation in 612 patients with inflammatory bowel disease. Am J Hum Genet.

[54] Bonen DK, Ogura Y, Nicolae DL, Inohara N, Saab L, Tanabe T, Chen FF, Foster SJ, Duerr RH, Brant SR, Cho JH, Nuñez G (2003). Crohn’s disease-associated NOD2 variants share a signaling defect in response to lipopolysaccharide and peptidoglycan. Gastroenterology.

[55] Hugot JP, Chamaillard M, Zouali H, Lesage S, Cezard JP, Belaiche J, Almer S, Tysk C, O'Morain CA, Gassull M (2001). Association of NOD2 leucine-rich repeat variants with susceptibility to Crohn’s disease. Nature.

[56] Rosenstiel P, Hellmig S, Hampe J, Ott S, Till A, Fischbach W, Sahly H, Lucius R, Folsch UR, Philpott D, Schreiber S (2006). Influence of polymorphisms in the NOD1/CARD4 and NOD2/CARD15 genes on the clinical outcome of Helicobacter pylori infection. Cell Microbiol.

