# Supplementary material for: The influence of single nucleotide polymorphisms of NOD2 or CD14 on the risk of Mycobacterium tuberculosis diseases: a systematic review
Source: Syst Rev. 2021 Jun 9;10:174. doi: 10.1186/s13643-021-01729-y (PMC8191055; doi:10.1186/s13643-021-01729-y)
Supplement: Supplementary file 1 — Additional file 1. Table with the exact search strategy per database and the number of hits per database. [file 13643_2021_1729_MOESM1_ESM.docx]

**Additional File 1**

The following Table show the exact search strategy per database and the number of hits per database.

| **Databases used** | **Field of research** | **Site** | **Initial search strategy** | **The number of hits** | **Last date of search** |
| --- | --- | --- | --- | --- | --- |
| PubMed® | Health / Biomedical | https://www.ncbi.nlm.nih.gov/pubmed | (((tuberculosis) AND (CD14 gene)) OR (NOD2 gene)) AND (single nucleotide polymorphism) | 244 | August 22, 2020 |
| Embase | Health / Biomedical | [https://www.embase.com](https://www.embase.com/) | ((tuberculosis OR 'mycobacterium tuberculosis') AND cd14 OR nod2) AND 'single nucleotide polymorphism' | 242 | August 22, 2020 |
| Scientific Electronic Library Online (SciELO) | Health / Biomedical | https://scielo.org | (tuberculosis) AND (CD14) OR (NOD2) AND (single nucleotide polymorphism) | 25 | August 22, 2020 |
| Literatura Latino-Americana e do Caribe em Ciências da Saúde (Lilacs) | Health / Biomedical | <https://lilacs.bvsalud.org/es/> | tuberculosis [Palavras] and polimorfismo [Palavras] and NOD2 [Palavras] or CD14 [Palavras] | 102 | August 22, 2020 |
