# Supplementary material for: The influence of single nucleotide polymorphisms of NOD2 or CD14 on the risk of Mycobacterium tuberculosis diseases: a systematic review
Source: Syst Rev. 2021 Jun 9;10:174. doi: 10.1186/s13643-021-01729-y (PMC8191055; doi:10.1186/s13643-021-01729-y)
Supplement: Supplementary file 2 — Additional file 2. Table of the five studies that evaluated the control groups with the tuberculin skin test. [file 13643_2021_1729_MOESM2_ESM.docx]

**Additional File 2**

The following Table show the summary of the five studies that evaluated the control groups with the tuberculin skin test.

| **Parameter** | **Study 1** | **Study 2** | **Study 3** | **Study 4** | **Study 5** |
| --- | --- | --- | --- | --- | --- |
| Refrence Number | 30 | 31 | 11 | 24 | 34 |
| Author/Year | *Ayaslioglu, E. et al,* 2012 | *Kang, Y. et al,* 2009 | *Pacheco, E. et al,* 2004 | *Hall, N. et al,* 2015 | *Austin, C. et al,* 2008 |
| Gene / studied SNP | **CD14** rs2569190 C>T | **CD14** rs2569190 C>T | **CD14** rs2569190 C>T | **NOD2** rs6500328 A>G, rs2111234 G>A and rs17313265 C>T | **NOD2** rs2066842 C>T rs2066844 C>T, and rs5743278 C>G |
| No. Control group | 116 | 422 | 112 | 595 | 187 |
| No. TB patients | 88 | 274 | 267 | 240 | 377 |
| Odds Ratio | **rs2569190** OR= 1.1122  95 % CI, 0.62 -2.0  p = 0.7218 | **rs2569190** OR= 1.60  95% CI, 1.01-2.54 p = 0.016 | **rs2569190** OR= 1.1779  95 % CI:, 0.85-1.63  p = 0.326 | **rs17313265** OR= 2.82  95% CI= 1.05-7.53  p=0.039  **rs6500328** OR= 2.44  95% CI=1.01-5.88  p=0.047    rs2111234 OR= 1.56  95% CI= 1.07-2.28  p=0.020 | **rs2066842** OR= 0.55  95% CI=0.32-0.94  p= 0.020  **rs2066844** OR= 0.27  95% CI= 0.08-0.88  p= 0.010 |
