# Supplementary material for: The influence of single nucleotide polymorphisms of NOD2 or CD14 on the risk of Mycobacterium tuberculosis diseases: a systematic review
Source: Syst Rev. 2021 Jun 9;10:174. doi: 10.1186/s13643-021-01729-y (PMC8191055; doi:10.1186/s13643-021-01729-y)
Supplement: Supplementary file 3 — Additional file 3. Tables describing the frequencies and the linkage disequilibrium of the seven CD14 SNPs. [file 13643_2021_1729_MOESM3_ESM.docx]

**Additional File 3**

The following Tables shows the frequencies of the seven CD14 SNPs: rs2569190, rs2569191, rs3138078, rs2915863, rs3138076 , rs5744455 and rs5744454. The Linkage Disequilibrium (LD) are expressed by the parameters R^2^ and calculate using Package LDpop.

**S1 Table.** Pairwise linkage disequilibrium (R^2^) for rs2569190 and rs2569191 CD14 SNPs.

| **Population** | **N** | **rs2569190 Allele Freq** | **rs2569191 Allele Freq** | **R^2^** |
| --- | --- | --- | --- | --- |
| ACB | 96 | A: 33.33%, G: 66.67% | C: 36.46%, T: 63.54% | 0.8714 |
| AFR | 661 | A: 30.11%, G: 69.89% | C: 32.3%, T: 67.7% | 0.8961 |
| ALL | 2504 | A: 46.88%, G: 53.12% | C: 47.48%, T: 52.52% | 0.9715 |
| AMR | 347 | A: 53.03%, G: 46.97% | C: 52.88%, T: 47.12% | 0.9827 |
| ASW | 61 | A: 25.41%, G: 74.59% | C: 26.23%, T: 73.77% | 0.9581 |
| BEB | 86 | A: 50.0%, G: 50.0% | C: 50.0%, T: 50.0% | 1 |
| CDX | 93 | A: 54.3%, G: 45.7% | C: 54.3%, T: 45.7% | 1 |
| CEU | 99 | A: 53.03%, G: 46.97% | C: 53.03%, T: 46.97% | 1 |
| CHB | 103 | A: 61.65%, G: 38.35% | C: 61.65%, T: 38.35% | 1 |
| CHS | 105 | A: 59.52%, G: 40.48% | C: 60.0%, T: 40.0% | 0.9804 |
| CLM | 94 | A: 44.15%, G: 55.85% | C: 43.62%, T: 56.38% | 0.9786 |
| EAS | 504 | A: 57.24%, G: 42.76% | C: 57.44%, T: 42.56% | 0.9919 |
| ESN | 99 | A: 23.74%, G: 76.26% | C: 25.25%, T: 74.75% | 0.9213 |
| EUR | 503 | A: 48.61%, G: 51.39% | C: 48.61%, T: 51.39% | 1 |
| FIN | 99 | A: 38.38%, G: 61.62% | C: 38.38%, T: 61.62% | 1 |
| GBR | 91 | A: 46.15%, G: 53.85% | C: 46.15%, T: 53.85% | 1 |
| GIH | 103 | A: 58.25%, G: 41.75% | C: 58.25%, T: 41.75% | 1 |
| GWD | 113 | A: 26.55%, G: 73.45% | C: 29.65%, T: 70.35% | 0.8578 |
| IBS | 107 | A: 51.4%, G: 48.6% | C: 51.4%, T: 48.6% | 1 |
| ITU | 102 | A: 50.0%, G: 50.0% | C: 50.0%, T: 50.0% | 1 |
| JPT | 104 | A: 53.37%, G: 46.63% | C: 53.37%, T: 46.63% | 1 |
| KHV | 99 | A: 57.07%, G: 42.93% | C: 57.58%, T: 42.42% | 0.9796 |
| LWK | 99 | A: 42.93%, G: 57.07% | C: 43.43%, T: 56.57% | 0.9393 |
| MSL | 85 | A: 25.29%, G: 74.71% | C: 28.82%, T: 71.18% | 0.8361 |
| MXL | 64 | A: 54.69%, G: 45.31% | C: 55.47%, T: 44.53% | 0.9689 |
| PEL | 85 | A: 64.71%, G: 35.29% | C: 64.12%, T: 35.88% | 0.9747 |
| PJL | 96 | A: 55.73%, G: 44.27% | C: 55.73%, T: 44.27% | 1 |
| PUR | 104 | A: 50.48%, G: 49.52% | C: 50.48%, T: 49.52% | 1 |
| SAS | 489 | A: 52.76%, G: 47.24% | C: 52.76%, T: 47.24% | 1 |
| STU | 102 | A: 49.51%, G: 50.49% | C: 49.51%, T: 50.49% | 1 |
| TSI | 107 | A: 53.27%, G: 46.73% | C: 53.27%, T: 46.73% | 1 |
| YRI | 108 | A: 31.48%, G: 68.52% | C: 33.8%, T: 66.2% | 0.9 |

Abbreviations: ACB, African Caribbeans in Barbados; AFR, All African; ALL, All populations; AMR, Ad Mixed American; ASW, Americans of African Ancestry in SW USA; BEB, Bengali from Bangladesh; CDX, Chinese Dai in Xishuangbanna, China; CEU, Utah Residents (CEPH) with Northern and Western European Ancestry; CHB, Han Chinese in Beijing, China; CHS, Southern Han Chinese; CLM, Colombians from Medellin, Colombia; EAS, All East Asian; ESN, Esan in Nigeria; EUR, European; FIN, Finnish in Finland; GBR, British in England and Scotland; GIH, Gujarati Indian from Houston, Texas; GWD, Gambian in Western Divisions in the Gambia; IBS, Iberian Population in Spain; ITU, Indian Telugu from the UK; JPT, Japanese in Tokyo, Japan; KHV, Kinh in Ho Chi Minh City, Vietnam; LWK, Luhya in Webuye, Kenya; MSL, Mende in Sierra Leone; MXL, Mexican Ancestry from Los Angeles USA; PEL, Peruvians from Lima, Peru; PJL, Punjabi from Lahore, Pakistan; PUR, Puerto Ricans from Puerto Rico; SAS, South Asian; STU, Sri Lankan Tamil from the UK; TSI, Toscani in Italia; YRI, Yoruba in Ibadan, Nigeria.

**S2 Table.** Pairwise linkage disequilibrium (R^2^) for rs2569190 and rs3138078 CD14 SNPs.

| **Population** | **N** | **rs2569190 Allele Freq** | **rs3138078 Allele Freq** | **R^2^** |
| --- | --- | --- | --- | --- |
| ACB | 96 | A: 33.33%, G: 66.67% | C: 85.94%, A: 14.06% | 0.0818 |
| AFR | 661 | A: 30.11%, G: 69.89% | C: 80.03%, A: 19.97% | 0.1075 |
| ALL | 2504 | A: 46.88%, G: 53.12% | C: 75.7%, A: 24.3% | 0.2824 |
| AMR | 347 | A: 53.03%, G: 46.97% | C: 77.23%, A: 22.77% | 0.3249 |
| ASW | 61 | A: 25.41%, G: 74.59% | C: 78.69%, A: 21.31% | 0.0923 |
| BEB | 86 | A: 50.0%, G: 50.0% | C: 70.35%, A: 29.65% | 0.4215 |
| CDX | 93 | A: 54.3%, G: 45.7% | C: 65.59%, A: 34.41% | 0.6233 |
| CEU | 99 | A: 53.03%, G: 46.97% | C: 76.26%, A: 23.74% | 0.3514 |
| CHB | 103 | A: 61.65%, G: 38.35% | C: 73.3%, A: 26.7% | 0.5855 |
| CHS | 105 | A: 59.52%, G: 40.48% | C: 70.48%, A: 29.52% | 0.6161 |
| CLM | 94 | A: 44.15%, G: 55.85% | C: 78.19%, A: 21.81% | 0.2205 |
| EAS | 504 | A: 57.24%, G: 42.76% | C: 70.44%, A: 29.56% | 0.5619 |
| ESN | 99 | A: 23.74%, G: 76.26% | C: 83.33%, A: 16.67% | 0.0623 |
| EUR | 503 | A: 48.61%, G: 51.39% | C: 75.15%, A: 24.85% | 0.3128 |
| FIN | 99 | A: 38.38%, G: 61.62% | C: 72.73%, A: 27.27% | 0.2336 |
| GBR | 91 | A: 46.15%, G: 53.85% | C: 75.27%, A: 24.73% | 0.2815 |
| GIH | 103 | A: 58.25%, G: 41.75% | C: 80.1%, A: 19.9% | 0.3467 |
| GWD | 113 | A: 26.55%, G: 73.45% | C: 73.45%, A: 26.55% | 0.1306 |
| IBS | 107 | A: 51.4%, G: 48.6% | C: 77.1%, A: 22.9% | 0.3141 |
| ITU | 102 | A: 50.0%, G: 50.0% | C: 74.02%, A: 25.98% | 0.351 |
| JPT | 104 | A: 53.37%, G: 46.63% | C: 69.71%, A: 30.29% | 0.4972 |
| KHV | 99 | A: 57.07%, G: 42.93% | C: 72.73%, A: 27.27% | 0.4985 |
| LWK | 99 | A: 42.93%, G: 57.07% | C: 87.37%, A: 12.63% | 0.1087 |
| MSL | 85 | A: 25.29%, G: 74.71% | C: 74.71%, A: 25.29% | 0.1146 |
| MXL | 64 | A: 54.69%, G: 45.31% | C: 73.44%, A: 26.56% | 0.4365 |
| PEL | 85 | A: 64.71%, G: 35.29% | C: 82.35%, A: 17.65% | 0.3534 |
| PJL | 96 | A: 55.73%, G: 44.27% | C: 73.96%, A: 26.04% | 0.4432 |
| PUR | 104 | A: 50.48%, G: 49.52% | C: 74.52%, A: 25.48% | 0.3486 |
| SAS | 489 | A: 52.76%, G: 47.24% | C: 74.74%, A: 25.26% | 0.3774 |
| STU | 102 | A: 49.51%, G: 50.49% | C: 74.51%, A: 25.49% | 0.3355 |
| TSI | 107 | A: 53.27%, G: 46.73% | C: 74.3%, A: 25.7% | 0.3943 |
| YRI | 108 | A: 31.48%, G: 68.52% | C: 76.85%, A: 23.15% | 0.1384 |

Abbreviations: ACB, African Caribbeans in Barbados; AFR, All African; ALL, All populations; AMR, Ad Mixed American; ASW, Americans of African Ancestry in SW USA; BEB, Bengali from Bangladesh; CDX, Chinese Dai in Xishuangbanna, China; CEU, Utah Residents (CEPH) with Northern and Western European Ancestry; CHB, Han Chinese in Beijing, China; CHS, Southern Han Chinese; CLM, Colombians from Medellin, Colombia; EAS, All East Asian; ESN, Esan in Nigeria; EUR, European; FIN, Finnish in Finland; GBR, British in England and Scotland; GIH, Gujarati Indian from Houston, Texas; GWD, Gambian in Western Divisions in the Gambia; IBS, Iberian Population in Spain; ITU, Indian Telugu from the UK; JPT, Japanese in Tokyo, Japan; KHV, Kinh in Ho Chi Minh City, Vietnam; LWK, Luhya in Webuye, Kenya; MSL, Mende in Sierra Leone; MXL, Mexican Ancestry from Los Angeles USA; PEL, Peruvians from Lima, Peru; PJL, Punjabi from Lahore, Pakistan; PUR, Puerto Ricans from Puerto Rico; SAS, South Asian; STU, Sri Lankan Tamil from the UK; TSI, Toscani in Italia; YRI, Yoruba in Ibadan, Nigeria.

**S3 Table.** Pairwise linkage disequilibrium (R^2^ ) for rs2569190 and rs2915863 CD14 SNPs.

| **Population** | **N** | **rs2569190 Allele Freq** | **rs2915863 Allele Freq** | **R^2^** |
| --- | --- | --- | --- | --- |
| ACB | 96 | A: 33.33%, G: 66.67% | C: 23.44%, T: 76.56% | 0.6122 |
| AFR | 661 | A: 30.11%, G: 69.89% | C: 18.46%, T: 81.54% | 0.5193 |
| ALL | 2504 | A: 46.88%, G: 53.12% | C: 39.76%, T: 60.24% | 0.7434 |
| AMR | 347 | A: 53.03%, G: 46.97% | C: 44.81%, T: 55.19% | 0.7193 |
| ASW | 61 | A: 25.41%, G: 74.59% | C: 14.75%, T: 85.25% | 0.5081 |
| BEB | 86 | A: 50.0%, G: 50.0% | C: 47.09%, T: 52.91% | 0.8901 |
| CDX | 93 | A: 54.3%, G: 45.7% | C: 49.46%, T: 50.54% | 0.8237 |
| CEU | 99 | A: 53.03%, G: 46.97% | C: 44.95%, T: 55.05% | 0.7232 |
| CHB | 103 | A: 61.65%, G: 38.35% | C: 55.83%, T: 44.17% | 0.7861 |
| CHS | 105 | A: 59.52%, G: 40.48% | C: 56.67%, T: 43.33% | 0.8527 |
| CLM | 94 | A: 44.15%, G: 55.85% | C: 34.57%, T: 65.43% | 0.6685 |
| EAS | 504 | A: 57.24%, G: 42.76% | C: 53.57%, T: 46.43% | 0.847 |
| ESN | 99 | A: 23.74%, G: 76.26% | C: 14.14%, T: 85.86% | 0.5292 |
| EUR | 503 | A: 48.61%, G: 51.39% | C: 42.15%, T: 57.85% | 0.7702 |
| FIN | 99 | A: 38.38%, G: 61.62% | C: 29.29%, T: 70.71% | 0.665 |
| GBR | 91 | A: 46.15%, G: 53.85% | C: 42.31%, T: 57.69% | 0.8556 |
| GIH | 103 | A: 58.25%, G: 41.75% | C: 51.46%, T: 48.54% | 0.7597 |
| GWD | 113 | A: 26.55%, G: 73.45% | C: 17.7%, T: 82.3% | 0.595 |
| IBS | 107 | A: 51.4%, G: 48.6% | C: 47.66%, T: 52.34% | 0.861 |
| ITU | 102 | A: 50.0%, G: 50.0% | C: 45.59%, T: 54.41% | 0.8378 |
| JPT | 104 | A: 53.37%, G: 46.63% | C: 50.0%, T: 50.0% | 0.8739 |
| KHV | 99 | A: 57.07%, G: 42.93% | C: 55.56%, T: 44.44% | 0.9009 |
| LWK | 99 | A: 42.93%, G: 57.07% | C: 21.21%, T: 78.79% | 0.3579 |
| MSL | 85 | A: 25.29%, G: 74.71% | C: 18.82%, T: 81.18% | 0.6849 |
| MXL | 64 | A: 54.69%, G: 45.31% | C: 48.44%, T: 51.56% | 0.7784 |
| PEL | 85 | A: 64.71%, G: 35.29% | C: 59.41%, T: 40.59% | 0.7984 |
| PJL | 96 | A: 55.73%, G: 44.27% | C: 51.56%, T: 48.44% | 0.8456 |
| PUR | 104 | A: 50.48%, G: 49.52% | C: 39.9%, T: 60.1% | 0.6514 |
| SAS | 489 | A: 52.76%, G: 47.24% | C: 48.26%, T: 51.74% | 0.8352 |
| STU | 102 | A: 49.51%, G: 50.49% | C: 45.59%, T: 54.41% | 0.8544 |
| TSI | 107 | A: 53.27%, G: 46.73% | C: 45.79%, T: 54.21% | 0.7411 |
| YRI | 108 | A: 31.48%, G: 68.52% | C: 18.06%, T: 81.94% | 0.4443 |

Abbreviations: ACB, African Caribbeans in Barbados; AFR, All African; ALL, All populations; AMR, Ad Mixed American; ASW, Americans of African Ancestry in SW USA; BEB, Bengali from Bangladesh; CDX, Chinese Dai in Xishuangbanna, China; CEU, Utah Residents (CEPH) with Northern and Western European Ancestry; CHB, Han Chinese in Beijing, China; CHS, Southern Han Chinese; CLM, Colombians from Medellin, Colombia; EAS, All East Asian; ESN, Esan in Nigeria; EUR, European; FIN, Finnish in Finland; GBR, British in England and Scotland; GIH, Gujarati Indian from Houston, Texas; GWD, Gambian in Western Divisions in the Gambia; IBS, Iberian Population in Spain; ITU, Indian Telugu from the UK; JPT, Japanese in Tokyo, Japan; KHV, Kinh in Ho Chi Minh City, Vietnam; LWK, Luhya in Webuye, Kenya; MSL, Mende in Sierra Leone; MXL, Mexican Ancestry from Los Angeles USA; PEL, Peruvians from Lima, Peru; PJL, Punjabi from Lahore, Pakistan; PUR, Puerto Ricans from Puerto Rico; SAS, South Asian; STU, Sri Lankan Tamil from the UK; TSI, Toscani in Italia; YRI, Yoruba in Ibadan, Nigeria.

**S4 Table.** Pairwise linkage disequilibrium (R^2^ ) for rs2569190 and rs3138076 CD14 SNPs.

| **Population** | **N** | **rs2569190 Allele Freq** | **rs3138076 Allele Freq** | **R^2^** |
| --- | --- | --- | --- | --- |
| ACB | 96 | A: 33.33%, G: 66.67% | T: 85.94%, C: 14.06% | 0.0818 |
| AFR | 661 | A: 30.11%, G: 69.89% | T: 80.03%, C: 19.97% | 0.1075 |
| ALL | 2504 | A: 46.88%, G: 53.12% | T: 75.6%, C: 24.4% | 0.2839 |
| AMR | 347 | A: 53.03%, G: 46.97% | T: 77.23%, C: 22.77% | 0.3249 |
| ASW | 61 | A: 25.41%, G: 74.59% | T: 78.69%, C: 21.31% | 0.0923 |
| BEB | 86 | A: 50.0%, G: 50.0% | T: 70.35%, C: 29.65% | 0.4215 |
| CDX | 93 | A: 54.3%, G: 45.7% | T: 65.59%, C: 34.41% | 0.6233 |
| CEU | 99 | A: 53.03%, G: 46.97% | T: 75.76%, C: 24.24% | 0.3613 |
| CHB | 103 | A: 61.65%, G: 38.35% | T: 73.3%, C: 26.7% | 0.5855 |
| CHS | 105 | A: 59.52%, G: 40.48% | T: 70.48%, C: 29.52% | 0.6161 |
| CLM | 94 | A: 44.15%, G: 55.85% | T: 78.19%, C: 21.81% | 0.2205 |
| EAS | 504 | A: 57.24%, G: 42.76% | T: 70.44%, C: 29.56% | 0.5619 |
| ESN | 99 | A: 23.74%, G: 76.26% | T: 83.33%, C: 16.67% | 0.0623 |
| EUR | 503 | A: 48.61%, G: 51.39% | T: 74.65%, C: 25.35% | 0.3212 |
| FIN | 99 | A: 38.38%, G: 61.62% | T: 70.71%, C: 29.29% | 0.2581 |
| GBR | 91 | A: 46.15%, G: 53.85% | T: 75.27%, C: 24.73% | 0.2815 |
| GIH | 103 | A: 58.25%, G: 41.75% | T: 80.1%, C: 19.9% | 0.3467 |
| GWD | 113 | A: 26.55%, G: 73.45% | T: 73.45%, C: 26.55% | 0.1306 |
| IBS | 107 | A: 51.4%, G: 48.6% | T: 77.1%, C: 22.9% | 0.3141 |
| ITU | 102 | A: 50.0%, G: 50.0% | T: 74.02%, C: 25.98% | 0.351 |
| JPT | 104 | A: 53.37%, G: 46.63% | T: 69.71%, C: 30.29% | 0.4972 |
| KHV | 99 | A: 57.07%, G: 42.93% | T: 72.73%, C: 27.27% | 0.4985 |
| LWK | 99 | A: 42.93%, G: 57.07% | T: 87.37%, C: 12.63% | 0.1087 |
| MSL | 85 | A: 25.29%, G: 74.71% | T: 74.71%, C: 25.29% | 0.1146 |
| MXL | 64 | A: 54.69%, G: 45.31% | T: 73.44%, C: 26.56% | 0.4365 |
| PEL | 85 | A: 64.71%, G: 35.29% | T: 82.35%, C: 17.65% | 0.3534 |
| PJL | 96 | A: 55.73%, G: 44.27% | T: 73.96%, C: 26.04% | 0.4432 |
| PUR | 104 | A: 50.48%, G: 49.52% | T: 74.52%, C: 25.48% | 0.3486 |
| SAS | 489 | A: 52.76%, G: 47.24% | T: 74.74%, C: 25.26% | 0.3774 |
| STU | 102 | A: 49.51%, G: 50.49% | T: 74.51%, C: 25.49% | 0.3355 |
| TSI | 107 | A: 53.27%, G: 46.73% | T: 74.3%, C: 25.7% | 0.3943 |
| YRI | 108 | A: 31.48%, G: 68.52% | T: 76.85%, C: 23.15% | 0.1384 |

Abbreviations: ACB, African Caribbeans in Barbados; AFR, All African; ALL, All populations; AMR, Ad Mixed American; ASW, Americans of African Ancestry in SW USA; BEB, Bengali from Bangladesh; CDX, Chinese Dai in Xishuangbanna, China; CEU, Utah Residents (CEPH) with Northern and Western European Ancestry; CHB, Han Chinese in Beijing, China; CHS, Southern Han Chinese; CLM, Colombians from Medellin, Colombia; EAS, All East Asian; ESN, Esan in Nigeria; EUR, European; FIN, Finnish in Finland; GBR, British in England and Scotland; GIH, Gujarati Indian from Houston, Texas; GWD, Gambian in Western Divisions in the Gambia; IBS, Iberian Population in Spain; ITU, Indian Telugu from the UK; JPT, Japanese in Tokyo, Japan; KHV, Kinh in Ho Chi Minh City, Vietnam; LWK, Luhya in Webuye, Kenya; MSL, Mende in Sierra Leone; MXL, Mexican Ancestry from Los Angeles USA; PEL, Peruvians from Lima, Peru; PJL, Punjabi from Lahore, Pakistan; PUR, Puerto Ricans from Puerto Rico; SAS, South Asian; STU, Sri Lankan Tamil from the UK; TSI, Toscani in Italia; YRI, Yoruba in Ibadan, Nigeria.

**S5 Table.** Pairwise linkage disequilibrium (R^2^ ) for rs2569190 and rs5744455 CD14 SNPs.

| **Population** | **N** | **rs2569190 Allele Freq** | **rs5744455 Allele Freq** | **R^2^** |
| --- | --- | --- | --- | --- |
| ACB | 96 | A: 33.33%, G: 66.67% | G: 94.27%, A: 5.73% | 0.0304 |
| AFR | 661 | A: 30.11%, G: 69.89% | G: 94.02%, A: 5.98% | 0.0274 |
| ALL | 2504 | A: 46.88%, G: 53.12% | G: 79.85%, A: 20.15% | 0.2218 |
| AMR | 347 | A: 53.03%, G: 46.97% | G: 79.83%, A: 20.17% | 0.2776 |
| ASW | 61 | A: 25.41%, G: 74.59% | G: 90.98%, A: 9.02% | 0.0338 |
| BEB | 86 | A: 50.0%, G: 50.0% | G: 70.35%, A: 29.65% | 0.4215 |
| CDX | 93 | A: 54.3%, G: 45.7% | G: 65.59%, A: 34.41% | 0.6233 |
| CEU | 99 | A: 53.03%, G: 46.97% | G: 77.78%, A: 22.22% | 0.3226 |
| CHB | 103 | A: 61.65%, G: 38.35% | G: 73.3%, A: 26.7% | 0.5855 |
| CHS | 105 | A: 59.52%, G: 40.48% | G: 70.48%, A: 29.52% | 0.6161 |
| CLM | 94 | A: 44.15%, G: 55.85% | G: 82.45%, A: 17.55% | 0.1683 |
| EAS | 504 | A: 57.24%, G: 42.76% | G: 70.44%, A: 29.56% | 0.5619 |
| ESN | 99 | A: 23.74%, G: 76.26% | G: 93.94%, A: 6.06% | 0.0201 |
| EUR | 503 | A: 48.61%, G: 51.39% | G: 75.75%, A: 24.25% | 0.3029 |
| FIN | 99 | A: 38.38%, G: 61.62% | G: 72.73%, A: 27.27% | 0.2336 |
| GBR | 91 | A: 46.15%, G: 53.85% | G: 75.27%, A: 24.73% | 0.2815 |
| GIH | 103 | A: 58.25%, G: 41.75% | G: 79.61%, A: 20.39% | 0.3573 |
| GWD | 113 | A: 26.55%, G: 73.45% | G: 95.13%, A: 4.87% | 0.0185 |
| IBS | 107 | A: 51.4%, G: 48.6% | G: 77.57%, A: 22.43% | 0.3058 |
| ITU | 102 | A: 50.0%, G: 50.0% | G: 74.02%, A: 25.98% | 0.351 |
| JPT | 104 | A: 53.37%, G: 46.63% | G: 69.71%, A: 30.29% | 0.4972 |
| KHV | 99 | A: 57.07%, G: 42.93% | G: 72.73%, A: 27.27% | 0.4985 |
| LWK | 99 | A: 42.93%, G: 57.07% | G: 97.47%, A: 2.53% | 0.0195 |
| MSL | 85 | A: 25.29%, G: 74.71% | G: 92.94%, A: 7.06% | 0.0257 |
| MXL | 64 | A: 54.69%, G: 45.31% | G: 74.22%, A: 25.78% | 0.4192 |
| PEL | 85 | A: 64.71%, G: 35.29% | G: 83.53%, A: 16.47% | 0.3227 |
| PJL | 96 | A: 55.73%, G: 44.27% | G: 73.96%, A: 26.04% | 0.4432 |
| PUR | 104 | A: 50.48%, G: 49.52% | G: 77.88%, A: 22.12% | 0.2895 |
| SAS | 489 | A: 52.76%, G: 47.24% | G: 74.64%, A: 25.36% | 0.3794 |
| STU | 102 | A: 49.51%, G: 50.49% | G: 74.51%, A: 25.49% | 0.3355 |
| TSI | 107 | A: 53.27%, G: 46.73% | G: 75.23%, A: 24.77% | 0.3753 |
| YRI | 108 | A: 31.48%, G: 68.52% | G: 92.13%, A: 7.87% | 0.0393 |

Abbreviations: ACB, African Caribbeans in Barbados; AFR, All African; ALL, All populations; AMR, Ad Mixed American; ASW, Americans of African Ancestry in SW USA; BEB, Bengali from Bangladesh; CDX, Chinese Dai in Xishuangbanna, China; CEU, Utah Residents (CEPH) with Northern and Western European Ancestry; CHB, Han Chinese in Beijing, China; CHS, Southern Han Chinese; CLM, Colombians from Medellin, Colombia; EAS, All East Asian; ESN, Esan in Nigeria; EUR, European; FIN, Finnish in Finland; GBR, British in England and Scotland; GIH, Gujarati Indian from Houston, Texas; GWD, Gambian in Western Divisions in the Gambia; IBS, Iberian Population in Spain; ITU, Indian Telugu from the UK; JPT, Japanese in Tokyo, Japan; KHV, Kinh in Ho Chi Minh City, Vietnam; LWK, Luhya in Webuye, Kenya; MSL, Mende in Sierra Leone; MXL, Mexican Ancestry from Los Angeles USA; PEL, Peruvians from Lima, Peru; PJL, Punjabi from Lahore, Pakistan; PUR, Puerto Ricans from Puerto Rico; SAS, South Asian; STU, Sri Lankan Tamil from the UK; TSI, Toscani in Italia; YRI, Yoruba in Ibadan, Nigeria.

**S6 Table.** Pairwise linkage disequilibrium (R^2^ ) for rs2569190 and rs5744454 CD14 SNPs.

| **Population** | **N** | **rs2569190 Allele Freq** | **rs5744454 Allele Freq** | **R^2^** |
| --- | --- | --- | --- | --- |
| ACB | 96 | A: 33.33%, G: 66.67% | T: 85.94%, G: 14.06% | 0.0818 |
| AFR | 661 | A: 30.11%, G: 69.89% | T: 80.03%, G: 19.97% | 0.1075 |
| ALL | 2504 | A: 46.88%, G: 53.12% | T: 75.6%, G: 24.4% | 0.2839 |
| AMR | 347 | A: 53.03%, G: 46.97% | T: 77.23%, G: 22.77% | 0.3249 |
| ASW | 61 | A: 25.41%, G: 74.59% | T: 78.69%, G: 21.31% | 0.0923 |
| BEB | 86 | A: 50.0%, G: 50.0% | T: 70.35%, G: 29.65% | 0.4215 |
| CDX | 93 | A: 54.3%, G: 45.7% | T: 65.59%, G: 34.41% | 0.6233 |
| CEU | 99 | A: 53.03%, G: 46.97% | T: 75.76%, G: 24.24% | 0.3613 |
| CHB | 103 | A: 61.65%, G: 38.35% | T: 73.3%, G: 26.7% | 0.5855 |
| CHS | 105 | A: 59.52%, G: 40.48% | T: 70.48%, G: 29.52% | 0.6161 |
| CLM | 94 | A: 44.15%, G: 55.85% | T: 78.19%, G: 21.81% | 0.2205 |
| EAS | 504 | A: 57.24%, G: 42.76% | T: 70.44%, G: 29.56% | 0.5619 |
| ESN | 99 | A: 23.74%, G: 76.26% | T: 83.33%, G: 16.67% | 0.0623 |
| EUR | 503 | A: 48.61%, G: 51.39% | T: 74.65%, G: 25.35% | 0.3212 |
| FIN | 99 | A: 38.38%, G: 61.62% | T: 70.71%, G: 29.29% | 0.2581 |
| GBR | 91 | A: 46.15%, G: 53.85% | T: 75.27%, G: 24.73% | 0.2815 |
| GIH | 103 | A: 58.25%, G: 41.75% | T: 80.1%, G: 19.9% | 0.3467 |
| GWD | 113 | A: 26.55%, G: 73.45% | T: 73.45%, G: 26.55% | 0.1306 |
| IBS | 107 | A: 51.4%, G: 48.6% | T: 77.1%, G: 22.9% | 0.3141 |
| ITU | 102 | A: 50.0%, G: 50.0% | T: 74.02%, G: 25.98% | 0.351 |
| JPT | 104 | A: 53.37%, G: 46.63% | T: 69.71%, G: 30.29% | 0.4972 |
| KHV | 99 | A: 57.07%, G: 42.93% | T: 72.73%, G: 27.27% | 0.4985 |
| LWK | 99 | A: 42.93%, G: 57.07% | T: 87.37%, G: 12.63% | 0.1087 |
| MSL | 85 | A: 25.29%, G: 74.71% | T: 74.71%, G: 25.29% | 0.1146 |
| MXL | 64 | A: 54.69%, G: 45.31% | T: 73.44%, G: 26.56% | 0.4365 |
| PEL | 85 | A: 64.71%, G: 35.29% | T: 82.35%, G: 17.65% | 0.3534 |
| PJL | 96 | A: 55.73%, G: 44.27% | T: 73.96%, G: 26.04% | 0.4432 |
| PUR | 104 | A: 50.48%, G: 49.52% | T: 74.52%, G: 25.48% | 0.3486 |
| SAS | 489 | A: 52.76%, G: 47.24% | T: 74.74%, G: 25.26% | 0.3774 |
| STU | 102 | A: 49.51%, G: 50.49% | T: 74.51%, G: 25.49% | 0.3355 |
| TSI | 107 | A: 53.27%, G: 46.73% | T: 74.3%, G: 25.7% | 0.3943 |
| YRI | 108 | A: 31.48%, G: 68.52% | T: 76.85%, G: 23.15% | 0.1384 |

Abbreviations: ACB, African Caribbeans in Barbados; AFR, All African; ALL, All populations; AMR, Ad Mixed American; ASW, Americans of African Ancestry in SW USA; BEB, Bengali from Bangladesh; CDX, Chinese Dai in Xishuangbanna, China; CEU, Utah Residents (CEPH) with Northern and Western European Ancestry; CHB, Han Chinese in Beijing, China; CHS, Southern Han Chinese; CLM, Colombians from Medellin, Colombia; EAS, All East Asian; ESN, Esan in Nigeria; EUR, European; FIN, Finnish in Finland; GBR, British in England and Scotland; GIH, Gujarati Indian from Houston, Texas; GWD, Gambian in Western Divisions in the Gambia; IBS, Iberian Population in Spain; ITU, Indian Telugu from the UK; JPT, Japanese in Tokyo, Japan; KHV, Kinh in Ho Chi Minh City, Vietnam; LWK, Luhya in Webuye, Kenya; MSL, Mende in Sierra Leone; MXL, Mexican Ancestry from Los Angeles USA; PEL, Peruvians from Lima, Peru; PJL, Punjabi from Lahore, Pakistan; PUR, Puerto Ricans from Puerto Rico; SAS, South Asian; STU, Sri Lankan Tamil from the UK; TSI, Toscani in Italia; YRI, Yoruba in Ibadan, Nigeria.

**S7 Table.** Pairwise linkage disequilibrium (R^2^ ) for rs2569191 and rs3138078 CD14 SNPs.

| **Population** | **N** | **rs2569191 Allele Freq** | **rs3138078 Allele Freq** | **R^2^** |
| --- | --- | --- | --- | --- |
| ACB | 96 | C: 36.46%, T: 63.54% | C: 85.94%, A: 14.06% | 0.0939 |
| AFR | 661 | C: 32.3%, T: 67.7% | C: 80.03%, A: 19.97% | 0.119 |
| ALL | 2504 | C: 47.48%, T: 52.52% | C: 75.7%, A: 24.3% | 0.2903 |
| AMR | 347 | C: 52.88%, T: 47.12% | C: 77.23%, A: 22.77% | 0.3308 |
| ASW | 61 | C: 26.23%, T: 73.77% | C: 78.69%, A: 21.31% | 0.0963 |
| BEB | 86 | C: 50.0%, T: 50.0% | C: 70.35%, A: 29.65% | 0.4215 |
| CDX | 93 | C: 54.3%, T: 45.7% | C: 65.59%, A: 34.41% | 0.6233 |
| CEU | 99 | C: 53.03%, T: 46.97% | C: 76.26%, A: 23.74% | 0.3514 |
| CHB | 103 | C: 61.65%, T: 38.35% | C: 73.3%, A: 26.7% | 0.5855 |
| CHS | 105 | C: 60.0%, T: 40.0% | C: 70.48%, A: 29.52% | 0.6284 |
| CLM | 94 | C: 43.62%, T: 56.38% | C: 78.19%, A: 21.81% | 0.2158 |
| EAS | 504 | C: 57.44%, T: 42.56% | C: 70.44%, A: 29.56% | 0.5665 |
| ESN | 99 | C: 25.25%, T: 74.75% | C: 83.33%, A: 16.67% | 0.0676 |
| EUR | 503 | C: 48.61%, T: 51.39% | C: 75.15%, A: 24.85% | 0.3128 |
| FIN | 99 | C: 38.38%, T: 61.62% | C: 72.73%, A: 27.27% | 0.2336 |
| GBR | 91 | C: 46.15%, T: 53.85% | C: 75.27%, A: 24.73% | 0.2815 |
| GIH | 103 | C: 58.25%, T: 41.75% | C: 80.1%, A: 19.9% | 0.3467 |
| GWD | 113 | C: 29.65%, T: 70.35% | C: 73.45%, A: 26.55% | 0.1523 |
| IBS | 107 | C: 51.4%, T: 48.6% | C: 77.1%, A: 22.9% | 0.3141 |
| ITU | 102 | C: 50.0%, T: 50.0% | C: 74.02%, A: 25.98% | 0.351 |
| JPT | 104 | C: 53.37%, T: 46.63% | C: 69.71%, A: 30.29% | 0.4972 |
| KHV | 99 | C: 57.58%, T: 42.42% | C: 72.73%, A: 27.27% | 0.5089 |
| LWK | 99 | C: 43.43%, T: 56.57% | C: 87.37%, A: 12.63% | 0.111 |
| MSL | 85 | C: 28.82%, T: 71.18% | C: 74.71%, A: 25.29% | 0.1371 |
| MXL | 64 | C: 55.47%, T: 44.53% | C: 73.44%, A: 26.56% | 0.4505 |
| PEL | 85 | C: 64.12%, T: 35.88% | C: 82.35%, A: 17.65% | 0.3829 |
| PJL | 96 | C: 55.73%, T: 44.27% | C: 73.96%, A: 26.04% | 0.4432 |
| PUR | 104 | C: 50.48%, T: 49.52% | C: 74.52%, A: 25.48% | 0.3486 |
| SAS | 489 | C: 52.76%, T: 47.24% | C: 74.74%, A: 25.26% | 0.3774 |
| STU | 102 | C: 49.51%, T: 50.49% | C: 74.51%, A: 25.49% | 0.3355 |
| TSI | 107 | C: 53.27%, T: 46.73% | C: 74.3%, A: 25.7% | 0.3943 |
| YRI | 108 | C: 33.8%, T: 66.2% | C: 76.85%, A: 23.15% | 0.1538 |

Abbreviations: ACB, African Caribbeans in Barbados; AFR, All African; ALL, All populations; AMR, Ad Mixed American; ASW, Americans of African Ancestry in SW USA; BEB, Bengali from Bangladesh; CDX, Chinese Dai in Xishuangbanna, China; CEU, Utah Residents (CEPH) with Northern and Western European Ancestry; CHB, Han Chinese in Beijing, China; CHS, Southern Han Chinese; CLM, Colombians from Medellin, Colombia; EAS, All East Asian; ESN, Esan in Nigeria; EUR, European; FIN, Finnish in Finland; GBR, British in England and Scotland; GIH, Gujarati Indian from Houston, Texas; GWD, Gambian in Western Divisions in the Gambia; IBS, Iberian Population in Spain; ITU, Indian Telugu from the UK; JPT, Japanese in Tokyo, Japan; KHV, Kinh in Ho Chi Minh City, Vietnam; LWK, Luhya in Webuye, Kenya; MSL, Mende in Sierra Leone; MXL, Mexican Ancestry from Los Angeles USA; PEL, Peruvians from Lima, Peru; PJL, Punjabi from Lahore, Pakistan; PUR, Puerto Ricans from Puerto Rico; SAS, South Asian; STU, Sri Lankan Tamil from the UK; TSI, Toscani in Italia; YRI, Yoruba in Ibadan, Nigeria.

**S8 Table.** Pairwise linkage disequilibrium (R^2^ ) for rs2569191 and rs2915863 CD14 SNPs.

| **Population** | **N** | **rs2569191 Allele Freq** | **rs2915863 Allele Freq** | **R^2^** |
| --- | --- | --- | --- | --- |
| ACB | 96 | C: 36.46%, T: 63.54% | C: 23.44%, T: 76.56% | 0.5335 |
| AFR | 661 | C: 32.3%, T: 67.7% | C: 18.46%, T: 81.54% | 0.4744 |
| ALL | 2504 | C: 47.48%, T: 52.52% | C: 39.76%, T: 60.24% | 0.7299 |
| AMR | 347 | C: 52.88%, T: 47.12% | C: 44.81%, T: 55.19% | 0.7235 |
| ASW | 61 | C: 26.23%, T: 73.77% | C: 14.75%, T: 85.25% | 0.4868 |
| BEB | 86 | C: 50.0%, T: 50.0% | C: 47.09%, T: 52.91% | 0.8901 |
| CDX | 93 | C: 54.3%, T: 45.7% | C: 49.46%, T: 50.54% | 0.8237 |
| CEU | 99 | C: 53.03%, T: 46.97% | C: 44.95%, T: 55.05% | 0.7232 |
| CHB | 103 | C: 61.65%, T: 38.35% | C: 55.83%, T: 44.17% | 0.7861 |
| CHS | 105 | C: 60.0%, T: 40.0% | C: 56.67%, T: 43.33% | 0.8718 |
| CLM | 94 | C: 43.62%, T: 56.38% | C: 34.57%, T: 65.43% | 0.6831 |
| EAS | 504 | C: 57.44%, T: 42.56% | C: 53.57%, T: 46.43% | 0.8549 |
| ESN | 99 | C: 25.25%, T: 74.75% | C: 14.14%, T: 85.86% | 0.4875 |
| EUR | 503 | C: 48.61%, T: 51.39% | C: 42.15%, T: 57.85% | 0.7702 |
| FIN | 99 | C: 38.38%, T: 61.62% | C: 29.29%, T: 70.71% | 0.665 |
| GBR | 91 | C: 46.15%, T: 53.85% | C: 42.31%, T: 57.69% | 0.8556 |
| GIH | 103 | C: 58.25%, T: 41.75% | C: 51.46%, T: 48.54% | 0.7597 |
| GWD | 113 | C: 29.65%, T: 70.35% | C: 17.7%, T: 82.3% | 0.5104 |
| IBS | 107 | C: 51.4%, T: 48.6% | C: 47.66%, T: 52.34% | 0.861 |
| ITU | 102 | C: 50.0%, T: 50.0% | C: 45.59%, T: 54.41% | 0.8378 |
| JPT | 104 | C: 53.37%, T: 46.63% | C: 50.0%, T: 50.0% | 0.8739 |
| KHV | 99 | C: 57.58%, T: 42.42% | C: 55.56%, T: 44.44% | 0.9211 |
| LWK | 99 | C: 43.43%, T: 56.57% | C: 21.21%, T: 78.79% | 0.3506 |
| MSL | 85 | C: 28.82%, T: 71.18% | C: 18.82%, T: 81.18% | 0.5726 |
| MXL | 64 | C: 55.47%, T: 44.53% | C: 48.44%, T: 51.56% | 0.7542 |
| PEL | 85 | C: 64.12%, T: 35.88% | C: 59.41%, T: 40.59% | 0.8192 |
| PJL | 96 | C: 55.73%, T: 44.27% | C: 51.56%, T: 48.44% | 0.8456 |
| PUR | 104 | C: 50.48%, T: 49.52% | C: 39.9%, T: 60.1% | 0.6514 |
| SAS | 489 | C: 52.76%, T: 47.24% | C: 48.26%, T: 51.74% | 0.8352 |
| STU | 102 | C: 49.51%, T: 50.49% | C: 45.59%, T: 54.41% | 0.8544 |
| TSI | 107 | C: 53.27%, T: 46.73% | C: 45.79%, T: 54.21% | 0.7411 |
| YRI | 108 | C: 33.8%, T: 66.2% | C: 18.06%, T: 81.94% | 0.4316 |

Abbreviations: ACB, African Caribbeans in Barbados; AFR, All African; ALL, All populations; AMR, Ad Mixed American; ASW, Americans of African Ancestry in SW USA; BEB, Bengali from Bangladesh; CDX, Chinese Dai in Xishuangbanna, China; CEU, Utah Residents (CEPH) with Northern and Western European Ancestry; CHB, Han Chinese in Beijing, China; CHS, Southern Han Chinese; CLM, Colombians from Medellin, Colombia; EAS, All East Asian; ESN, Esan in Nigeria; EUR, European; FIN, Finnish in Finland; GBR, British in England and Scotland; GIH, Gujarati Indian from Houston, Texas; GWD, Gambian in Western Divisions in the Gambia; IBS, Iberian Population in Spain; ITU, Indian Telugu from the UK; JPT, Japanese in Tokyo, Japan; KHV, Kinh in Ho Chi Minh City, Vietnam; LWK, Luhya in Webuye, Kenya; MSL, Mende in Sierra Leone; MXL, Mexican Ancestry from Los Angeles USA; PEL, Peruvians from Lima, Peru; PJL, Punjabi from Lahore, Pakistan; PUR, Puerto Ricans from Puerto Rico; SAS, South Asian; STU, Sri Lankan Tamil from the UK; TSI, Toscani in Italia; YRI, Yoruba in Ibadan, Nigeria.

**S9 Table.** Pairwise linkage disequilibrium (R^2^ ) for rs2569191 and rs3138076 CD14 SNPs.

| **Population** | **N** | **rs2569191 Allele Freq** | **rs3138076 Allele Freq** | **R^2^** |
| --- | --- | --- | --- | --- |
| ACB | 96 | C: 36.46%, T: 63.54% | T: 85.94%, C: 14.06% | 0.0939 |
| AFR | 661 | C: 32.3%, T: 67.7% | T: 80.03%, C: 19.97% | 0.119 |
| ALL | 2504 | C: 47.48%, T: 52.52% | T: 75.6%, C: 24.4% | 0.2918 |
| AMR | 347 | C: 52.88%, T: 47.12% | T: 77.23%, C: 22.77% | 0.3308 |
| ASW | 61 | C: 26.23%, T: 73.77% | T: 78.69%, C: 21.31% | 0.0963 |
| BEB | 86 | C: 50.0%, T: 50.0% | T: 70.35%, C: 29.65% | 0.4215 |
| CDX | 93 | C: 54.3%, T: 45.7% | T: 65.59%, C: 34.41% | 0.6233 |
| CEU | 99 | C: 53.03%, T: 46.97% | T: 75.76%, C: 24.24% | 0.3613 |
| CHB | 103 | C: 61.65%, T: 38.35% | T: 73.3%, C: 26.7% | 0.5855 |
| CHS | 105 | C: 60.0%, T: 40.0% | T: 70.48%, C: 29.52% | 0.6284 |
| CLM | 94 | C: 43.62%, T: 56.38% | T: 78.19%, C: 21.81% | 0.2158 |
| EAS | 504 | C: 57.44%, T: 42.56% | T: 70.44%, C: 29.56% | 0.5665 |
| ESN | 99 | C: 25.25%, T: 74.75% | T: 83.33%, C: 16.67% | 0.0676 |
| EUR | 503 | C: 48.61%, T: 51.39% | T: 74.65%, C: 25.35% | 0.3212 |
| FIN | 99 | C: 38.38%, T: 61.62% | T: 70.71%, C: 29.29% | 0.2581 |
| GBR | 91 | C: 46.15%, T: 53.85% | T: 75.27%, C: 24.73% | 0.2815 |
| GIH | 103 | C: 58.25%, T: 41.75% | T: 80.1%, C: 19.9% | 0.3467 |
| GWD | 113 | C: 29.65%, T: 70.35% | T: 73.45%, C: 26.55% | 0.1523 |
| IBS | 107 | C: 51.4%, T: 48.6% | T: 77.1%, C: 22.9% | 0.3141 |
| ITU | 102 | C: 50.0%, T: 50.0% | T: 74.02%, C: 25.98% | 0.351 |
| JPT | 104 | C: 53.37%, T: 46.63% | T: 69.71%, C: 30.29% | 0.4972 |
| KHV | 99 | C: 57.58%, T: 42.42% | T: 72.73%, C: 27.27% | 0.5089 |
| LWK | 99 | C: 43.43%, T: 56.57% | T: 87.37%, C: 12.63% | 0.111 |
| MSL | 85 | C: 28.82%, T: 71.18% | T: 74.71%, C: 25.29% | 0.1371 |
| MXL | 64 | C: 55.47%, T: 44.53% | T: 73.44%, C: 26.56% | 0.4505 |
| PEL | 85 | C: 64.12%, T: 35.88% | T: 82.35%, C: 17.65% | 0.3829 |
| PJL | 96 | C: 55.73%, T: 44.27% | T: 73.96%, C: 26.04% | 0.4432 |
| PUR | 104 | C: 50.48%, T: 49.52% | T: 74.52%, C: 25.48% | 0.3486 |
| SAS | 489 | C: 52.76%, T: 47.24% | T: 74.74%, C: 25.26% | 0.3774 |
| STU | 102 | C: 49.51%, T: 50.49% | T: 74.51%, C: 25.49% | 0.3355 |
| TSI | 107 | C: 53.27%, T: 46.73% | T: 74.3%, C: 25.7% | 0.3943 |
| YRI | 108 | C: 33.8%, T: 66.2% | T: 76.85%, C: 23.15% | 0.1538 |

Abbreviations: ACB, African Caribbeans in Barbados; AFR, All African; ALL, All populations; AMR, Ad Mixed American; ASW, Americans of African Ancestry in SW USA; BEB, Bengali from Bangladesh; CDX, Chinese Dai in Xishuangbanna, China; CEU, Utah Residents (CEPH) with Northern and Western European Ancestry; CHB, Han Chinese in Beijing, China; CHS, Southern Han Chinese; CLM, Colombians from Medellin, Colombia; EAS, All East Asian; ESN, Esan in Nigeria; EUR, European; FIN, Finnish in Finland; GBR, British in England and Scotland; GIH, Gujarati Indian from Houston, Texas; GWD, Gambian in Western Divisions in the Gambia; IBS, Iberian Population in Spain; ITU, Indian Telugu from the UK; JPT, Japanese in Tokyo, Japan; KHV, Kinh in Ho Chi Minh City, Vietnam; LWK, Luhya in Webuye, Kenya; MSL, Mende in Sierra Leone; MXL, Mexican Ancestry from Los Angeles USA; PEL, Peruvians from Lima, Peru; PJL, Punjabi from Lahore, Pakistan; PUR, Puerto Ricans from Puerto Rico; SAS, South Asian; STU, Sri Lankan Tamil from the UK; TSI, Toscani in Italia; YRI, Yoruba in Ibadan, Nigeria.

**S10 Table.** Pairwise linkage disequilibrium (R^2^ ) for rs2569191 and rs5744455 CD14 SNPs.

| **Population** | **N** | **rs2569191 Allele Freq** | **rs5744455 Allele Freq** | **R^2^** |
| --- | --- | --- | --- | --- |
| ACB | 96 | C: 36.46%, T: 63.54% | G: 94.27%, A: 5.73% | 0.0349 |
| AFR | 661 | C: 32.3%, T: 67.7% | G: 94.02%, A: 5.98% | 0.0303 |
| ALL | 2504 | C: 47.48%, T: 52.52% | G: 79.85%, A: 20.15% | 0.2272 |
| AMR | 347 | C: 52.88%, T: 47.12% | G: 79.83%, A: 20.17% | 0.276 |
| ASW | 61 | C: 26.23%, T: 73.77% | G: 90.98%, A: 9.02% | 0.0352 |
| BEB | 86 | C: 50.0%, T: 50.0% | G: 70.35%, A: 29.65% | 0.4215 |
| CDX | 93 | C: 54.3%, T: 45.7% | G: 65.59%, A: 34.41% | 0.6233 |
| CEU | 99 | C: 53.03%, T: 46.97% | G: 77.78%, A: 22.22% | 0.3226 |
| CHB | 103 | C: 61.65%, T: 38.35% | G: 73.3%, A: 26.7% | 0.5855 |
| CHS | 105 | C: 60.0%, T: 40.0% | G: 70.48%, A: 29.52% | 0.6284 |
| CLM | 94 | C: 43.62%, T: 56.38% | G: 82.45%, A: 17.55% | 0.1647 |
| EAS | 504 | C: 57.44%, T: 42.56% | G: 70.44%, A: 29.56% | 0.5665 |
| ESN | 99 | C: 25.25%, T: 74.75% | G: 93.94%, A: 6.06% | 0.0218 |
| EUR | 503 | C: 48.61%, T: 51.39% | G: 75.75%, A: 24.25% | 0.3029 |
| FIN | 99 | C: 38.38%, T: 61.62% | G: 72.73%, A: 27.27% | 0.2336 |
| GBR | 91 | C: 46.15%, T: 53.85% | G: 75.27%, A: 24.73% | 0.2815 |
| GIH | 103 | C: 58.25%, T: 41.75% | G: 79.61%, A: 20.39% | 0.3573 |
| GWD | 113 | C: 29.65%, T: 70.35% | G: 95.13%, A: 4.87% | 0.0216 |
| IBS | 107 | C: 51.4%, T: 48.6% | G: 77.57%, A: 22.43% | 0.3058 |
| ITU | 102 | C: 50.0%, T: 50.0% | G: 74.02%, A: 25.98% | 0.351 |
| JPT | 104 | C: 53.37%, T: 46.63% | G: 69.71%, A: 30.29% | 0.4972 |
| KHV | 99 | C: 57.58%, T: 42.42% | G: 72.73%, A: 27.27% | 0.5089 |
| LWK | 99 | C: 43.43%, T: 56.57% | G: 97.47%, A: 2.53% | 0.0199 |
| MSL | 85 | C: 28.82%, T: 71.18% | G: 92.94%, A: 7.06% | 0.0308 |
| MXL | 64 | C: 55.47%, T: 44.53% | G: 74.22%, A: 25.78% | 0.3867 |
| PEL | 85 | C: 64.12%, T: 35.88% | G: 83.53%, A: 16.47% | 0.3523 |
| PJL | 96 | C: 55.73%, T: 44.27% | G: 73.96%, A: 26.04% | 0.4432 |
| PUR | 104 | C: 50.48%, T: 49.52% | G: 77.88%, A: 22.12% | 0.2895 |
| SAS | 489 | C: 52.76%, T: 47.24% | G: 74.64%, A: 25.36% | 0.3794 |
| STU | 102 | C: 49.51%, T: 50.49% | G: 74.51%, A: 25.49% | 0.3355 |
| TSI | 107 | C: 53.27%, T: 46.73% | G: 75.23%, A: 24.77% | 0.3753 |
| YRI | 108 | C: 33.8%, T: 66.2% | G: 92.13%, A: 7.87% | 0.0436 |

Abbreviations: ACB, African Caribbeans in Barbados; AFR, All African; ALL, All populations; AMR, Ad Mixed American; ASW, Americans of African Ancestry in SW USA; BEB, Bengali from Bangladesh; CDX, Chinese Dai in Xishuangbanna, China; CEU, Utah Residents (CEPH) with Northern and Western European Ancestry; CHB, Han Chinese in Beijing, China; CHS, Southern Han Chinese; CLM, Colombians from Medellin, Colombia; EAS, All East Asian; ESN, Esan in Nigeria; EUR, European; FIN, Finnish in Finland; GBR, British in England and Scotland; GIH, Gujarati Indian from Houston, Texas; GWD, Gambian in Western Divisions in the Gambia; IBS, Iberian Population in Spain; ITU, Indian Telugu from the UK; JPT, Japanese in Tokyo, Japan; KHV, Kinh in Ho Chi Minh City, Vietnam; LWK, Luhya in Webuye, Kenya; MSL, Mende in Sierra Leone; MXL, Mexican Ancestry from Los Angeles USA; PEL, Peruvians from Lima, Peru; PJL, Punjabi from Lahore, Pakistan; PUR, Puerto Ricans from Puerto Rico; SAS, South Asian; STU, Sri Lankan Tamil from the UK; TSI, Toscani in Italia; YRI, Yoruba in Ibadan, Nigeria.

**S11 Table.** Pairwise linkage disequilibrium (R^2^ ) for rs2569191 and rs5744454 CD14 SNPs.

| **Population** | **N** | **rs2569191 Allele Freq** | **rs5744454 Allele Freq** | **R^2^** |
| --- | --- | --- | --- | --- |
| ACB | 96 | C: 36.46%, T: 63.54% | T: 85.94%, G: 14.06% | 0.0939 |
| AFR | 661 | C: 32.3%, T: 67.7% | T: 80.03%, G: 19.97% | 0.119 |
| ALL | 2504 | C: 47.48%, T: 52.52% | T: 75.6%, G: 24.4% | 0.2918 |
| AMR | 347 | C: 52.88%, T: 47.12% | T: 77.23%, G: 22.77% | 0.3308 |
| ASW | 61 | C: 26.23%, T: 73.77% | T: 78.69%, G: 21.31% | 0.0963 |
| BEB | 86 | C: 50.0%, T: 50.0% | T: 70.35%, G: 29.65% | 0.4215 |
| CDX | 93 | C: 54.3%, T: 45.7% | T: 65.59%, G: 34.41% | 0.6233 |
| CEU | 99 | C: 53.03%, T: 46.97% | T: 75.76%, G: 24.24% | 0.3613 |
| CHB | 103 | C: 61.65%, T: 38.35% | T: 73.3%, G: 26.7% | 0.5855 |
| CHS | 105 | C: 60.0%, T: 40.0% | T: 70.48%, G: 29.52% | 0.6284 |
| CLM | 94 | C: 43.62%, T: 56.38% | T: 78.19%, G: 21.81% | 0.2158 |
| EAS | 504 | C: 57.44%, T: 42.56% | T: 70.44%, G: 29.56% | 0.5665 |
| ESN | 99 | C: 25.25%, T: 74.75% | T: 83.33%, G: 16.67% | 0.0676 |
| EUR | 503 | C: 48.61%, T: 51.39% | T: 74.65%, G: 25.35% | 0.3212 |
| FIN | 99 | C: 38.38%, T: 61.62% | T: 70.71%, G: 29.29% | 0.2581 |
| GBR | 91 | C: 46.15%, T: 53.85% | T: 75.27%, G: 24.73% | 0.2815 |
| GIH | 103 | C: 58.25%, T: 41.75% | T: 80.1%, G: 19.9% | 0.3467 |
| GWD | 113 | C: 29.65%, T: 70.35% | T: 73.45%, G: 26.55% | 0.1523 |
| IBS | 107 | C: 51.4%, T: 48.6% | T: 77.1%, G: 22.9% | 0.3141 |
| ITU | 102 | C: 50.0%, T: 50.0% | T: 74.02%, G: 25.98% | 0.351 |
| JPT | 104 | C: 53.37%, T: 46.63% | T: 69.71%, G: 30.29% | 0.4972 |
| KHV | 99 | C: 57.58%, T: 42.42% | T: 72.73%, G: 27.27% | 0.5089 |
| LWK | 99 | C: 43.43%, T: 56.57% | T: 87.37%, G: 12.63% | 0.111 |
| MSL | 85 | C: 28.82%, T: 71.18% | T: 74.71%, G: 25.29% | 0.1371 |
| MXL | 64 | C: 55.47%, T: 44.53% | T: 73.44%, G: 26.56% | 0.4505 |
| PEL | 85 | C: 64.12%, T: 35.88% | T: 82.35%, G: 17.65% | 0.3829 |
| PJL | 96 | C: 55.73%, T: 44.27% | T: 73.96%, G: 26.04% | 0.4432 |
| PUR | 104 | C: 50.48%, T: 49.52% | T: 74.52%, G: 25.48% | 0.3486 |
| SAS | 489 | C: 52.76%, T: 47.24% | T: 74.74%, G: 25.26% | 0.3774 |
| STU | 102 | C: 49.51%, T: 50.49% | T: 74.51%, G: 25.49% | 0.3355 |
| TSI | 107 | C: 53.27%, T: 46.73% | T: 74.3%, G: 25.7% | 0.3943 |
| YRI | 108 | C: 33.8%, T: 66.2% | T: 76.85%, G: 23.15% | 0.1538 |

Abbreviations: ACB, African Caribbeans in Barbados; AFR, All African; ALL, All populations; AMR, Ad Mixed American; ASW, Americans of African Ancestry in SW USA; BEB, Bengali from Bangladesh; CDX, Chinese Dai in Xishuangbanna, China; CEU, Utah Residents (CEPH) with Northern and Western European Ancestry; CHB, Han Chinese in Beijing, China; CHS, Southern Han Chinese; CLM, Colombians from Medellin, Colombia; EAS, All East Asian; ESN, Esan in Nigeria; EUR, European; FIN, Finnish in Finland; GBR, British in England and Scotland; GIH, Gujarati Indian from Houston, Texas; GWD, Gambian in Western Divisions in the Gambia; IBS, Iberian Population in Spain; ITU, Indian Telugu from the UK; JPT, Japanese in Tokyo, Japan; KHV, Kinh in Ho Chi Minh City, Vietnam; LWK, Luhya in Webuye, Kenya; MSL, Mende in Sierra Leone; MXL, Mexican Ancestry from Los Angeles USA; PEL, Peruvians from Lima, Peru; PJL, Punjabi from Lahore, Pakistan; PUR, Puerto Ricans from Puerto Rico; SAS, South Asian; STU, Sri Lankan Tamil from the UK; TSI, Toscani in Italia; YRI, Yoruba in Ibadan, Nigeria.

**S12 Table.** Pairwise linkage disequilibrium (R^2^) for rs3138078 and rs2915863 CD14 SNPs.

| **Population** | **N** | **rs3138078 Allele Freq** | **rs2915863 Allele Freq** | **R^2^** |
| --- | --- | --- | --- | --- |
| ACB | 96 | C: 85.94%, A: 14.06% | C: 23.44%, T: 76.56% | 0.0501 |
| AFR | 661 | C: 80.03%, A: 19.97% | C: 18.46%, T: 81.54% | 0.0565 |
| ALL | 2504 | C: 75.7%, A: 24.3% | C: 39.76%, T: 60.24% | 0.2119 |
| AMR | 347 | C: 77.23%, A: 22.77% | C: 44.81%, T: 55.19% | 0.2394 |
| ASW | 61 | C: 78.69%, A: 21.31% | C: 14.75%, T: 85.25% | 0.0469 |
| BEB | 86 | C: 70.35%, A: 29.65% | C: 47.09%, T: 52.91% | 0.3752 |
| CDX | 93 | C: 65.59%, A: 34.41% | C: 49.46%, T: 50.54% | 0.5134 |
| CEU | 99 | C: 76.26%, A: 23.74% | C: 44.95%, T: 55.05% | 0.2541 |
| CHB | 103 | C: 73.3%, A: 26.7% | C: 55.83%, T: 44.17% | 0.4603 |
| CHS | 105 | C: 70.48%, A: 29.52% | C: 56.67%, T: 43.33% | 0.5478 |
| CLM | 94 | C: 78.19%, A: 21.81% | C: 34.57%, T: 65.43% | 0.1474 |
| EAS | 504 | C: 70.44%, A: 29.56% | C: 53.57%, T: 46.43% | 0.4843 |
| ESN | 99 | C: 83.33%, A: 16.67% | C: 14.14%, T: 85.86% | 0.0329 |
| EUR | 503 | C: 75.15%, A: 24.85% | C: 42.15%, T: 57.85% | 0.2409 |
| FIN | 99 | C: 72.73%, A: 27.27% | C: 29.29%, T: 70.71% | 0.1554 |
| GBR | 91 | C: 75.27%, A: 24.73% | C: 42.31%, T: 57.69% | 0.2409 |
| GIH | 103 | C: 80.1%, A: 19.9% | C: 51.46%, T: 48.54% | 0.2634 |
| GWD | 113 | C: 73.45%, A: 26.55% | C: 17.7%, T: 82.3% | 0.0777 |
| IBS | 107 | C: 77.1%, A: 22.9% | C: 47.66%, T: 52.34% | 0.2705 |
| ITU | 102 | C: 74.02%, A: 25.98% | C: 45.59%, T: 54.41% | 0.2941 |
| JPT | 104 | C: 69.71%, A: 30.29% | C: 50.0%, T: 50.0% | 0.4345 |
| KHV | 99 | C: 72.73%, A: 27.27% | C: 55.56%, T: 44.44% | 0.4688 |
| LWK | 99 | C: 87.37%, A: 12.63% | C: 21.21%, T: 78.79% | 0.0389 |
| MSL | 85 | C: 74.71%, A: 25.29% | C: 18.82%, T: 81.18% | 0.0785 |
| MXL | 64 | C: 73.44%, A: 26.56% | C: 48.44%, T: 51.56% | 0.3398 |
| PEL | 85 | C: 82.35%, A: 17.65% | C: 59.41%, T: 40.59% | 0.3137 |
| PJL | 96 | C: 73.96%, A: 26.04% | C: 51.56%, T: 48.44% | 0.3748 |
| PUR | 104 | C: 74.52%, A: 25.48% | C: 39.9%, T: 60.1% | 0.227 |
| SAS | 489 | C: 74.74%, A: 25.26% | C: 48.26%, T: 51.74% | 0.3152 |
| STU | 102 | C: 74.51%, A: 25.49% | C: 45.59%, T: 54.41% | 0.2866 |
| TSI | 107 | C: 74.3%, A: 25.7% | C: 45.79%, T: 54.21% | 0.2922 |
| YRI | 108 | C: 76.85%, A: 23.15% | C: 18.06%, T: 81.94% | 0.0664 |

Abbreviations: ACB, African Caribbeans in Barbados; AFR, All African; ALL, All populations; AMR, Ad Mixed American; ASW, Americans of African Ancestry in SW USA; BEB, Bengali from Bangladesh; CDX, Chinese Dai in Xishuangbanna, China; CEU, Utah Residents (CEPH) with Northern and Western European Ancestry; CHB, Han Chinese in Beijing, China; CHS, Southern Han Chinese; CLM, Colombians from Medellin, Colombia; EAS, All East Asian; ESN, Esan in Nigeria; EUR, European; FIN, Finnish in Finland; GBR, British in England and Scotland; GIH, Gujarati Indian from Houston, Texas; GWD, Gambian in Western Divisions in the Gambia; IBS, Iberian Population in Spain; ITU, Indian Telugu from the UK; JPT, Japanese in Tokyo, Japan; KHV, Kinh in Ho Chi Minh City, Vietnam; LWK, Luhya in Webuye, Kenya; MSL, Mende in Sierra Leone; MXL, Mexican Ancestry from Los Angeles USA; PEL, Peruvians from Lima, Peru; PJL, Punjabi from Lahore, Pakistan; PUR, Puerto Ricans from Puerto Rico; SAS, South Asian; STU, Sri Lankan Tamil from the UK; TSI, Toscani in Italia; YRI, Yoruba in Ibadan, Nigeria.

**S13 Table.** Pairwise linkage disequilibrium (R^2^) for rs3138078 and rs3138076 CD14 SNPs.

| **Population** | **N** | **rs3138078 Allele Freq** | **rs3138076 Allele Freq** | **R^2^** |
| --- | --- | --- | --- | --- |
| ACB | 96 | C: 85.94%, A: 14.06% | T: 85.94%, C: 14.06% | 1 |
| AFR | 661 | C: 80.03%, A: 19.97% | T: 80.03%, C: 19.97% | 1 |
| ALL | 2504 | C: 75.7%, A: 24.3% | T: 75.6%, C: 24.4% | 0.9924 |
| AMR | 347 | C: 77.23%, A: 22.77% | T: 77.23%, C: 22.77% | 1 |
| ASW | 61 | C: 78.69%, A: 21.31% | T: 78.69%, C: 21.31% | 1 |
| BEB | 86 | C: 70.35%, A: 29.65% | T: 70.35%, C: 29.65% | 1 |
| CDX | 93 | C: 65.59%, A: 34.41% | T: 65.59%, C: 34.41% | 1 |
| CEU | 99 | C: 76.26%, A: 23.74% | T: 75.76%, C: 24.24% | 0.9727 |
| CHB | 103 | C: 73.3%, A: 26.7% | T: 73.3%, C: 26.7% | 1 |
| CHS | 105 | C: 70.48%, A: 29.52% | T: 70.48%, C: 29.52% | 1 |
| CLM | 94 | C: 78.19%, A: 21.81% | T: 78.19%, C: 21.81% | 1 |
| EAS | 504 | C: 70.44%, A: 29.56% | T: 70.44%, C: 29.56% | 1 |
| ESN | 99 | C: 83.33%, A: 16.67% | T: 83.33%, C: 16.67% | 1 |
| EUR | 503 | C: 75.15%, A: 24.85% | T: 74.65%, C: 25.35% | 0.9635 |
| FIN | 99 | C: 72.73%, A: 27.27% | T: 70.71%, C: 29.29% | 0.9052 |
| GBR | 91 | C: 75.27%, A: 24.73% | T: 75.27%, C: 24.73% | 1 |
| GIH | 103 | C: 80.1%, A: 19.9% | T: 80.1%, C: 19.9% | 1 |
| GWD | 113 | C: 73.45%, A: 26.55% | T: 73.45%, C: 26.55% | 1 |
| IBS | 107 | C: 77.1%, A: 22.9% | T: 77.1%, C: 22.9% | 1 |
| ITU | 102 | C: 74.02%, A: 25.98% | T: 74.02%, C: 25.98% | 1 |
| JPT | 104 | C: 69.71%, A: 30.29% | T: 69.71%, C: 30.29% | 1 |
| KHV | 99 | C: 72.73%, A: 27.27% | T: 72.73%, C: 27.27% | 1 |
| LWK | 99 | C: 87.37%, A: 12.63% | T: 87.37%, C: 12.63% | 1 |
| MSL | 85 | C: 74.71%, A: 25.29% | T: 74.71%, C: 25.29% | 1 |
| MXL | 64 | C: 73.44%, A: 26.56% | T: 73.44%, C: 26.56% | 1 |
| PEL | 85 | C: 82.35%, A: 17.65% | T: 82.35%, C: 17.65% | 1 |
| PJL | 96 | C: 73.96%, A: 26.04% | T: 73.96%, C: 26.04% | 1 |
| PUR | 104 | C: 74.52%, A: 25.48% | T: 74.52%, C: 25.48% | 1 |
| SAS | 489 | C: 74.74%, A: 25.26% | T: 74.74%, C: 25.26% | 1 |
| STU | 102 | C: 74.51%, A: 25.49% | T: 74.51%, C: 25.49% | 1 |
| TSI | 107 | C: 74.3%, A: 25.7% | T: 74.3%, C: 25.7% | 0.9517 |
| YRI | 108 | C: 76.85%, A: 23.15% | T: 76.85%, C: 23.15% | 1 |

Abbreviations: ACB, African Caribbeans in Barbados; AFR, All African; ALL, All populations; AMR, Ad Mixed American; ASW, Americans of African Ancestry in SW USA; BEB, Bengali from Bangladesh; CDX, Chinese Dai in Xishuangbanna, China; CEU, Utah Residents (CEPH) with Northern and Western European Ancestry; CHB, Han Chinese in Beijing, China; CHS, Southern Han Chinese; CLM, Colombians from Medellin, Colombia; EAS, All East Asian; ESN, Esan in Nigeria; EUR, European; FIN, Finnish in Finland; GBR, British in England and Scotland; GIH, Gujarati Indian from Houston, Texas; GWD, Gambian in Western Divisions in the Gambia; IBS, Iberian Population in Spain; ITU, Indian Telugu from the UK; JPT, Japanese in Tokyo, Japan; KHV, Kinh in Ho Chi Minh City, Vietnam; LWK, Luhya in Webuye, Kenya; MSL, Mende in Sierra Leone; MXL, Mexican Ancestry from Los Angeles USA; PEL, Peruvians from Lima, Peru; PJL, Punjabi from Lahore, Pakistan; PUR, Puerto Ricans from Puerto Rico; SAS, South Asian; STU, Sri Lankan Tamil from the UK; TSI, Toscani in Italia; YRI, Yoruba in Ibadan, Nigeria.

**S14 Table.** Pairwise linkage disequilibrium (R^2^) for rs3138078 and rs5744455 CD14 SNPs.

| **Population** | **N** | **rs3138078 Allele Freq** | **rs5744455 Allele Freq** | **R^2^** |
| --- | --- | --- | --- | --- |
| ACB | 96 | C: 85.94%, A: 14.06% | G: 94.27%, A: 5.73% | 0.3714 |
| AFR | 661 | C: 80.03%, A: 19.97% | G: 94.02%, A: 5.98% | 0.2547 |
| ALL | 2504 | C: 75.7%, A: 24.3% | G: 79.85%, A: 20.15% | 0.7819 |
| AMR | 347 | C: 77.23%, A: 22.77% | G: 79.83%, A: 20.17% | 0.8415 |
| ASW | 61 | C: 78.69%, A: 21.31% | G: 90.98%, A: 9.02% | 0.3659 |
| BEB | 86 | C: 70.35%, A: 29.65% | G: 70.35%, A: 29.65% | 1 |
| CDX | 93 | C: 65.59%, A: 34.41% | G: 65.59%, A: 34.41% | 1 |
| CEU | 99 | C: 76.26%, A: 23.74% | G: 77.78%, A: 22.22% | 0.9179 |
| CHB | 103 | C: 73.3%, A: 26.7% | G: 73.3%, A: 26.7% | 1 |
| CHS | 105 | C: 70.48%, A: 29.52% | G: 70.48%, A: 29.52% | 1 |
| CLM | 94 | C: 78.19%, A: 21.81% | G: 82.45%, A: 17.55% | 0.7633 |
| EAS | 504 | C: 70.44%, A: 29.56% | G: 70.44%, A: 29.56% | 1 |
| ESN | 99 | C: 83.33%, A: 16.67% | G: 93.94%, A: 6.06% | 0.3226 |
| EUR | 503 | C: 75.15%, A: 24.85% | G: 75.75%, A: 24.25% | 0.9683 |
| FIN | 99 | C: 72.73%, A: 27.27% | G: 72.73%, A: 27.27% | 1 |
| GBR | 91 | C: 75.27%, A: 24.73% | G: 75.27%, A: 24.73% | 1 |
| GIH | 103 | C: 80.1%, A: 19.9% | G: 79.61%, A: 20.39% | 0.9703 |
| GWD | 113 | C: 73.45%, A: 26.55% | G: 95.13%, A: 4.87% | 0.1416 |
| IBS | 107 | C: 77.1%, A: 22.9% | G: 77.57%, A: 22.43% | 0.9737 |
| ITU | 102 | C: 74.02%, A: 25.98% | G: 74.02%, A: 25.98% | 1 |
| JPT | 104 | C: 69.71%, A: 30.29% | G: 69.71%, A: 30.29% | 1 |
| KHV | 99 | C: 72.73%, A: 27.27% | G: 72.73%, A: 27.27% | 1 |
| LWK | 99 | C: 87.37%, A: 12.63% | G: 97.47%, A: 2.53% | 0.1793 |
| MSL | 85 | C: 74.71%, A: 25.29% | G: 92.94%, A: 7.06% | 0.2243 |
| MXL | 64 | C: 73.44%, A: 26.56% | G: 74.22%, A: 25.78% | 0.8827 |
| PEL | 85 | C: 82.35%, A: 17.65% | G: 83.53%, A: 16.47% | 0.9202 |
| PJL | 96 | C: 73.96%, A: 26.04% | G: 73.96%, A: 26.04% | 1 |
| PUR | 104 | C: 74.52%, A: 25.48% | G: 77.88%, A: 22.12% | 0.8304 |
| SAS | 489 | C: 74.74%, A: 25.26% | G: 74.64%, A: 25.36% | 0.9946 |
| STU | 102 | C: 74.51%, A: 25.49% | G: 74.51%, A: 25.49% | 1 |
| TSI | 107 | C: 74.3%, A: 25.7% | G: 75.23%, A: 24.77% | 0.9517 |
| YRI | 108 | C: 76.85%, A: 23.15% | G: 92.13%, A: 7.87% | 0.2836 |

Abbreviations: ACB, African Caribbeans in Barbados; AFR, All African; ALL, All populations; AMR, Ad Mixed American; ASW, Americans of African Ancestry in SW USA; BEB, Bengali from Bangladesh; CDX, Chinese Dai in Xishuangbanna, China; CEU, Utah Residents (CEPH) with Northern and Western European Ancestry; CHB, Han Chinese in Beijing, China; CHS, Southern Han Chinese; CLM, Colombians from Medellin, Colombia; EAS, All East Asian; ESN, Esan in Nigeria; EUR, European; FIN, Finnish in Finland; GBR, British in England and Scotland; GIH, Gujarati Indian from Houston, Texas; GWD, Gambian in Western Divisions in the Gambia; IBS, Iberian Population in Spain; ITU, Indian Telugu from the UK; JPT, Japanese in Tokyo, Japan; KHV, Kinh in Ho Chi Minh City, Vietnam; LWK, Luhya in Webuye, Kenya; MSL, Mende in Sierra Leone; MXL, Mexican Ancestry from Los Angeles USA; PEL, Peruvians from Lima, Peru; PJL, Punjabi from Lahore, Pakistan; PUR, Puerto Ricans from Puerto Rico; SAS, South Asian; STU, Sri Lankan Tamil from the UK; TSI, Toscani in Italia; YRI, Yoruba in Ibadan, Nigeria.

**S15 Table.** Pairwise linkage disequilibrium (R^2^) for rs3138078 and rs5744454 CD14 SNPs.

| **Population** | **N** | **rs3138078 Allele Freq** | **rs5744454 Allele Freq** | **R^2^** |
| --- | --- | --- | --- | --- |
| ACB | 96 | C: 85.94%, A: 14.06% | T: 85.94%, G: 14.06% | 1 |
| AFR | 661 | C: 80.03%, A: 19.97% | T: 80.03%, G: 19.97% | 1 |
| ALL | 2504 | C: 75.7%, A: 24.3% | T: 75.6%, G: 24.4% | 0.9924 |
| AMR | 347 | C: 77.23%, A: 22.77% | T: 77.23%, G: 22.77% | 1 |
| ASW | 61 | C: 78.69%, A: 21.31% | T: 78.69%, G: 21.31% | 1 |
| BEB | 86 | C: 70.35%, A: 29.65% | T: 70.35%, G: 29.65% | 1 |
| CDX | 93 | C: 65.59%, A: 34.41% | T: 65.59%, G: 34.41% | 1 |
| CEU | 99 | C: 76.26%, A: 23.74% | T: 75.76%, G: 24.24% | 0.9727 |
| CHB | 103 | C: 73.3%, A: 26.7% | T: 73.3%, G: 26.7% | 1 |
| CHS | 105 | C: 70.48%, A: 29.52% | T: 70.48%, G: 29.52% | 1 |
| CLM | 94 | C: 78.19%, A: 21.81% | T: 78.19%, G: 21.81% | 1 |
| EAS | 504 | C: 70.44%, A: 29.56% | T: 70.44%, G: 29.56% | 1 |
| ESN | 99 | C: 83.33%, A: 16.67% | T: 83.33%, G: 16.67% | 1 |
| EUR | 503 | C: 75.15%, A: 24.85% | T: 74.65%, G: 25.35% | 0.9635 |
| FIN | 99 | C: 72.73%, A: 27.27% | T: 70.71%, G: 29.29% | 0.9052 |
| GBR | 91 | C: 75.27%, A: 24.73% | T: 75.27%, G: 24.73% | 1 |
| GIH | 103 | C: 80.1%, A: 19.9% | T: 80.1%, G: 19.9% | 1 |
| GWD | 113 | C: 73.45%, A: 26.55% | T: 73.45%, G: 26.55% | 1 |
| IBS | 107 | C: 77.1%, A: 22.9% | T: 77.1%, G: 22.9% | 1 |
| ITU | 102 | C: 74.02%, A: 25.98% | T: 74.02%, G: 25.98% | 1 |
| JPT | 104 | C: 69.71%, A: 30.29% | T: 69.71%, G: 30.29% | 1 |
| KHV | 99 | C: 72.73%, A: 27.27% | T: 72.73%, G: 27.27% | 1 |
| LWK | 99 | C: 87.37%, A: 12.63% | T: 87.37%, G: 12.63% | 1 |
| MSL | 85 | C: 74.71%, A: 25.29% | T: 74.71%, G: 25.29% | 1 |
| MXL | 64 | C: 73.44%, A: 26.56% | T: 73.44%, G: 26.56% | 1 |
| PEL | 85 | C: 82.35%, A: 17.65% | T: 82.35%, G: 17.65% | 1 |
| PJL | 96 | C: 73.96%, A: 26.04% | T: 73.96%, G: 26.04% | 1 |
| PUR | 104 | C: 74.52%, A: 25.48% | T: 74.52%, G: 25.48% | 1 |
| SAS | 489 | C: 74.74%, A: 25.26% | T: 74.74%, G: 25.26% | 1 |
| STU | 102 | C: 74.51%, A: 25.49% | T: 74.51%, G: 25.49% | 1 |
| TSI | 107 | C: 74.3%, A: 25.7% | T: 74.3%, G: 25.7% | 0.9517 |
| YRI | 108 | C: 76.85%, A: 23.15% | T: 76.85%, G: 23.15% | 1 |

Abbreviations: ACB, African Caribbeans in Barbados; AFR, All African; ALL, All populations; AMR, Ad Mixed American; ASW, Americans of African Ancestry in SW USA; BEB, Bengali from Bangladesh; CDX, Chinese Dai in Xishuangbanna, China; CEU, Utah Residents (CEPH) with Northern and Western European Ancestry; CHB, Han Chinese in Beijing, China; CHS, Southern Han Chinese; CLM, Colombians from Medellin, Colombia; EAS, All East Asian; ESN, Esan in Nigeria; EUR, European; FIN, Finnish in Finland; GBR, British in England and Scotland; GIH, Gujarati Indian from Houston, Texas; GWD, Gambian in Western Divisions in the Gambia; IBS, Iberian Population in Spain; ITU, Indian Telugu from the UK; JPT, Japanese in Tokyo, Japan; KHV, Kinh in Ho Chi Minh City, Vietnam; LWK, Luhya in Webuye, Kenya; MSL, Mende in Sierra Leone; MXL, Mexican Ancestry from Los Angeles USA; PEL, Peruvians from Lima, Peru; PJL, Punjabi from Lahore, Pakistan; PUR, Puerto Ricans from Puerto Rico; SAS, South Asian; STU, Sri Lankan Tamil from the UK; TSI, Toscani in Italia; YRI, Yoruba in Ibadan, Nigeria.

**S16 Table.** Pairwise linkage disequilibrium (R^2^) for rs2915863 and rs3138076 CD14 SNPs.

| **Population** | **N** | **rs2915863 Allele Freq** | **rs3138076 Allele Freq** | **R^2^** |
| --- | --- | --- | --- | --- |
| ACB | 96 | C: 23.44%, T: 76.56% | T: 85.94%, C: 14.06% | 0.0501 |
| AFR | 661 | C: 18.46%, T: 81.54% | T: 80.03%, C: 19.97% | 0.0565 |
| ALL | 2504 | C: 39.76%, T: 60.24% | T: 75.6%, C: 24.4% | 0.213 |
| AMR | 347 | C: 44.81%, T: 55.19% | T: 77.23%, C: 22.77% | 0.2394 |
| ASW | 61 | C: 14.75%, T: 85.25% | T: 78.69%, C: 21.31% | 0.0469 |
| BEB | 86 | C: 47.09%, T: 52.91% | T: 70.35%, C: 29.65% | 0.3752 |
| CDX | 93 | C: 49.46%, T: 50.54% | T: 65.59%, C: 34.41% | 0.5134 |
| CEU | 99 | C: 44.95%, T: 55.05% | T: 75.76%, C: 24.24% | 0.2613 |
| CHB | 103 | C: 55.83%, T: 44.17% | T: 73.3%, C: 26.7% | 0.4603 |
| CHS | 105 | C: 56.67%, T: 43.33% | T: 70.48%, C: 29.52% | 0.5478 |
| CLM | 94 | C: 34.57%, T: 65.43% | T: 78.19%, C: 21.81% | 0.1474 |
| EAS | 504 | C: 53.57%, T: 46.43% | T: 70.44%, C: 29.56% | 0.4843 |
| ESN | 99 | C: 14.14%, T: 85.86% | T: 83.33%, C: 16.67% | 0.0329 |
| EUR | 503 | C: 42.15%, T: 57.85% | T: 74.65%, C: 25.35% | 0.2474 |
| FIN | 99 | C: 29.29%, T: 70.71% | T: 70.71%, C: 29.29% | 0.1716 |
| GBR | 91 | C: 42.31%, T: 57.69% | T: 75.27%, C: 24.73% | 0.2409 |
| GIH | 103 | C: 51.46%, T: 48.54% | T: 80.1%, C: 19.9% | 0.2634 |
| GWD | 113 | C: 17.7%, T: 82.3% | T: 73.45%, C: 26.55% | 0.0777 |
| IBS | 107 | C: 47.66%, T: 52.34% | T: 77.1%, C: 22.9% | 0.2705 |
| ITU | 102 | C: 45.59%, T: 54.41% | T: 74.02%, C: 25.98% | 0.2941 |
| JPT | 104 | C: 50.0%, T: 50.0% | T: 69.71%, C: 30.29% | 0.4345 |
| KHV | 99 | C: 55.56%, T: 44.44% | T: 72.73%, C: 27.27% | 0.4688 |
| LWK | 99 | C: 21.21%, T: 78.79% | T: 87.37%, C: 12.63% | 0.0389 |
| MSL | 85 | C: 18.82%, T: 81.18% | T: 74.71%, C: 25.29% | 0.0785 |
| MXL | 64 | C: 48.44%, T: 51.56% | T: 73.44%, C: 26.56% | 0.3398 |
| PEL | 85 | C: 59.41%, T: 40.59% | T: 82.35%, C: 17.65% | 0.3137 |
| PJL | 96 | C: 51.56%, T: 48.44% | T: 73.96%, C: 26.04% | 0.3748 |
| PUR | 104 | C: 39.9%, T: 60.1% | T: 74.52%, C: 25.48% | 0.227 |
| SAS | 489 | C: 48.26%, T: 51.74% | T: 74.74%, C: 25.26% | 0.3152 |
| STU | 102 | C: 45.59%, T: 54.41% | T: 74.51%, C: 25.49% | 0.2866 |
| TSI | 107 | C: 45.79%, T: 54.21% | T: 74.3%, C: 25.7% | 0.2922 |
| YRI | 108 | C: 18.06%, T: 81.94% | T: 76.85%, C: 23.15% | 0.0664 |

Abbreviations: ACB, African Caribbeans in Barbados; AFR, All African; ALL, All populations; AMR, Ad Mixed American; ASW, Americans of African Ancestry in SW USA; BEB, Bengali from Bangladesh; CDX, Chinese Dai in Xishuangbanna, China; CEU, Utah Residents (CEPH) with Northern and Western European Ancestry; CHB, Han Chinese in Beijing, China; CHS, Southern Han Chinese; CLM, Colombians from Medellin, Colombia; EAS, All East Asian; ESN, Esan in Nigeria; EUR, European; FIN, Finnish in Finland; GBR, British in England and Scotland; GIH, Gujarati Indian from Houston, Texas; GWD, Gambian in Western Divisions in the Gambia; IBS, Iberian Population in Spain; ITU, Indian Telugu from the UK; JPT, Japanese in Tokyo, Japan; KHV, Kinh in Ho Chi Minh City, Vietnam; LWK, Luhya in Webuye, Kenya; MSL, Mende in Sierra Leone; MXL, Mexican Ancestry from Los Angeles USA; PEL, Peruvians from Lima, Peru; PJL, Punjabi from Lahore, Pakistan; PUR, Puerto Ricans from Puerto Rico; SAS, South Asian; STU, Sri Lankan Tamil from the UK; TSI, Toscani in Italia; YRI, Yoruba in Ibadan, Nigeria.

**S17 Table.** Pairwise linkage disequilibrium (R^2^) for rs2915863 and rs5744455 CD14 SNPs.

| **Population** | **N** | **rs2915863 Allele Freq** | **rs5744455 Allele Freq** | **R^2^** |
| --- | --- | --- | --- | --- |
| ACB | 96 | C: 23.44%, T: 76.56% | G: 94.27%, A: 5.73% | 0.0186 |
| AFR | 661 | C: 18.46%, T: 81.54% | G: 94.02%, A: 5.98% | 0.0144 |
| ALL | 2504 | C: 39.76%, T: 60.24% | G: 79.85%, A: 20.15% | 0.1665 |
| AMR | 347 | C: 44.81%, T: 55.19% | G: 79.83%, A: 20.17% | 0.2052 |
| ASW | 61 | C: 14.75%, T: 85.25% | G: 90.98%, A: 9.02% | 0.0172 |
| BEB | 86 | C: 47.09%, T: 52.91% | G: 70.35%, A: 29.65% | 0.3752 |
| CDX | 93 | C: 49.46%, T: 50.54% | G: 65.59%, A: 34.41% | 0.5134 |
| CEU | 99 | C: 44.95%, T: 55.05% | G: 77.78%, A: 22.22% | 0.2333 |
| CHB | 103 | C: 55.83%, T: 44.17% | G: 73.3%, A: 26.7% | 0.4603 |
| CHS | 105 | C: 56.67%, T: 43.33% | G: 70.48%, A: 29.52% | 0.5478 |
| CLM | 94 | C: 34.57%, T: 65.43% | G: 82.45%, A: 17.55% | 0.1125 |
| EAS | 504 | C: 53.57%, T: 46.43% | G: 70.44%, A: 29.56% | 0.4843 |
| ESN | 99 | C: 14.14%, T: 85.86% | G: 93.94%, A: 6.06% | 0.0106 |
| EUR | 503 | C: 42.15%, T: 57.85% | G: 75.75%, A: 24.25% | 0.2333 |
| FIN | 99 | C: 29.29%, T: 70.71% | G: 72.73%, A: 27.27% | 0.1554 |
| GBR | 91 | C: 42.31%, T: 57.69% | G: 75.27%, A: 24.73% | 0.2409 |
| GIH | 103 | C: 51.46%, T: 48.54% | G: 79.61%, A: 20.39% | 0.2715 |
| GWD | 113 | C: 17.7%, T: 82.3% | G: 95.13%, A: 4.87% | 0.011 |
| IBS | 107 | C: 47.66%, T: 52.34% | G: 77.57%, A: 22.43% | 0.2633 |
| ITU | 102 | C: 45.59%, T: 54.41% | G: 74.02%, A: 25.98% | 0.2941 |
| JPT | 104 | C: 50.0%, T: 50.0% | G: 69.71%, A: 30.29% | 0.4345 |
| KHV | 99 | C: 55.56%, T: 44.44% | G: 72.73%, A: 27.27% | 0.4688 |
| LWK | 99 | C: 21.21%, T: 78.79% | G: 97.47%, A: 2.53% | 0.007 |
| MSL | 85 | C: 18.82%, T: 81.18% | G: 92.94%, A: 7.06% | 0.0176 |
| MXL | 64 | C: 48.44%, T: 51.56% | G: 74.22%, A: 25.78% | 0.3263 |
| PEL | 85 | C: 59.41%, T: 40.59% | G: 83.53%, A: 16.47% | 0.2886 |
| PJL | 96 | C: 51.56%, T: 48.44% | G: 73.96%, A: 26.04% | 0.3748 |
| PUR | 104 | C: 39.9%, T: 60.1% | G: 77.88%, A: 22.12% | 0.1885 |
| SAS | 489 | C: 48.26%, T: 51.74% | G: 74.64%, A: 25.36% | 0.3169 |
| STU | 102 | C: 45.59%, T: 54.41% | G: 74.51%, A: 25.49% | 0.2866 |
| TSI | 107 | C: 45.79%, T: 54.21% | G: 75.23%, A: 24.77% | 0.2781 |
| YRI | 108 | C: 18.06%, T: 81.94% | G: 92.13%, A: 7.87% | 0.0188 |

Abbreviations: ACB, African Caribbeans in Barbados; AFR, All African; ALL, All populations; AMR, Ad Mixed American; ASW, Americans of African Ancestry in SW USA; BEB, Bengali from Bangladesh; CDX, Chinese Dai in Xishuangbanna, China; CEU, Utah Residents (CEPH) with Northern and Western European Ancestry; CHB, Han Chinese in Beijing, China; CHS, Southern Han Chinese; CLM, Colombians from Medellin, Colombia; EAS, All East Asian; ESN, Esan in Nigeria; EUR, European; FIN, Finnish in Finland; GBR, British in England and Scotland; GIH, Gujarati Indian from Houston, Texas; GWD, Gambian in Western Divisions in the Gambia; IBS, Iberian Population in Spain; ITU, Indian Telugu from the UK; JPT, Japanese in Tokyo, Japan; KHV, Kinh in Ho Chi Minh City, Vietnam; LWK, Luhya in Webuye, Kenya; MSL, Mende in Sierra Leone; MXL, Mexican Ancestry from Los Angeles USA; PEL, Peruvians from Lima, Peru; PJL, Punjabi from Lahore, Pakistan; PUR, Puerto Ricans from Puerto Rico; SAS, South Asian; STU, Sri Lankan Tamil from the UK; TSI, Toscani in Italia; YRI, Yoruba in Ibadan, Nigeria.

**S18 Table.** Pairwise linkage disequilibrium (R^2^) for rs2915863 and rs5744454 CD14 SNPs.

| **Population** | **N** | **rs2915863 Allele Freq** | **rs5744454 Allele Freq** | **R^2^** |
| --- | --- | --- | --- | --- |
| ACB | 96 | C: 23.44%, T: 76.56% | T: 85.94%, G: 14.06% | 0.0501 |
| AFR | 661 | C: 18.46%, T: 81.54% | T: 80.03%, G: 19.97% | 0.0565 |
| ALL | 2504 | C: 39.76%, T: 60.24% | T: 75.6%, G: 24.4% | 0.213 |
| AMR | 347 | C: 44.81%, T: 55.19% | T: 77.23%, G: 22.77% | 0.2394 |
| ASW | 61 | C: 14.75%, T: 85.25% | T: 78.69%, G: 21.31% | 0.0469 |
| BEB | 86 | C: 47.09%, T: 52.91% | T: 70.35%, G: 29.65% | 0.3752 |
| CDX | 93 | C: 49.46%, T: 50.54% | T: 65.59%, G: 34.41% | 0.5134 |
| CEU | 99 | C: 44.95%, T: 55.05% | T: 75.76%, G: 24.24% | 0.2613 |
| CHB | 103 | C: 55.83%, T: 44.17% | T: 73.3%, G: 26.7% | 0.4603 |
| CHS | 105 | C: 56.67%, T: 43.33% | T: 70.48%, G: 29.52% | 0.5478 |
| CLM | 94 | C: 34.57%, T: 65.43% | T: 78.19%, G: 21.81% | 0.1474 |
| EAS | 504 | C: 53.57%, T: 46.43% | T: 70.44%, G: 29.56% | 0.4843 |
| ESN | 99 | C: 14.14%, T: 85.86% | T: 83.33%, G: 16.67% | 0.0329 |
| EUR | 503 | C: 42.15%, T: 57.85% | T: 74.65%, G: 25.35% | 0.2474 |
| FIN | 99 | C: 29.29%, T: 70.71% | T: 70.71%, G: 29.29% | 0.1716 |
| GBR | 91 | C: 42.31%, T: 57.69% | T: 75.27%, G: 24.73% | 0.2409 |
| GIH | 103 | C: 51.46%, T: 48.54% | T: 80.1%, G: 19.9% | 0.2634 |
| GWD | 113 | C: 17.7%, T: 82.3% | T: 73.45%, G: 26.55% | 0.0777 |
| IBS | 107 | C: 47.66%, T: 52.34% | T: 77.1%, G: 22.9% | 0.2705 |
| ITU | 102 | C: 45.59%, T: 54.41% | T: 74.02%, G: 25.98% | 0.2941 |
| JPT | 104 | C: 50.0%, T: 50.0% | T: 69.71%, G: 30.29% | 0.4345 |
| KHV | 99 | C: 55.56%, T: 44.44% | T: 72.73%, G: 27.27% | 0.4688 |
| LWK | 99 | C: 21.21%, T: 78.79% | T: 87.37%, G: 12.63% | 0.0389 |
| MSL | 85 | C: 18.82%, T: 81.18% | T: 74.71%, G: 25.29% | 0.0785 |
| MXL | 64 | C: 48.44%, T: 51.56% | T: 73.44%, G: 26.56% | 0.3398 |
| PEL | 85 | C: 59.41%, T: 40.59% | T: 82.35%, G: 17.65% | 0.3137 |
| PJL | 96 | C: 51.56%, T: 48.44% | T: 73.96%, G: 26.04% | 0.3748 |
| PUR | 104 | C: 39.9%, T: 60.1% | T: 74.52%, G: 25.48% | 0.227 |
| SAS | 489 | C: 48.26%, T: 51.74% | T: 74.74%, G: 25.26% | 0.3152 |
| STU | 102 | C: 45.59%, T: 54.41% | T: 74.51%, G: 25.49% | 0.2866 |
| TSI | 107 | C: 45.79%, T: 54.21% | T: 74.3%, G: 25.7% | 0.2922 |
| YRI | 108 | C: 18.06%, T: 81.94% | T: 76.85%, G: 23.15% | 0.0664 |

Abbreviations: ACB, African Caribbeans in Barbados; AFR, All African; ALL, All populations; AMR, Ad Mixed American; ASW, Americans of African Ancestry in SW USA; BEB, Bengali from Bangladesh; CDX, Chinese Dai in Xishuangbanna, China; CEU, Utah Residents (CEPH) with Northern and Western European Ancestry; CHB, Han Chinese in Beijing, China; CHS, Southern Han Chinese; CLM, Colombians from Medellin, Colombia; EAS, All East Asian; ESN, Esan in Nigeria; EUR, European; FIN, Finnish in Finland; GBR, British in England and Scotland; GIH, Gujarati Indian from Houston, Texas; GWD, Gambian in Western Divisions in the Gambia; IBS, Iberian Population in Spain; ITU, Indian Telugu from the UK; JPT, Japanese in Tokyo, Japan; KHV, Kinh in Ho Chi Minh City, Vietnam; LWK, Luhya in Webuye, Kenya; MSL, Mende in Sierra Leone; MXL, Mexican Ancestry from Los Angeles USA; PEL, Peruvians from Lima, Peru; PJL, Punjabi from Lahore, Pakistan; PUR, Puerto Ricans from Puerto Rico; SAS, South Asian; STU, Sri Lankan Tamil from the UK; TSI, Toscani in Italia; YRI, Yoruba in Ibadan, Nigeria.

**S19 Table.** Pairwise linkage disequilibrium (R^2^) for rs3138076 and rs5744455 CD14 SNPs.

| **Population** | **N** | **rs3138076 Allele Freq** | **rs5744455 Allele Freq** | **R^2^** |
| --- | --- | --- | --- | --- |
| ACB | 96 | T: 85.94%, C: 14.06% | G: 94.27%, A: 5.73% | 0.3714 |
| AFR | 661 | T: 80.03%, C: 19.97% | G: 94.02%, A: 5.98% | 0.2547 |
| ALL | 2504 | T: 75.6%, C: 24.4% | G: 79.85%, A: 20.15% | 0.7776 |
| AMR | 347 | T: 77.23%, C: 22.77% | G: 79.83%, A: 20.17% | 0.8415 |
| ASW | 61 | T: 78.69%, C: 21.31% | G: 90.98%, A: 9.02% | 0.3659 |
| BEB | 86 | T: 70.35%, C: 29.65% | G: 70.35%, A: 29.65% | 1 |
| CDX | 93 | T: 65.59%, C: 34.41% | G: 65.59%, A: 34.41% | 1 |
| CEU | 99 | T: 75.76%, C: 24.24% | G: 77.78%, A: 22.22% | 0.8929 |
| CHB | 103 | T: 73.3%, C: 26.7% | G: 73.3%, A: 26.7% | 1 |
| CHS | 105 | T: 70.48%, C: 29.52% | G: 70.48%, A: 29.52% | 1 |
| CLM | 94 | T: 78.19%, C: 21.81% | G: 82.45%, A: 17.55% | 0.7633 |
| EAS | 504 | T: 70.44%, C: 29.56% | G: 70.44%, A: 29.56% | 1 |
| ESN | 99 | T: 83.33%, C: 16.67% | G: 93.94%, A: 6.06% | 0.3226 |
| EUR | 503 | T: 74.65%, C: 25.35% | G: 75.75%, A: 24.25% | 0.943 |
| FIN | 99 | T: 70.71%, C: 29.29% | G: 72.73%, A: 27.27% | 0.9052 |
| GBR | 91 | T: 75.27%, C: 24.73% | G: 75.27%, A: 24.73% | 1 |
| GIH | 103 | T: 80.1%, C: 19.9% | G: 79.61%, A: 20.39% | 0.9703 |
| GWD | 113 | T: 73.45%, C: 26.55% | G: 95.13%, A: 4.87% | 0.1416 |
| IBS | 107 | T: 77.1%, C: 22.9% | G: 77.57%, A: 22.43% | 0.9737 |
| ITU | 102 | T: 74.02%, C: 25.98% | G: 74.02%, A: 25.98% | 1 |
| JPT | 104 | T: 69.71%, C: 30.29% | G: 69.71%, A: 30.29% | 1 |
| KHV | 99 | T: 72.73%, C: 27.27% | G: 72.73%, A: 27.27% | 1 |
| LWK | 99 | T: 87.37%, C: 12.63% | G: 97.47%, A: 2.53% | 0.1793 |
| MSL | 85 | T: 74.71%, C: 25.29% | G: 92.94%, A: 7.06% | 0.2243 |
| MXL | 64 | T: 73.44%, C: 26.56% | G: 74.22%, A: 25.78% | 0.8827 |
| PEL | 85 | T: 82.35%, C: 17.65% | G: 83.53%, A: 16.47% | 0.9202 |
| PJL | 96 | T: 73.96%, C: 26.04% | G: 73.96%, A: 26.04% | 1 |
| PUR | 104 | T: 74.52%, C: 25.48% | G: 77.88%, A: 22.12% | 0.8304 |
| SAS | 489 | T: 74.74%, C: 25.26% | G: 74.64%, A: 25.36% | 0.9946 |
| STU | 102 | T: 74.51%, C: 25.49% | G: 74.51%, A: 25.49% | 1 |
| TSI | 107 | T: 74.3%, C: 25.7% | G: 75.23%, A: 24.77% | 0.9517 |
| YRI | 108 | T: 76.85%, C: 23.15% | G: 92.13%, A: 7.87% | 0.2836 |

Abbreviations: ACB, African Caribbeans in Barbados; AFR, All African; ALL, All populations; AMR, Ad Mixed American; ASW, Americans of African Ancestry in SW USA; BEB, Bengali from Bangladesh; CDX, Chinese Dai in Xishuangbanna, China; CEU, Utah Residents (CEPH) with Northern and Western European Ancestry; CHB, Han Chinese in Beijing, China; CHS, Southern Han Chinese; CLM, Colombians from Medellin, Colombia; EAS, All East Asian; ESN, Esan in Nigeria; EUR, European; FIN, Finnish in Finland; GBR, British in England and Scotland; GIH, Gujarati Indian from Houston, Texas; GWD, Gambian in Western Divisions in the Gambia; IBS, Iberian Population in Spain; ITU, Indian Telugu from the UK; JPT, Japanese in Tokyo, Japan; KHV, Kinh in Ho Chi Minh City, Vietnam; LWK, Luhya in Webuye, Kenya; MSL, Mende in Sierra Leone; MXL, Mexican Ancestry from Los Angeles USA; PEL, Peruvians from Lima, Peru; PJL, Punjabi from Lahore, Pakistan; PUR, Puerto Ricans from Puerto Rico; SAS, South Asian; STU, Sri Lankan Tamil from the UK; TSI, Toscani in Italia; YRI, Yoruba in Ibadan, Nigeria.

**S20 Table.** Pairwise linkage disequilibrium (R^2^) for rs3138076 and rs5744454 CD14 SNPs.

| **Population** | **N** | **rs3138076 Allele Freq** | **rs5744454 Allele Freq** | **R^2^** |
| --- | --- | --- | --- | --- |
| ACB | 96 | T: 85.94%, C: 14.06% | T: 85.94%, G: 14.06% | 1 |
| AFR | 661 | T: 80.03%, C: 19.97% | T: 80.03%, G: 19.97% | 1 |
| ALL | 2504 | T: 75.6%, C: 24.4% | T: 75.6%, G: 24.4% | 1 |
| AMR | 347 | T: 77.23%, C: 22.77% | T: 77.23%, G: 22.77% | 1 |
| ASW | 61 | T: 78.69%, C: 21.31% | T: 78.69%, G: 21.31% | 1 |
| BEB | 86 | T: 70.35%, C: 29.65% | T: 70.35%, G: 29.65% | 1 |
| CDX | 93 | T: 65.59%, C: 34.41% | T: 65.59%, G: 34.41% | 1 |
| CEU | 99 | T: 75.76%, C: 24.24% | T: 75.76%, G: 24.24% | 1 |
| CHB | 103 | T: 73.3%, C: 26.7% | T: 73.3%, G: 26.7% | 1 |
| CHS | 105 | T: 70.48%, C: 29.52% | T: 70.48%, G: 29.52% | 1 |
| CLM | 94 | T: 78.19%, C: 21.81% | T: 78.19%, G: 21.81% | 1 |
| EAS | 504 | T: 70.44%, C: 29.56% | T: 70.44%, G: 29.56% | 1 |
| ESN | 99 | T: 83.33%, C: 16.67% | T: 83.33%, G: 16.67% | 1 |
| EUR | 503 | T: 74.65%, C: 25.35% | T: 74.65%, G: 25.35% | 1 |
| FIN | 99 | T: 70.71%, C: 29.29% | T: 70.71%, G: 29.29% | 1 |
| GBR | 91 | T: 75.27%, C: 24.73% | T: 75.27%, G: 24.73% | 1 |
| GIH | 103 | T: 80.1%, C: 19.9% | T: 80.1%, G: 19.9% | 1 |
| GWD | 113 | T: 73.45%, C: 26.55% | T: 73.45%, G: 26.55% | 1 |
| IBS | 107 | T: 77.1%, C: 22.9% | T: 77.1%, G: 22.9% | 1 |
| ITU | 102 | T: 74.02%, C: 25.98% | T: 74.02%, G: 25.98% | 1 |
| JPT | 104 | T: 69.71%, C: 30.29% | T: 69.71%, G: 30.29% | 1 |
| KHV | 99 | T: 72.73%, C: 27.27% | T: 72.73%, G: 27.27% | 1 |
| LWK | 99 | T: 87.37%, C: 12.63% | T: 87.37%, G: 12.63% | 1 |
| MSL | 85 | T: 74.71%, C: 25.29% | T: 74.71%, G: 25.29% | 1 |
| MXL | 64 | T: 73.44%, C: 26.56% | T: 73.44%, G: 26.56% | 1 |
| PEL | 85 | T: 82.35%, C: 17.65% | T: 82.35%, G: 17.65% | 1 |
| PJL | 96 | T: 73.96%, C: 26.04% | T: 73.96%, G: 26.04% | 1 |
| PUR | 104 | T: 74.52%, C: 25.48% | T: 74.52%, G: 25.48% | 1 |
| SAS | 489 | T: 74.74%, C: 25.26% | T: 74.74%, G: 25.26% | 1 |
| STU | 102 | T: 74.51%, C: 25.49% | T: 74.51%, G: 25.49% | 1 |
| TSI | 107 | T: 74.3%, C: 25.7% | T: 74.3%, G: 25.7% | 1 |
| YRI | 108 | T: 76.85%, C: 23.15% | T: 76.85%, G: 23.15% | 1 |

Abbreviations: ACB, African Caribbeans in Barbados; AFR, All African; ALL, All populations; AMR, Ad Mixed American; ASW, Americans of African Ancestry in SW USA; BEB, Bengali from Bangladesh; CDX, Chinese Dai in Xishuangbanna, China; CEU, Utah Residents (CEPH) with Northern and Western European Ancestry; CHB, Han Chinese in Beijing, China; CHS, Southern Han Chinese; CLM, Colombians from Medellin, Colombia; EAS, All East Asian; ESN, Esan in Nigeria; EUR, European; FIN, Finnish in Finland; GBR, British in England and Scotland; GIH, Gujarati Indian from Houston, Texas; GWD, Gambian in Western Divisions in the Gambia; IBS, Iberian Population in Spain; ITU, Indian Telugu from the UK; JPT, Japanese in Tokyo, Japan; KHV, Kinh in Ho Chi Minh City, Vietnam; LWK, Luhya in Webuye, Kenya; MSL, Mende in Sierra Leone; MXL, Mexican Ancestry from Los Angeles USA; PEL, Peruvians from Lima, Peru; PJL, Punjabi from Lahore, Pakistan; PUR, Puerto Ricans from Puerto Rico; SAS, South Asian; STU, Sri Lankan Tamil from the UK; TSI, Toscani in Italia; YRI, Yoruba in Ibadan, Nigeria.

**S21 Table.** Pairwise linkage disequilibrium (R^2^) for rs5744455 and rs5744454 CD14 SNPs.

| **Population** | **N** | **rs5744455 Allele Freq** | **rs5744454 Allele Freq** | **R^2^** |
| --- | --- | --- | --- | --- |
| ACB | 96 | G: 94.27%, A: 5.73% | T: 85.94%, G: 14.06% | 0.3714 |
| AFR | 661 | G: 94.02%, A: 5.98% | T: 80.03%, G: 19.97% | 0.2547 |
| ALL | 2504 | G: 79.85%, A: 20.15% | T: 75.6%, G: 24.4% | 0.7776 |
| AMR | 347 | G: 79.83%, A: 20.17% | T: 77.23%, G: 22.77% | 0.8415 |
| ASW | 61 | G: 90.98%, A: 9.02% | T: 78.69%, G: 21.31% | 0.3659 |
| BEB | 86 | G: 70.35%, A: 29.65% | T: 70.35%, G: 29.65% | 1 |
| CDX | 93 | G: 65.59%, A: 34.41% | T: 65.59%, G: 34.41% | 1 |
| CEU | 99 | G: 77.78%, A: 22.22% | T: 75.76%, G: 24.24% | 0.8929 |
| CHB | 103 | G: 73.3%, A: 26.7% | T: 73.3%, G: 26.7% | 1 |
| CHS | 105 | G: 70.48%, A: 29.52% | T: 70.48%, G: 29.52% | 1 |
| CLM | 94 | G: 82.45%, A: 17.55% | T: 78.19%, G: 21.81% | 0.7633 |
| EAS | 504 | G: 70.44%, A: 29.56% | T: 70.44%, G: 29.56% | 1 |
| ESN | 99 | G: 93.94%, A: 6.06% | T: 83.33%, G: 16.67% | 0.3226 |
| EUR | 503 | G: 75.75%, A: 24.25% | T: 74.65%, G: 25.35% | 0.943 |
| FIN | 99 | G: 72.73%, A: 27.27% | T: 70.71%, G: 29.29% | 0.9052 |
| GBR | 91 | G: 75.27%, A: 24.73% | T: 75.27%, G: 24.73% | 1 |
| GIH | 103 | G: 79.61%, A: 20.39% | T: 80.1%, G: 19.9% | 0.9703 |
| GWD | 113 | G: 95.13%, A: 4.87% | T: 73.45%, G: 26.55% | 0.1416 |
| IBS | 107 | G: 77.57%, A: 22.43% | T: 77.1%, G: 22.9% | 0.9737 |
| ITU | 102 | G: 74.02%, A: 25.98% | T: 74.02%, G: 25.98% | 1 |
| JPT | 104 | G: 69.71%, A: 30.29% | T: 69.71%, G: 30.29% | 1 |
| KHV | 99 | G: 72.73%, A: 27.27% | T: 72.73%, G: 27.27% | 1 |
| LWK | 99 | G: 97.47%, A: 2.53% | T: 87.37%, G: 12.63% | 0.1793 |
| MSL | 85 | G: 92.94%, A: 7.06% | T: 74.71%, G: 25.29% | 0.2243 |
| MXL | 64 | G: 74.22%, A: 25.78% | T: 73.44%, G: 26.56% | 0.8827 |
| PEL | 85 | G: 83.53%, A: 16.47% | T: 82.35%, G: 17.65% | 0.9202 |
| PJL | 96 | G: 73.96%, A: 26.04% | T: 73.96%, G: 26.04% | 1 |
| PUR | 104 | G: 77.88%, A: 22.12% | T: 74.52%, G: 25.48% | 0.8304 |
| SAS | 489 | G: 74.64%, A: 25.36% | T: 74.74%, G: 25.26% | 0.9946 |
| STU | 102 | G: 74.51%, A: 25.49% | T: 74.51%, G: 25.49% | 1 |
| TSI | 107 | G: 75.23%, A: 24.77% | T: 74.3%, G: 25.7% | 0.9517 |
| YRI | 108 | G: 92.13%, A: 7.87% | T: 76.85%, G: 23.15% | 0.2836 |

Abbreviations: ACB, African Caribbeans in Barbados; AFR, All African; ALL, All populations; AMR, Ad Mixed American; ASW, Americans of African Ancestry in SW USA; BEB, Bengali from Bangladesh; CDX, Chinese Dai in Xishuangbanna, China; CEU, Utah Residents (CEPH) with Northern and Western European Ancestry; CHB, Han Chinese in Beijing, China; CHS, Southern Han Chinese; CLM, Colombians from Medellin, Colombia; EAS, All East Asian; ESN, Esan in Nigeria; EUR, European; FIN, Finnish in Finland; GBR, British in England and Scotland; GIH, Gujarati Indian from Houston, Texas; GWD, Gambian in Western Divisions in the Gambia; IBS, Iberian Population in Spain; ITU, Indian Telugu from the UK; JPT, Japanese in Tokyo, Japan; KHV, Kinh in Ho Chi Minh City, Vietnam; LWK, Luhya in Webuye, Kenya; MSL, Mende in Sierra Leone; MXL, Mexican Ancestry from Los Angeles USA; PEL, Peruvians from Lima, Peru; PJL, Punjabi from Lahore, Pakistan; PUR, Puerto Ricans from Puerto Rico; SAS, South Asian; STU, Sri Lankan Tamil from the UK; TSI, Toscani in Italia; YRI, Yoruba in Ibadan, Nigeria.
