# Supplementary material for: The influence of single nucleotide polymorphisms of NOD2 or CD14 on the risk of Mycobacterium tuberculosis diseases: a systematic review
Source: Syst Rev. 2021 Jun 9;10:174. doi: 10.1186/s13643-021-01729-y (PMC8191055; doi:10.1186/s13643-021-01729-y)
Supplement: Supplementary file 4 — Additional file 4. Tables depicting the frequencies and the linkage disequilibrium of the nine NOD2 SNPs. [file 13643_2021_1729_MOESM4_ESM.docx]

**Additional File 4**

The following Tables shows the frequencies of the nine NOD2 SNPs: rs1861759, rs7194886, rs9302752, rs2066842, rs2066844, rs5743278, rs17313265, rs6500328 and rs2111234. The Linkage Disequilibrium (LD) are expressed by the parameters R^2^ and calculate using Package LDpop.

**S1 Table.** Pairwise linkage disequilibrium (R^2^) for rs1861759 and rs7194886 NOD2 SNPs.

| **Population** | **N** | **rs1861759 Allele Freq** | **rs7194886 Allele Freq** | **R^2^** |
| --- | --- | --- | --- | --- |
| ALL | 2504 | T: 78.39%, G: 21.61% | C: 74.36%, T: 25.64% | 0.3702 |
| AFR | 661 | T: 84.11%, G: 15.89% | C: 74.21%, T: 25.79% | 0.0512 |
| YRI | 108 | T: 84.26%, G: 15.74% | C: 77.31%, T: 22.69% | 0.0169 |
| LWK | 99 | T: 85.86%, G: 14.14% | C: 77.78%, T: 22.22% | 0.1686 |
| GWD | 113 | T: 81.86%, G: 18.14% | C: 72.12%, T: 27.88% | 0.0016 |
| MSL | 85 | T: 86.47%, G: 13.53% | C: 78.24%, T: 21.76% | 0.0277 |
| ESN | 99 | T: 85.35%, G: 14.65% | C: 72.73%, T: 27.27% | 0.0517 |
| ASW | 61 | T: 84.43%, G: 15.57% | C: 72.95%, T: 27.05% | 0.0612 |
| ACB | 96 | T: 81.25%, G: 18.75% | C: 68.23%, T: 31.77% | 0.1511 |
| AMR | 347 | T: 72.77%, G: 27.23% | C: 69.88%, T: 30.12% | 0.5497 |
| MXL | 64 | T: 75.78%, G: 24.22% | C: 76.56%, T: 23.44% | 0.5829 |
| PUR | 104 | T: 71.63%, G: 28.37% | C: 63.46%, T: 36.54% | 0.485 |
| CLM | 94 | T: 72.87%, G: 27.13% | C: 68.62%, T: 31.38% | 0.5209 |
| PEL | 85 | T: 71.76%, G: 28.24% | C: 74.12%, T: 25.88% | 0.6769 |
| EAS | 504 | T: 90.08%, G: 9.92% | C: 90.48%, T: 9.52% | 0.0054 |
| CHB | 103 | T: 87.38%, G: 12.62% | C: 89.81%, T: 10.19% | 0.0064 |
| JPT | 104 | T: 86.06%, G: 13.94% | C: 90.87%, T: 9.13% | 0.0163 |
| CHS | 105 | T: 88.1%, G: 11.9% | C: 88.57%, T: 11.43% | 0.0074 |
| CDX | 93 | T: 93.55%, G: 6.45% | C: 91.4%, T: 8.6% | 0 |
| KHV | 99 | T: 95.96%, G: 4.04% | C: 91.92%, T: 8.08% | 0.0037 |
| EUR | 503 | T: 58.35%, G: 41.65% | C: 55.57%, T: 44.43% | 0.7742 |
| CEU | 99 | T: 63.64%, G: 36.36% | C: 63.64%, T: 36.36% | 0.833 |
| TSI | 107 | T: 52.34%, G: 47.66% | C: 52.34%, T: 47.66% | 0.7878 |
| FIN | 99 | T: 55.05%, G: 44.95% | C: 46.97%, T: 53.03% | 0.689 |
| GBR | 91 | T: 58.24%, G: 41.76% | C: 56.04%, T: 43.96% | 0.8717 |
| IBS | 107 | T: 62.62%, G: 37.38% | C: 58.88%, T: 41.12% | 0.7158 |
| SAS | 489 | T: 83.23%, G: 16.77% | C: 80.47%, T: 19.53% | 0.7208 |
| GIH | 103 | T: 83.5%, G: 16.5% | C: 78.16%, T: 21.84% | 0.7072 |
| PJL | 96 | T: 79.69%, G: 20.31% | C: 78.12%, T: 21.88% | 0.8516 |
| BEB | 86 | T: 86.63%, G: 13.37% | C: 83.14%, T: 16.86% | 0.6836 |
| STU | 102 | T: 83.33%, G: 16.67% | C: 81.37%, T: 18.63% | 0.6945 |
| ITU | 102 | T: 83.33%, G: 16.67% | C: 81.86%, T: 18.14% | 0.6619 |

Abbreviations: ACB, African Caribbeans in Barbados; AFR, All African; ALL, All populations; AMR, Ad Mixed American; ASW, Americans of African Ancestry in SW USA; BEB, Bengali from Bangladesh; CDX, Chinese Dai in Xishuangbanna, China; CEU, Utah Residents (CEPH) with Northern and Western European Ancestry; CHB, Han Chinese in Beijing, China; CHS, Southern Han Chinese; CLM, Colombians from Medellin, Colombia; EAS, All East Asian; ESN, Esan in Nigeria; EUR, European; FIN, Finnish in Finland; GBR, British in England and Scotland; GIH, Gujarati Indian from Houston, Texas; GWD, Gambian in Western Divisions in the Gambia; IBS, Iberian Population in Spain; ITU, Indian Telugu from the UK; JPT, Japanese in Tokyo, Japan; KHV, Kinh in Ho Chi Minh City, Vietnam; LWK, Luhya in Webuye, Kenya; MSL, Mende in Sierra Leone; MXL, Mexican Ancestry from Los Angeles USA; PEL, Peruvians from Lima, Peru; PJL, Punjabi from Lahore, Pakistan; PUR, Puerto Ricans from Puerto Rico; SAS, South Asian; STU, Sri Lankan Tamil from the UK; TSI, Toscani in Italia; YRI, Yoruba in Ibadan, Nigeria.

**S2 Table.** Pairwise linkage disequilibrium (R^2^) for rs1861759 and rs9302752 NOD2 SNPs.

| **Population** | **N** | **rs1861759 Allele Freq** | **rs9302752 Allele Freq** | **R^2^** |
| --- | --- | --- | --- | --- |
| ALL | 2504 | T: 78.39%, G: 21.61% | T: 50.02%, C: 49.98% | 0.2291 |
| AFR | 661 | T: 84.11%, G: 15.89% | T: 34.95%, C: 65.05% | 0.0805 |
| YRI | 108 | T: 84.26%, G: 15.74% | T: 39.81%, C: 60.19% | 0.0613 |
| LWK | 99 | T: 85.86%, G: 14.14% | T: 34.85%, C: 65.15% | 0.071 |
| GWD | 113 | T: 81.86%, G: 18.14% | T: 31.86%, C: 68.14% | 0.0743 |
| MSL | 85 | T: 86.47%, G: 13.53% | T: 41.76%, C: 58.24% | 0.1122 |
| ESN | 99 | T: 85.35%, G: 14.65% | T: 30.3%, C: 69.7% | 0.0586 |
| ASW | 61 | T: 84.43%, G: 15.57% | T: 35.25%, C: 64.75% | 0.1004 |
| ACB | 96 | T: 81.25%, G: 18.75% | T: 31.77%, C: 68.23% | 0.1075 |
| AMR | 347 | T: 72.77%, G: 27.23% | T: 45.1%, C: 54.9% | 0.2932 |
| MXL | 64 | T: 75.78%, G: 24.22% | T: 57.03%, C: 42.97% | 0.4242 |
| PUR | 104 | T: 71.63%, G: 28.37% | T: 28.37%, C: 71.63% | 0.1568 |
| CLM | 94 | T: 72.87%, G: 27.13% | T: 39.36%, C: 60.64% | 0.2416 |
| PEL | 85 | T: 71.76%, G: 28.24% | T: 62.94%, C: 37.06% | 0.5827 |
| EAS | 504 | T: 90.08%, G: 9.92% | T: 77.88%, C: 22.12% | 0.2141 |
| CHB | 103 | T: 87.38%, G: 12.62% | T: 72.33%, C: 27.67% | 0.2035 |
| JPT | 104 | T: 86.06%, G: 13.94% | T: 75.96%, C: 24.04% | 0.382 |
| CHS | 105 | T: 88.1%, G: 11.9% | T: 76.19%, C: 23.81% | 0.173 |
| CDX | 93 | T: 93.55%, G: 6.45% | T: 82.26%, C: 17.74% | 0.2033 |
| KHV | 99 | T: 95.96%, G: 4.04% | T: 83.33%, C: 16.67% | 0.0637 |
| EUR | 503 | T: 58.35%, G: 41.65% | T: 25.45%, C: 74.55% | 0.2346 |
| CEU | 99 | T: 63.64%, G: 36.36% | T: 27.78%, C: 72.22% | 0.2198 |
| TSI | 107 | T: 52.34%, G: 47.66% | T: 24.3%, C: 75.7% | 0.2471 |
| FIN | 99 | T: 55.05%, G: 44.95% | T: 23.74%, C: 76.26% | 0.2541 |
| GBR | 91 | T: 58.24%, G: 41.76% | T: 25.27%, C: 74.73% | 0.2425 |
| IBS | 107 | T: 62.62%, G: 37.38% | T: 26.17%, C: 73.83% | 0.2116 |
| SAS | 489 | T: 83.23%, G: 16.77% | T: 70.45%, C: 29.55% | 0.3565 |
| GIH | 103 | T: 83.5%, G: 16.5% | T: 70.39%, C: 29.61% | 0.4314 |
| PJL | 96 | T: 79.69%, G: 20.31% | T: 63.54%, C: 36.46% | 0.3754 |
| BEB | 86 | T: 86.63%, G: 13.37% | T: 71.51%, C: 28.49% | 0.3418 |
| STU | 102 | T: 83.33%, G: 16.67% | T: 74.51%, C: 25.49% | 0.3062 |
| ITU | 102 | T: 83.33%, G: 16.67% | T: 72.06%, C: 27.94% | 0.3267 |

Abbreviations: ACB, African Caribbeans in Barbados; AFR, All African; ALL, All populations; AMR, Ad Mixed American; ASW, Americans of African Ancestry in SW USA; BEB, Bengali from Bangladesh; CDX, Chinese Dai in Xishuangbanna, China; CEU, Utah Residents (CEPH) with Northern and Western European Ancestry; CHB, Han Chinese in Beijing, China; CHS, Southern Han Chinese; CLM, Colombians from Medellin, Colombia; EAS, All East Asian; ESN, Esan in Nigeria; EUR, European; FIN, Finnish in Finland; GBR, British in England and Scotland; GIH, Gujarati Indian from Houston, Texas; GWD, Gambian in Western Divisions in the Gambia; IBS, Iberian Population in Spain; ITU, Indian Telugu from the UK; JPT, Japanese in Tokyo, Japan; KHV, Kinh in Ho Chi Minh City, Vietnam; LWK, Luhya in Webuye, Kenya; MSL, Mende in Sierra Leone; MXL, Mexican Ancestry from Los Angeles USA; PEL, Peruvians from Lima, Peru; PJL, Punjabi from Lahore, Pakistan; PUR, Puerto Ricans from Puerto Rico; SAS, South Asian; STU, Sri Lankan Tamil from the UK; TSI, Toscani in Italia; YRI, Yoruba in Ibadan, Nigeria.

**S3 Table.** Pairwise linkage disequilibrium (R^2^) for rs1861759 and rs2066842 NOD2 SNPs.

| **Population** | **N** | **rs1861759 Allele Freq** | **rs2066842 Allele Freq** | **R^2^** |
| --- | --- | --- | --- | --- |
| ALL | 2504 | T: 78.39%, G: 21.61% | C: 89.8%, T: 10.2% | 0.0308 |
| AFR | 661 | T: 84.11%, G: 15.89% | C: 97.28%, T: 2.72% | 0.0036 |
| YRI | 108 | T: 84.26%, G: 15.74% | C: 99.54%, T: 0.46% | 0.0009 |
| LWK | 99 | T: 85.86%, G: 14.14% | C: 98.48%, T: 1.52% | 0.0025 |
| GWD | 113 | T: 81.86%, G: 18.14% | C: 95.13%, T: 4.87% | 0.0028 |
| MSL | 85 | T: 86.47%, G: 13.53% | C: 99.41%, T: 0.59% | 0.0009 |
| ESN | 99 | T: 85.35%, G: 14.65% | C: 99.49%, T: 0.51% | 0.0009 |
| ASW | 61 | T: 84.43%, G: 15.57% | C: 88.52%, T: 11.48% | 0.0239 |
| ACB | 96 | T: 81.25%, G: 18.75% | C: 97.4%, T: 2.6% | 0.0062 |
| AMR | 347 | T: 72.77%, G: 27.23% | C: 83.29%, T: 16.71% | 0.0751 |
| MXL | 64 | T: 75.78%, G: 24.22% | C: 85.16%, T: 14.84% | 0.0557 |
| PUR | 104 | T: 71.63%, G: 28.37% | C: 77.4%, T: 22.6% | 0.1156 |
| CLM | 94 | T: 72.87%, G: 27.13% | C: 78.72%, T: 21.28% | 0.1006 |
| PEL | 85 | T: 71.76%, G: 28.24% | C: 94.12%, T: 5.88% | 0.0246 |
| EAS | 504 | T: 90.08%, G: 9.92% | C: 99.21%, T: 0.79% | 0.0009 |
| CHB | 103 | T: 87.38%, G: 12.62% | C: 100.0%, T: 0.0% | NA |
| JPT | 104 | T: 86.06%, G: 13.94% | C: 100.0%, T: 0.0% | NA |
| CHS | 105 | T: 88.1%, G: 11.9% | C: 99.52%, T: 0.48% | 0.0006 |
| CDX | 93 | T: 93.55%, G: 6.45% | C: 98.92%, T: 1.08% | 0.0007 |
| KHV | 99 | T: 95.96%, G: 4.04% | C: 97.47%, T: 2.53% | 0.0011 |
| EUR | 503 | T: 58.35%, G: 41.65% | C: 75.35%, T: 24.65% | 0.2335 |
| CEU | 99 | T: 63.64%, G: 36.36% | C: 68.18%, T: 31.82% | 0.2667 |
| TSI | 107 | T: 52.34%, G: 47.66% | C: 76.17%, T: 23.83% | 0.2849 |
| FIN | 99 | T: 55.05%, G: 44.95% | C: 85.86%, T: 14.14% | 0.1345 |
| GBR | 91 | T: 58.24%, G: 41.76% | C: 75.27%, T: 24.73% | 0.2355 |
| IBS | 107 | T: 62.62%, G: 37.38% | C: 71.5%, T: 28.5% | 0.238 |
| SAS | 489 | T: 83.23%, G: 16.77% | C: 89.47%, T: 10.53% | 0.0237 |
| GIH | 103 | T: 83.5%, G: 16.5% | C: 92.23%, T: 7.77% | 0.0166 |
| PJL | 96 | T: 79.69%, G: 20.31% | C: 86.46%, T: 13.54% | 0.0399 |
| BEB | 86 | T: 86.63%, G: 13.37% | C: 91.28%, T: 8.72% | 0.0147 |
| STU | 102 | T: 83.33%, G: 16.67% | C: 88.73%, T: 11.27% | 0.0254 |
| ITU | 102 | T: 83.33%, G: 16.67% | C: 88.73%, T: 11.27% | 0.0254 |

Abbreviations: ACB, African Caribbeans in Barbados; AFR, All African; ALL, All populations; AMR, Ad Mixed American; ASW, Americans of African Ancestry in SW USA; BEB, Bengali from Bangladesh; CDX, Chinese Dai in Xishuangbanna, China; CEU, Utah Residents (CEPH) with Northern and Western European Ancestry; CHB, Han Chinese in Beijing, China; CHS, Southern Han Chinese; CLM, Colombians from Medellin, Colombia; EAS, All East Asian; ESN, Esan in Nigeria; EUR, European; FIN, Finnish in Finland; GBR, British in England and Scotland; GIH, Gujarati Indian from Houston, Texas; GWD, Gambian in Western Divisions in the Gambia; IBS, Iberian Population in Spain; ITU, Indian Telugu from the UK; JPT, Japanese in Tokyo, Japan; KHV, Kinh in Ho Chi Minh City, Vietnam; LWK, Luhya in Webuye, Kenya; MSL, Mende in Sierra Leone; MXL, Mexican Ancestry from Los Angeles USA; PEL, Peruvians from Lima, Peru; PJL, Punjabi from Lahore, Pakistan; PUR, Puerto Ricans from Puerto Rico; SAS, South Asian; Nan Not applicable; STU, Sri Lankan Tamil from the UK; TSI, Toscani in Italia; YRI, Yoruba in Ibadan, Nigeria.

**S4 Table.** Pairwise linkage disequilibrium (R^2^) for rs1861759 and rs2066844 NOD2 SNPs.

| **Population** | **N** | **rs1861759 Allele Freq** | **rs2066844 Allele Freq** | **R^2^** |
| --- | --- | --- | --- | --- |
| ALL | 2504 | T: 78.39%, G: 21.61% | C: 98.56%, T: 1.44% | 0.004 |
| AFR | 661 | T: 84.11%, G: 15.89% | C: 99.77%, T: 0.23% | 0.0004 |
| YRI | 108 | T: 84.26%, G: 15.74% | C: 100.0%, T: 0.0% | NA |
| LWK | 99 | T: 85.86%, G: 14.14% | C: 100.0%, T: 0.0% | NA |
| GWD | 113 | T: 81.86%, G: 18.14% | C: 100.0%, T: 0.0% | NA |
| MSL | 85 | T: 86.47%, G: 13.53% | C: 100.0%, T: 0.0% | NA |
| ESN | 99 | T: 85.35%, G: 14.65% | C: 100.0%, T: 0.0% | NA |
| ASW | 61 | T: 84.43%, G: 15.57% | C: 98.36%, T: 1.64% | 0.0031 |
| ACB | 96 | T: 81.25%, G: 18.75% | C: 99.48%, T: 0.52% | 0.0012 |
| AMR | 347 | T: 72.77%, G: 27.23% | C: 97.55%, T: 2.45% | 0.0094 |
| MXL | 64 | T: 75.78%, G: 24.22% | C: 100.0%, T: 0.0% | NA |
| PUR | 104 | T: 71.63%, G: 28.37% | C: 97.12%, T: 2.88% | 0.0118 |
| CLM | 94 | T: 72.87%, G: 27.13% | C: 94.68%, T: 5.32% | 0.0209 |
| PEL | 85 | T: 71.76%, G: 28.24% | C: 99.41%, T: 0.59% | 0.0023 |
| EAS | 504 | T: 90.08%, G: 9.92% | C: 100.0%, T: 0.0% | NA |
| CHB | 103 | T: 87.38%, G: 12.62% | C: 100.0%, T: 0.0% | NA |
| JPT | 104 | T: 86.06%, G: 13.94% | C: 100.0%, T: 0.0% | NA |
| CHS | 105 | T: 88.1%, G: 11.9% | C: 100.0%, T: 0.0% | NA |
| CDX | 93 | T: 93.55%, G: 6.45% | C: 100.0%, T: 0.0% | NA |
| KHV | 99 | T: 95.96%, G: 4.04% | C: 100.0%, T: 0.0% | NA |
| EUR | 503 | T: 58.35%, G: 41.65% | C: 94.93%, T: 5.07% | 0.0381 |
| CEU | 99 | T: 63.64%, G: 36.36% | C: 92.93%, T: 7.07% | 0.0435 |
| TSI | 107 | T: 52.34%, G: 47.66% | C: 95.33%, T: 4.67% | 0.0446 |
| FIN | 99 | T: 55.05%, G: 44.95% | C: 96.97%, T: 3.03% | 0.0255 |
| GBR | 91 | T: 58.24%, G: 41.76% | C: 95.05%, T: 4.95% | 0.0373 |
| IBS | 107 | T: 62.62%, G: 37.38% | C: 94.39%, T: 5.61% | 0.0355 |
| SAS | 489 | T: 83.23%, G: 16.77% | C: 99.9%, T: 0.1% | 0.0002 |
| GIH | 103 | T: 83.5%, G: 16.5% | C: 100.0%, T: 0.0% | NA |
| PJL | 96 | T: 79.69%, G: 20.31% | C: 100.0%, T: 0.0% | NA |
| BEB | 86 | T: 86.63%, G: 13.37% | C: 99.42%, T: 0.58% | 0.0009 |
| STU | 102 | T: 83.33%, G: 16.67% | C: 100.0%, T: 0.0% | NA |
| ITU | 102 | T: 83.33%, G: 16.67% | C: 100.0%, T: 0.0% | NA |

Abbreviations: ACB, African Caribbeans in Barbados; AFR, All African; ALL, All populations; AMR, Ad Mixed American; ASW, Americans of African Ancestry in SW USA; BEB, Bengali from Bangladesh; CDX, Chinese Dai in Xishuangbanna, China; CEU, Utah Residents (CEPH) with Northern and Western European Ancestry; CHB, Han Chinese in Beijing, China; CHS, Southern Han Chinese; CLM, Colombians from Medellin, Colombia; EAS, All East Asian; ESN, Esan in Nigeria; EUR, European; FIN, Finnish in Finland; GBR, British in England and Scotland; GIH, Gujarati Indian from Houston, Texas; GWD, Gambian in Western Divisions in the Gambia; IBS, Iberian Population in Spain; ITU, Indian Telugu from the UK; JPT, Japanese in Tokyo, Japan; KHV, Kinh in Ho Chi Minh City, Vietnam; LWK, Luhya in Webuye, Kenya; MSL, Mende in Sierra Leone; MXL, Mexican Ancestry from Los Angeles USA; PEL, Peruvians from Lima, Peru; PJL, Punjabi from Lahore, Pakistan; PUR, Puerto Ricans from Puerto Rico; SAS, South Asian; STU, Sri Lankan Tamil from the UK; TSI, Toscani in Italia; YRI, Yoruba in Ibadan, Nigeria.

**S5 Table.** Pairwise linkage disequilibrium (R^2^) for rs1861759 and rs5743278 NOD2 SNPs.

| **Population** | **N** | **rs1861759 Allele Freq** | **rs5743278 Allele Freq** | **R^2^** |
| --- | --- | --- | --- | --- |
| ALL | 2504 | T: 78.39%, G: 21.61% | C: 98.16%, G: 1.84% | 0.0052 |
| AFR | 661 | T: 84.11%, G: 15.89% | C: 93.27%, G: 6.73% | 0.0136 |
| YRI | 108 | T: 84.26%, G: 15.74% | C: 93.06%, G: 6.94% | 0.0139 |
| LWK | 99 | T: 85.86%, G: 14.14% | C: 88.89%, G: 11.11% | 0.0206 |
| GWD | 113 | T: 81.86%, G: 18.14% | C: 95.58%, G: 4.42% | 0.0103 |
| MSL | 85 | T: 86.47%, G: 13.53% | C: 93.53%, G: 6.47% | 0.0108 |
| ESN | 99 | T: 85.35%, G: 14.65% | C: 93.43%, G: 6.57% | 0.0121 |
| ASW | 61 | T: 84.43%, G: 15.57% | C: 93.44%, G: 6.56% | 0.0129 |
| ACB | 96 | T: 81.25%, G: 18.75% | C: 94.79%, G: 5.21% | 0.0127 |
| AMR | 347 | T: 72.77%, G: 27.23% | C: 99.57%, G: 0.43% | 0.0016 |
| MXL | 64 | T: 75.78%, G: 24.22% | C: 100.0%, G: 0.0% | NA |
| PUR | 104 | T: 71.63%, G: 28.37% | C: 99.52%, G: 0.48% | 0.0019 |
| CLM | 94 | T: 72.87%, G: 27.13% | C: 99.47%, G: 0.53% | 0.002 |
| PEL | 85 | T: 71.76%, G: 28.24% | C: 99.41%, G: 0.59% | 0.0023 |
| EAS | 504 | T: 90.08%, G: 9.92% | C: 100.0%, G: 0.0% | NA |
| CHB | 103 | T: 87.38%, G: 12.62% | C: 100.0%, G: 0.0% | NA |
| JPT | 104 | T: 86.06%, G: 13.94% | C: 100.0%, G: 0.0% | NA |
| CHS | 105 | T: 88.1%, G: 11.9% | C: 100.0%, G: 0.0% | NA |
| CDX | 93 | T: 93.55%, G: 6.45% | C: 100.0%, G: 0.0% | NA |
| KHV | 99 | T: 95.96%, G: 4.04% | C: 100.0%, G: 0.0% | NA |
| EUR | 503 | T: 58.35%, G: 41.65% | C: 100.0%, G: 0.0% | NA |
| CEU | 99 | T: 63.64%, G: 36.36% | C: 100.0%, G: 0.0% | NA |
| TSI | 107 | T: 52.34%, G: 47.66% | C: 100.0%, G: 0.0% | NA |
| FIN | 99 | T: 55.05%, G: 44.95% | C: 100.0%, G: 0.0% | NA |
| GBR | 91 | T: 58.24%, G: 41.76% | C: 100.0%, G: 0.0% | NA |
| IBS | 107 | T: 62.62%, G: 37.38% | C: 100.0%, G: 0.0% | NA |
| SAS | 489 | T: 83.23%, G: 16.77% | C: 100.0%, G: 0.0% | NA |
| GIH | 103 | T: 83.5%, G: 16.5% | C: 100.0%, G: 0.0% | NA |
| PJL | 96 | T: 79.69%, G: 20.31% | C: 100.0%, G: 0.0% | NA |
| BEB | 86 | T: 86.63%, G: 13.37% | C: 100.0%, G: 0.0% | NA |
| STU | 102 | T: 83.33%, G: 16.67% | C: 100.0%, G: 0.0% | NA |
| ITU | 102 | T: 83.33%, G: 16.67% | C: 100.0%, G: 0.0% | NA |

Abbreviations: ACB, African Caribbeans in Barbados; AFR, All African; ALL, All populations; AMR, Ad Mixed American; ASW, Americans of African Ancestry in SW USA; BEB, Bengali from Bangladesh; CDX, Chinese Dai in Xishuangbanna, China; CEU, Utah Residents (CEPH) with Northern and Western European Ancestry; CHB, Han Chinese in Beijing, China; CHS, Southern Han Chinese; CLM, Colombians from Medellin, Colombia; EAS, All East Asian; ESN, Esan in Nigeria; EUR, European; FIN, Finnish in Finland; GBR, British in England and Scotland; GIH, Gujarati Indian from Houston, Texas; GWD, Gambian in Western Divisions in the Gambia; IBS, Iberian Population in Spain; ITU, Indian Telugu from the UK; JPT, Japanese in Tokyo, Japan; KHV, Kinh in Ho Chi Minh City, Vietnam; LWK, Luhya in Webuye, Kenya; MSL, Mende in Sierra Leone; MXL, Mexican Ancestry from Los Angeles USA; PEL, Peruvians from Lima, Peru; PJL, Punjabi from Lahore, Pakistan; PUR, Puerto Ricans from Puerto Rico; SAS, South Asian; STU, Sri Lankan Tamil from the UK; TSI, Toscani in Italia; YRI, Yoruba in Ibadan, Nigeria.

**S6 Table.** Pairwise linkage disequilibrium (R^2^) for rs1861759 and rs6500328 NOD2 SNPs.

| **Population** | **N** | **rs1861759 Allele Freq** | **rs6500328 Allele Freq** | **R^2^** |
| --- | --- | --- | --- | --- |
| ALL | 2504 | T: 78.39%, G: 21.61% | A: 67.25%, G: 32.75% | 0.5582 |
| AFR | 661 | T: 84.11%, G: 15.89% | A: 52.95%, G: 47.05% | 0.205 |
| YRI | 108 | T: 84.26%, G: 15.74% | A: 53.24%, G: 46.76% | 0.2127 |
| LWK | 99 | T: 85.86%, G: 14.14% | A: 46.46%, G: 53.54% | 0.1218 |
| GWD | 113 | T: 81.86%, G: 18.14% | A: 57.52%, G: 42.48% | 0.2752 |
| MSL | 85 | T: 86.47%, G: 13.53% | A: 53.53%, G: 46.47% | 0.1802 |
| ESN | 99 | T: 85.35%, G: 14.65% | A: 50.51%, G: 49.49% | 0.1751 |
| ASW | 61 | T: 84.43%, G: 15.57% | A: 60.66%, G: 39.34% | 0.2844 |
| ACB | 96 | T: 81.25%, G: 18.75% | A: 51.04%, G: 48.96% | 0.2406 |
| AMR | 347 | T: 72.77%, G: 27.23% | A: 67.72%, G: 32.28% | 0.7853 |
| MXL | 64 | T: 75.78%, G: 24.22% | A: 74.22%, G: 25.78% | 0.92 |
| PUR | 104 | T: 71.63%, G: 28.37% | A: 64.42%, G: 35.58% | 0.717 |
| CLM | 94 | T: 72.87%, G: 27.13% | A: 68.62%, G: 31.38% | 0.8139 |
| PEL | 85 | T: 71.76%, G: 28.24% | A: 65.88%, G: 34.12% | 0.7598 |
| EAS | 504 | T: 90.08%, G: 9.92% | A: 82.34%, G: 17.66% | 0.5135 |
| CHB | 103 | T: 87.38%, G: 12.62% | A: 75.24%, G: 24.76% | 0.439 |
| JPT | 104 | T: 86.06%, G: 13.94% | A: 79.33%, G: 20.67% | 0.6217 |
| CHS | 105 | T: 88.1%, G: 11.9% | A: 80.95%, G: 19.05% | 0.5743 |
| CDX | 93 | T: 93.55%, G: 6.45% | A: 86.02%, G: 13.98% | 0.4244 |
| KHV | 99 | T: 95.96%, G: 4.04% | A: 90.91%, G: 9.09% | 0.4211 |
| EUR | 503 | T: 58.35%, G: 41.65% | A: 57.06%, G: 42.94% | 0.9405 |
| CEU | 99 | T: 63.64%, G: 36.36% | A: 63.64%, G: 36.36% | 1 |
| TSI | 107 | T: 52.34%, G: 47.66% | A: 50.93%, G: 49.07% | 0.9094 |
| FIN | 99 | T: 55.05%, G: 44.95% | A: 51.52%, G: 48.48% | 0.8675 |
| GBR | 91 | T: 58.24%, G: 41.76% | A: 58.24%, G: 41.76% | 1 |
| IBS | 107 | T: 62.62%, G: 37.38% | A: 61.21%, G: 38.79% | 0.9423 |
| SAS | 489 | T: 83.23%, G: 16.77% | A: 81.19%, G: 18.81% | 0.8435 |
| GIH | 103 | T: 83.5%, G: 16.5% | A: 81.07%, G: 18.93% | 0.8465 |
| PJL | 96 | T: 79.69%, G: 20.31% | A: 79.17%, G: 20.83% | 0.9686 |
| BEB | 86 | T: 86.63%, G: 13.37% | A: 83.14%, G: 16.86% | 0.7612 |
| STU | 102 | T: 83.33%, G: 16.67% | A: 81.86%, G: 18.14% | 0.839 |
| ITU | 102 | T: 83.33%, G: 16.67% | A: 80.88%, G: 19.12% | 0.7857 |

Abbreviations: ACB, African Caribbeans in Barbados; AFR, All African; ALL, All populations; AMR, Ad Mixed American; ASW, Americans of African Ancestry in SW USA; BEB, Bengali from Bangladesh; CDX, Chinese Dai in Xishuangbanna, China; CEU, Utah Residents (CEPH) with Northern and Western European Ancestry; CHB, Han Chinese in Beijing, China; CHS, Southern Han Chinese; CLM, Colombians from Medellin, Colombia; EAS, All East Asian; ESN, Esan in Nigeria; EUR, European; FIN, Finnish in Finland; GBR, British in England and Scotland; GIH, Gujarati Indian from Houston, Texas; GWD, Gambian in Western Divisions in the Gambia; IBS, Iberian Population in Spain; ITU, Indian Telugu from the UK; JPT, Japanese in Tokyo, Japan; KHV, Kinh in Ho Chi Minh City, Vietnam; LWK, Luhya in Webuye, Kenya; MSL, Mende in Sierra Leone; MXL, Mexican Ancestry from Los Angeles USA; PEL, Peruvians from Lima, Peru; PJL, Punjabi from Lahore, Pakistan; PUR, Puerto Ricans from Puerto Rico; SAS, South Asian; STU, Sri Lankan Tamil from the UK; TSI, Toscani in Italia; YRI, Yoruba in Ibadan, Nigeria.

**S7 Table.** Pairwise linkage disequilibrium (R^2^) for rs1861759 and rs2111234 NOD2 SNPs.

| **Population** | **N** | **rs1861759 Allele Freq** | **rs2111234 Allele Freq** | **R^2^** |
| --- | --- | --- | --- | --- |
| ALL | 2504 | T: 78.39%, G: 21.61% | G: 48.7%, A: 51.3% | 0.2479 |
| AFR | 661 | T: 84.11%, G: 15.89% | G: 33.96%, A: 66.04% | 0.0971 |
| YRI | 108 | T: 84.26%, G: 15.74% | G: 35.65%, A: 64.35% | 0.1035 |
| LWK | 99 | T: 85.86%, G: 14.14% | G: 37.37%, A: 62.63% | 0.0983 |
| GWD | 113 | T: 81.86%, G: 18.14% | G: 29.65%, A: 70.35% | 0.0934 |
| MSL | 85 | T: 86.47%, G: 13.53% | G: 36.47%, A: 63.53% | 0.0898 |
| ESN | 99 | T: 85.35%, G: 14.65% | G: 31.82%, A: 68.18% | 0.0801 |
| ASW | 61 | T: 84.43%, G: 15.57% | G: 34.43%, A: 65.57% | 0.0968 |
| ACB | 96 | T: 81.25%, G: 18.75% | G: 33.33%, A: 66.67% | 0.1154 |
| AMR | 347 | T: 72.77%, G: 27.23% | G: 48.41%, A: 51.59% | 0.3436 |
| MXL | 64 | T: 75.78%, G: 24.22% | G: 58.59%, A: 41.41% | 0.4522 |
| PUR | 104 | T: 71.63%, G: 28.37% | G: 36.54%, A: 63.46% | 0.228 |
| CLM | 94 | T: 72.87%, G: 27.13% | G: 42.02%, A: 57.98% | 0.2698 |
| PEL | 85 | T: 71.76%, G: 28.24% | G: 62.35%, A: 37.65% | 0.6088 |
| EAS | 504 | T: 90.08%, G: 9.92% | G: 76.49%, A: 23.51% | 0.3043 |
| CHB | 103 | T: 87.38%, G: 12.62% | G: 72.33%, A: 27.67% | 0.3015 |
| JPT | 104 | T: 86.06%, G: 13.94% | G: 73.08%, A: 26.92% | 0.3241 |
| CHS | 105 | T: 88.1%, G: 11.9% | G: 78.57%, A: 21.43% | 0.4463 |
| CDX | 93 | T: 93.55%, G: 6.45% | G: 76.88%, A: 23.12% | 0.2294 |
| KHV | 99 | T: 95.96%, G: 4.04% | G: 81.82%, A: 18.18% | 0.1895 |
| EUR | 503 | T: 58.35%, G: 41.65% | G: 29.22%, A: 70.78% | 0.2852 |
| CEU | 99 | T: 63.64%, G: 36.36% | G: 28.79%, A: 71.21% | 0.231 |
| TSI | 107 | T: 52.34%, G: 47.66% | G: 26.17%, A: 73.83% | 0.2991 |
| FIN | 99 | T: 55.05%, G: 44.95% | G: 31.31%, A: 68.69% | 0.3722 |
| GBR | 91 | T: 58.24%, G: 41.76% | G: 29.12%, A: 70.88% | 0.2946 |
| IBS | 107 | T: 62.62%, G: 37.38% | G: 30.84%, A: 69.16% | 0.2451 |
| SAS | 489 | T: 83.23%, G: 16.77% | G: 60.22%, A: 39.78% | 0.275 |
| GIH | 103 | T: 83.5%, G: 16.5% | G: 60.68%, A: 39.32% | 0.3051 |
| PJL | 96 | T: 79.69%, G: 20.31% | G: 55.73%, A: 44.27% | 0.292 |
| BEB | 86 | T: 86.63%, G: 13.37% | G: 62.79%, A: 37.21% | 0.2605 |
| STU | 102 | T: 83.33%, G: 16.67% | G: 60.78%, A: 39.22% | 0.2529 |
| ITU | 102 | T: 83.33%, G: 16.67% | G: 61.27%, A: 38.73% | 0.2586 |

Abbreviations: ACB, African Caribbeans in Barbados; AFR, All African; ALL, All populations; AMR, Ad Mixed American; ASW, Americans of African Ancestry in SW USA; BEB, Bengali from Bangladesh; CDX, Chinese Dai in Xishuangbanna, China; CEU, Utah Residents (CEPH) with Northern and Western European Ancestry; CHB, Han Chinese in Beijing, China; CHS, Southern Han Chinese; CLM, Colombians from Medellin, Colombia; EAS, All East Asian; ESN, Esan in Nigeria; EUR, European; FIN, Finnish in Finland; GBR, British in England and Scotland; GIH, Gujarati Indian from Houston, Texas; GWD, Gambian in Western Divisions in the Gambia; IBS, Iberian Population in Spain; ITU, Indian Telugu from the UK; JPT, Japanese in Tokyo, Japan; KHV, Kinh in Ho Chi Minh City, Vietnam; LWK, Luhya in Webuye, Kenya; MSL, Mende in Sierra Leone; MXL, Mexican Ancestry from Los Angeles USA; PEL, Peruvians from Lima, Peru; PJL, Punjabi from Lahore, Pakistan; PUR, Puerto Ricans from Puerto Rico; SAS, South Asian; STU, Sri Lankan Tamil from the UK; TSI, Toscani in Italia; YRI, Yoruba in Ibadan, Nigeria.

**S8 Table.** Pairwise linkage disequilibrium (R^2^) for rs7194886 and rs9302752 NOD2 SNPs.

| **Population** | **N** | **rs7194886 Allele Freq** | **rs9302752 Allele Freq** | **R^2^** |
| --- | --- | --- | --- | --- |
| ALL | 2504 | C: 74.36%, T: 25.64% | T: 50.02%, C: 49.98% | 0.3302 |
| AFR | 661 | C: 74.21%, T: 25.79% | T: 34.95%, C: 65.05% | 0.1836 |
| YRI | 108 | C: 77.31%, T: 22.69% | T: 39.81%, C: 60.19% | 0.1941 |
| LWK | 99 | C: 77.78%, T: 22.22% | T: 34.85%, C: 65.15% | 0.1528 |
| GWD | 113 | C: 72.12%, T: 27.88% | T: 31.86%, C: 68.14% | 0.1807 |
| MSL | 85 | C: 78.24%, T: 21.76% | T: 41.76%, C: 58.24% | 0.1745 |
| ESN | 99 | C: 72.73%, T: 27.27% | T: 30.3%, C: 69.7% | 0.163 |
| ASW | 61 | C: 72.95%, T: 27.05% | T: 35.25%, C: 64.75% | 0.2018 |
| ACB | 96 | C: 68.23%, T: 31.77% | T: 31.77%, C: 68.23% | 0.2168 |
| AMR | 347 | C: 69.88%, T: 30.12% | T: 45.1%, C: 54.9% | 0.354 |
| MXL | 64 | C: 76.56%, T: 23.44% | T: 57.03%, C: 42.97% | 0.4063 |
| PUR | 104 | C: 63.46%, T: 36.54% | T: 28.37%, C: 71.63% | 0.228 |
| CLM | 94 | C: 68.62%, T: 31.38% | T: 39.36%, C: 60.64% | 0.2969 |
| PEL | 85 | C: 74.12%, T: 25.88% | T: 62.94%, C: 37.06% | 0.5931 |
| EAS | 504 | C: 90.48%, T: 9.52% | T: 77.88%, C: 22.12% | 0.351 |
| CHB | 103 | C: 89.81%, T: 10.19% | T: 72.33%, C: 27.67% | 0.2589 |
| JPT | 104 | C: 90.87%, T: 9.13% | T: 75.96%, C: 24.04% | 0.3177 |
| CHS | 105 | C: 88.57%, T: 11.43% | T: 76.19%, C: 23.81% | 0.4129 |
| CDX | 93 | C: 91.4%, T: 8.6% | T: 82.26%, C: 17.74% | 0.4364 |
| KHV | 99 | C: 91.92%, T: 8.08% | T: 83.33%, C: 16.67% | 0.3761 |
| EUR | 503 | C: 55.57%, T: 44.43% | T: 25.45%, C: 74.55% | 0.2729 |
| CEU | 99 | C: 63.64%, T: 36.36% | T: 27.78%, C: 72.22% | 0.2198 |
| TSI | 107 | C: 52.34%, T: 47.66% | T: 24.3%, C: 75.7% | 0.2923 |
| FIN | 99 | C: 46.97%, T: 53.03% | T: 23.74%, C: 76.26% | 0.3514 |
| GBR | 91 | C: 56.04%, T: 43.96% | T: 25.27%, C: 74.73% | 0.2653 |
| IBS | 107 | C: 58.88%, T: 41.12% | T: 26.17%, C: 73.83% | 0.2475 |
| SAS | 489 | C: 80.47%, T: 19.53% | T: 70.45%, C: 29.55% | 0.4879 |
| GIH | 103 | C: 78.16%, T: 21.84% | T: 70.39%, C: 29.61% | 0.6231 |
| PJL | 96 | C: 78.12%, T: 21.88% | T: 63.54%, C: 36.46% | 0.4176 |
| BEB | 86 | C: 83.14%, T: 16.86% | T: 71.51%, C: 28.49% | 0.4156 |
| STU | 102 | C: 81.37%, T: 18.63% | T: 74.51%, C: 25.49% | 0.4934 |
| ITU | 102 | C: 81.86%, T: 18.14% | T: 72.06%, C: 27.94% | 0.4889 |

Abbreviations: ACB, African Caribbeans in Barbados; AFR, All African; ALL, All populations; AMR, Ad Mixed American; ASW, Americans of African Ancestry in SW USA; BEB, Bengali from Bangladesh; CDX, Chinese Dai in Xishuangbanna, China; CEU, Utah Residents (CEPH) with Northern and Western European Ancestry; CHB, Han Chinese in Beijing, China; CHS, Southern Han Chinese; CLM, Colombians from Medellin, Colombia; EAS, All East Asian; ESN, Esan in Nigeria; EUR, European; FIN, Finnish in Finland; GBR, British in England and Scotland; GIH, Gujarati Indian from Houston, Texas; GWD, Gambian in Western Divisions in the Gambia; IBS, Iberian Population in Spain; ITU, Indian Telugu from the UK; JPT, Japanese in Tokyo, Japan; KHV, Kinh in Ho Chi Minh City, Vietnam; LWK, Luhya in Webuye, Kenya; MSL, Mende in Sierra Leone; MXL, Mexican Ancestry from Los Angeles USA; PEL, Peruvians from Lima, Peru; PJL, Punjabi from Lahore, Pakistan; PUR, Puerto Ricans from Puerto Rico; SAS, South Asian; STU, Sri Lankan Tamil from the UK; TSI, Toscani in Italia; YRI, Yoruba in Ibadan, Nigeria.

**S9 Table.** Pairwise linkage disequilibrium (R^2^) for rs7194886 and rs2066842 NOD2 SNPs.

| **Population** | **N** | **rs7194886 Allele Freq** | **rs2066842 Allele Freq** | **R^2^** |
| --- | --- | --- | --- | --- |
| ALL | 2504 | C: 74.36%, T: 25.64% | C: 89.8%, T: 10.2% | 0.0297 |
| AFR | 661 | C: 74.21%, T: 25.79% | C: 97.28%, T: 2.72% | 0.006 |
| YRI | 108 | C: 77.31%, T: 22.69% | C: 99.54%, T: 0.46% | 0.0014 |
| LWK | 99 | C: 77.78%, T: 22.22% | C: 98.48%, T: 1.52% | 0.0044 |
| GWD | 113 | C: 72.12%, T: 27.88% | C: 95.13%, T: 4.87% | 0.0198 |
| MSL | 85 | C: 78.24%, T: 21.76% | C: 99.41%, T: 0.59% | 0.0016 |
| ESN | 99 | C: 72.73%, T: 27.27% | C: 99.49%, T: 0.51% | 0.0019 |
| ASW | 61 | C: 72.95%, T: 27.05% | C: 88.52%, T: 11.48% | 0.0107 |
| ACB | 96 | C: 68.23%, T: 31.77% | C: 97.4%, T: 2.6% | 0.0125 |
| AMR | 347 | C: 69.88%, T: 30.12% | C: 83.29%, T: 16.71% | 0.0593 |
| MXL | 64 | C: 76.56%, T: 23.44% | C: 85.16%, T: 14.84% | 0.0321 |
| PUR | 104 | C: 63.46%, T: 36.54% | C: 77.4%, T: 22.6% | 0.1491 |
| CLM | 94 | C: 68.62%, T: 31.38% | C: 78.72%, T: 21.28% | 0.1047 |
| PEL | 85 | C: 74.12%, T: 25.88% | C: 94.12%, T: 5.88% | 0.0006 |
| EAS | 504 | C: 90.48%, T: 9.52% | C: 99.21%, T: 0.79% | 0.0008 |
| CHB | 103 | C: 89.81%, T: 10.19% | C: 100.0%, T: 0.0% | NA |
| JPT | 104 | C: 90.87%, T: 9.13% | C: 100.0%, T: 0.0% | NA |
| CHS | 105 | C: 88.57%, T: 11.43% | C: 99.52%, T: 0.48% | 0.0006 |
| CDX | 93 | C: 91.4%, T: 8.6% | C: 98.92%, T: 1.08% | 0.001 |
| KHV | 99 | C: 91.92%, T: 8.08% | C: 97.47%, T: 2.53% | 0.0023 |
| EUR | 503 | C: 55.57%, T: 44.43% | C: 75.35%, T: 24.65% | 0.225 |
| CEU | 99 | C: 63.64%, T: 36.36% | C: 68.18%, T: 31.82% | 0.2439 |
| TSI | 107 | C: 52.34%, T: 47.66% | C: 76.17%, T: 23.83% | 0.24 |
| FIN | 99 | C: 46.97%, T: 53.03% | C: 85.86%, T: 14.14% | 0.1618 |
| GBR | 91 | C: 56.04%, T: 43.96% | C: 75.27%, T: 24.73% | 0.2576 |
| IBS | 107 | C: 58.88%, T: 41.12% | C: 71.5%, T: 28.5% | 0.1967 |
| SAS | 489 | C: 80.47%, T: 19.53% | C: 89.47%, T: 10.53% | 0.0258 |
| GIH | 103 | C: 78.16%, T: 21.84% | C: 92.23%, T: 7.77% | 0.0235 |
| PJL | 96 | C: 78.12%, T: 21.88% | C: 86.46%, T: 13.54% | 0.0439 |
| BEB | 86 | C: 83.14%, T: 16.86% | C: 91.28%, T: 8.72% | 0.0194 |
| STU | 102 | C: 81.37%, T: 18.63% | C: 88.73%, T: 11.27% | 0.0291 |
| ITU | 102 | C: 81.86%, T: 18.14% | C: 88.73%, T: 11.27% | 0.0163 |

Abbreviations: ACB, African Caribbeans in Barbados; AFR, All African; ALL, All populations; AMR, Ad Mixed American; ASW, Americans of African Ancestry in SW USA; BEB, Bengali from Bangladesh; CDX, Chinese Dai in Xishuangbanna, China; CEU, Utah Residents (CEPH) with Northern and Western European Ancestry; CHB, Han Chinese in Beijing, China; CHS, Southern Han Chinese; CLM, Colombians from Medellin, Colombia; EAS, All East Asian; ESN, Esan in Nigeria; EUR, European; FIN, Finnish in Finland; GBR, British in England and Scotland; GIH, Gujarati Indian from Houston, Texas; GWD, Gambian in Western Divisions in the Gambia; IBS, Iberian Population in Spain; ITU, Indian Telugu from the UK; JPT, Japanese in Tokyo, Japan; KHV, Kinh in Ho Chi Minh City, Vietnam; LWK, Luhya in Webuye, Kenya; MSL, Mende in Sierra Leone; MXL, Mexican Ancestry from Los Angeles USA; PEL, Peruvians from Lima, Peru; PJL, Punjabi from Lahore, Pakistan; PUR, Puerto Ricans from Puerto Rico; SAS, South Asian; STU, Sri Lankan Tamil from the UK; TSI, Toscani in Italia; YRI, Yoruba in Ibadan, Nigeria.

**S10 Table.** Pairwise linkage disequilibrium (R^2^) for rs7194886 and rs2066844 NOD2 SNPs.

| **Population** | **N** | **rs7194886 Allele Freq** | **rs2066844 Allele Freq** | **R^2^** |
| --- | --- | --- | --- | --- |
| ALL | 2504 | C: 74.36%, T: 25.64% | C: 98.56%, T: 1.44% | 0.0035 |
| AFR | 661 | C: 74.21%, T: 25.79% | C: 99.77%, T: 0.23% | 0.0008 |
| YRI | 108 | C: 77.31%, T: 22.69% | C: 100.0%, T: 0.0% | NA |
| LWK | 99 | C: 77.78%, T: 22.22% | C: 100.0%, T: 0.0% | NA |
| GWD | 113 | C: 72.12%, T: 27.88% | C: 100.0%, T: 0.0% | NA |
| MSL | 85 | C: 78.24%, T: 21.76% | C: 100.0%, T: 0.0% | NA |
| ESN | 99 | C: 72.73%, T: 27.27% | C: 100.0%, T: 0.0% | NA |
| ASW | 61 | C: 72.95%, T: 27.05% | C: 98.36%, T: 1.64% | 0.0062 |
| ACB | 96 | C: 68.23%, T: 31.77% | C: 99.48%, T: 0.52% | 0.0024 |
| AMR | 347 | C: 69.88%, T: 30.12% | C: 97.55%, T: 2.45% | 0.0108 |
| MXL | 64 | C: 76.56%, T: 23.44% | C: 100.0%, T: 0.0% | NA |
| PUR | 104 | C: 63.46%, T: 36.54% | C: 97.12%, T: 2.88% | 0.0171 |
| CLM | 94 | C: 68.62%, T: 31.38% | C: 94.68%, T: 5.32% | 0.0257 |
| PEL | 85 | C: 74.12%, T: 25.88% | C: 99.41%, T: 0.59% | 0.0021 |
| EAS | 504 | C: 90.48%, T: 9.52% | C: 100.0%, T: 0.0% | NA |
| CHB | 103 | C: 89.81%, T: 10.19% | C: 100.0%, T: 0.0% | NA |
| JPT | 104 | C: 90.87%, T: 9.13% | C: 100.0%, T: 0.0% | NA |
| CHS | 105 | C: 88.57%, T: 11.43% | C: 100.0%, T: 0.0% | NA |
| CDX | 93 | C: 91.4%, T: 8.6% | C: 100.0%, T: 0.0% | NA |
| KHV | 99 | C: 91.92%, T: 8.08% | C: 100.0%, T: 0.0% | NA |
| EUR | 503 | C: 55.57%, T: 44.43% | C: 94.93%, T: 5.07% | 0.0321 |
| CEU | 99 | C: 63.64%, T: 36.36% | C: 92.93%, T: 7.07% | 0.0435 |
| TSI | 107 | C: 52.34%, T: 47.66% | C: 95.33%, T: 4.67% | 0.0279 |
| FIN | 99 | C: 46.97%, T: 53.03% | C: 96.97%, T: 3.03% | 0.0353 |
| GBR | 91 | C: 56.04%, T: 43.96% | C: 95.05%, T: 4.95% | 0.0408 |
| IBS | 107 | C: 58.88%, T: 41.12% | C: 94.39%, T: 5.61% | 0.0147 |
| SAS | 489 | C: 80.47%, T: 19.53% | C: 99.9%, T: 0.1% | 0.0002 |
| GIH | 103 | C: 78.16%, T: 21.84% | C: 100.0%, T: 0.0% | NA |
| PJL | 96 | C: 78.12%, T: 21.88% | C: 100.0%, T: 0.0% | NA |
| BEB | 86 | C: 83.14%, T: 16.86% | C: 99.42%, T: 0.58% | 0.0012 |
| STU | 102 | C: 81.37%, T: 18.63% | C: 100.0%, T: 0.0% | NA |
| ITU | 102 | C: 81.86%, T: 18.14% | C: 100.0%, T: 0.0% | NA |

Abbreviations: ACB, African Caribbeans in Barbados; AFR, All African; ALL, All populations; AMR, Ad Mixed American; ASW, Americans of African Ancestry in SW USA; BEB, Bengali from Bangladesh; CDX, Chinese Dai in Xishuangbanna, China; CEU, Utah Residents (CEPH) with Northern and Western European Ancestry; CHB, Han Chinese in Beijing, China; CHS, Southern Han Chinese; CLM, Colombians from Medellin, Colombia; EAS, All East Asian; ESN, Esan in Nigeria; EUR, European; FIN, Finnish in Finland; GBR, British in England and Scotland; GIH, Gujarati Indian from Houston, Texas; GWD, Gambian in Western Divisions in the Gambia; IBS, Iberian Population in Spain; ITU, Indian Telugu from the UK; JPT, Japanese in Tokyo, Japan; KHV, Kinh in Ho Chi Minh City, Vietnam; LWK, Luhya in Webuye, Kenya; MSL, Mende in Sierra Leone; MXL, Mexican Ancestry from Los Angeles USA; PEL, Peruvians from Lima, Peru; PJL, Punjabi from Lahore, Pakistan; PUR, Puerto Ricans from Puerto Rico; SAS, South Asian; STU, Sri Lankan Tamil from the UK; TSI, Toscani in Italia; YRI, Yoruba in Ibadan, Nigeria.

**S11 Table.** Pairwise linkage disequilibrium (R^2^) for rs7194886 and rs5743278 NOD2 SNPs.

| **Population** | **N** | **rs7194886 Allele Freq** | **rs5743278 Allele Freq** | **R^2^** |
| --- | --- | --- | --- | --- |
| ALL | 2504 | C: 74.36%, T: 25.64% | C: 98.16%, G: 1.84% | 0.0044 |
| AFR | 661 | C: 74.21%, T: 25.79% | C: 93.27%, G: 6.73% | 0.0171 |
| YRI | 108 | C: 77.31%, T: 22.69% | C: 93.06%, G: 6.94% | 0.0219 |
| LWK | 99 | C: 77.78%, T: 22.22% | C: 88.89%, G: 11.11% | 0.0125 |
| GWD | 113 | C: 72.12%, T: 27.88% | C: 95.58%, G: 4.42% | 0.0179 |
| MSL | 85 | C: 78.24%, T: 21.76% | C: 93.53%, G: 6.47% | 0.0192 |
| ESN | 99 | C: 72.73%, T: 27.27% | C: 93.43%, G: 6.57% | 0.0136 |
| ASW | 61 | C: 72.95%, T: 27.05% | C: 93.44%, G: 6.56% | 0.0075 |
| ACB | 96 | C: 68.23%, T: 31.77% | C: 94.79%, G: 5.21% | 0.0256 |
| AMR | 347 | C: 69.88%, T: 30.12% | C: 99.57%, G: 0.43% | 0.0019 |
| MXL | 64 | C: 76.56%, T: 23.44% | C: 100.0%, G: 0.0% | NA |
| PUR | 104 | C: 63.46%, T: 36.54% | C: 99.52%, G: 0.48% | 0.0028 |
| CLM | 94 | C: 68.62%, T: 31.38% | C: 99.47%, G: 0.53% | 0.0024 |
| PEL | 85 | C: 74.12%, T: 25.88% | C: 99.41%, G: 0.59% | 0.0021 |
| EAS | 504 | C: 90.48%, T: 9.52% | C: 100.0%, G: 0.0% | NA |
| CHB | 103 | C: 89.81%, T: 10.19% | C: 100.0%, G: 0.0% | NA |
| JPT | 104 | C: 90.87%, T: 9.13% | C: 100.0%, G: 0.0% | NA |
| CHS | 105 | C: 88.57%, T: 11.43% | C: 100.0%, G: 0.0% | NA |
| CDX | 93 | C: 91.4%, T: 8.6% | C: 100.0%, G: 0.0% | NA |
| KHV | 99 | C: 91.92%, T: 8.08% | C: 100.0%, G: 0.0% | NA |
| EUR | 503 | C: 55.57%, T: 44.43% | C: 100.0%, G: 0.0% | NA |
| CEU | 99 | C: 63.64%, T: 36.36% | C: 100.0%, G: 0.0% | NA |
| TSI | 107 | C: 52.34%, T: 47.66% | C: 100.0%, G: 0.0% | NA |
| FIN | 99 | C: 46.97%, T: 53.03% | C: 100.0%, G: 0.0% | NA |
| GBR | 91 | C: 56.04%, T: 43.96% | C: 100.0%, G: 0.0% | NA |
| IBS | 107 | C: 58.88%, T: 41.12% | C: 100.0%, G: 0.0% | NA |
| SAS | 489 | C: 80.47%, T: 19.53% | C: 100.0%, G: 0.0% | NA |
| GIH | 103 | C: 78.16%, T: 21.84% | C: 100.0%, G: 0.0% | NA |
| PJL | 96 | C: 78.12%, T: 21.88% | C: 100.0%, G: 0.0% | NA |
| BEB | 86 | C: 83.14%, T: 16.86% | C: 100.0%, G: 0.0% | NA |
| STU | 102 | C: 81.37%, T: 18.63% | C: 100.0%, G: 0.0% | NA |
| ITU | 102 | C: 81.86%, T: 18.14% | C: 100.0%, G: 0.0% | NA |

Abbreviations: ACB, African Caribbeans in Barbados; AFR, All African; ALL, All populations; AMR, Ad Mixed American; ASW, Americans of African Ancestry in SW USA; BEB, Bengali from Bangladesh; CDX, Chinese Dai in Xishuangbanna, China; CEU, Utah Residents (CEPH) with Northern and Western European Ancestry; CHB, Han Chinese in Beijing, China; CHS, Southern Han Chinese; CLM, Colombians from Medellin, Colombia; EAS, All East Asian; ESN, Esan in Nigeria; EUR, European; FIN, Finnish in Finland; GBR, British in England and Scotland; GIH, Gujarati Indian from Houston, Texas; GWD, Gambian in Western Divisions in the Gambia; IBS, Iberian Population in Spain; ITU, Indian Telugu from the UK; JPT, Japanese in Tokyo, Japan; KHV, Kinh in Ho Chi Minh City, Vietnam; LWK, Luhya in Webuye, Kenya; MSL, Mende in Sierra Leone; MXL, Mexican Ancestry from Los Angeles USA; PEL, Peruvians from Lima, Peru; PJL, Punjabi from Lahore, Pakistan; PUR, Puerto Ricans from Puerto Rico; SAS, South Asian; STU, Sri Lankan Tamil from the UK; TSI, Toscani in Italia; YRI, Yoruba in Ibadan, Nigeria.

**S12 Table.** Pairwise linkage disequilibrium (R^2^) for rs7194886 and rs6500328 NOD2 SNPs.

| **Population** | **N** | **rs7194886 Allele Freq** | **rs6500328 Allele Freq** | **R^2^** |
| --- | --- | --- | --- | --- |
| ALL | 2504 | C: 74.36%, T: 25.64% | A: 67.25%, G: 32.75% | 0.293 |
| AFR | 661 | C: 74.21%, T: 25.79% | A: 52.95%, G: 47.05% | 0.0197 |
| YRI | 108 | C: 77.31%, T: 22.69% | A: 53.24%, G: 46.76% | 0.0047 |
| LWK | 99 | C: 77.78%, T: 22.22% | A: 46.46%, G: 53.54% | 0.0919 |
| GWD | 113 | C: 72.12%, T: 27.88% | A: 57.52%, G: 42.48% | 0.0042 |
| MSL | 85 | C: 78.24%, T: 21.76% | A: 53.53%, G: 46.47% | 0.0118 |
| ESN | 99 | C: 72.73%, T: 27.27% | A: 50.51%, G: 49.49% | 0.0094 |
| ASW | 61 | C: 72.95%, T: 27.05% | A: 60.66%, G: 39.34% | 0.013 |
| ACB | 96 | C: 68.23%, T: 31.77% | A: 51.04%, G: 48.96% | 0.0621 |
| AMR | 347 | C: 69.88%, T: 30.12% | A: 67.72%, G: 32.28% | 0.5317 |
| MXL | 64 | C: 76.56%, T: 23.44% | A: 74.22%, G: 25.78% | 0.5299 |
| PUR | 104 | C: 63.46%, T: 36.54% | A: 64.42%, G: 35.58% | 0.4725 |
| CLM | 94 | C: 68.62%, T: 31.38% | A: 68.62%, G: 31.38% | 0.495 |
| PEL | 85 | C: 74.12%, T: 25.88% | A: 65.88%, G: 34.12% | 0.6743 |
| EAS | 504 | C: 90.48%, T: 9.52% | A: 82.34%, G: 17.66% | 0.0007 |
| CHB | 103 | C: 89.81%, T: 10.19% | A: 75.24%, G: 24.76% | 0.0001 |
| JPT | 104 | C: 90.87%, T: 9.13% | A: 79.33%, G: 20.67% | 0 |
| CHS | 105 | C: 88.57%, T: 11.43% | A: 80.95%, G: 19.05% | 0.0005 |
| CDX | 93 | C: 91.4%, T: 8.6% | A: 86.02%, G: 13.98% | 0.0018 |
| KHV | 99 | C: 91.92%, T: 8.08% | A: 90.91%, G: 9.09% | 0.0269 |
| EUR | 503 | C: 55.57%, T: 44.43% | A: 57.06%, G: 42.94% | 0.7554 |
| CEU | 99 | C: 63.64%, T: 36.36% | A: 63.64%, G: 36.36% | 0.833 |
| TSI | 107 | C: 52.34%, T: 47.66% | A: 50.93%, G: 49.07% | 0.8394 |
| FIN | 99 | C: 46.97%, T: 53.03% | A: 51.52%, G: 48.48% | 0.5641 |
| GBR | 91 | C: 56.04%, T: 43.96% | A: 58.24%, G: 41.76% | 0.8717 |
| IBS | 107 | C: 58.88%, T: 41.12% | A: 61.21%, G: 38.79% | 0.6981 |
| SAS | 489 | C: 80.47%, T: 19.53% | A: 81.19%, G: 18.81% | 0.8064 |
| GIH | 103 | C: 78.16%, T: 21.84% | A: 81.07%, G: 18.93% | 0.8355 |
| PJL | 96 | C: 78.12%, T: 21.88% | A: 79.17%, G: 20.83% | 0.8807 |
| BEB | 86 | C: 83.14%, T: 16.86% | A: 83.14%, G: 16.86% | 0.7666 |
| STU | 102 | C: 81.37%, T: 18.63% | A: 81.86%, G: 18.14% | 0.8435 |
| ITU | 102 | C: 81.86%, T: 18.14% | A: 80.88%, G: 19.12% | 0.7035 |

Abbreviations: ACB, African Caribbeans in Barbados; AFR, All African; ALL, All populations; AMR, Ad Mixed American; ASW, Americans of African Ancestry in SW USA; BEB, Bengali from Bangladesh; CDX, Chinese Dai in Xishuangbanna, China; CEU, Utah Residents (CEPH) with Northern and Western European Ancestry; CHB, Han Chinese in Beijing, China; CHS, Southern Han Chinese; CLM, Colombians from Medellin, Colombia; EAS, All East Asian; ESN, Esan in Nigeria; EUR, European; FIN, Finnish in Finland; GBR, British in England and Scotland; GIH, Gujarati Indian from Houston, Texas; GWD, Gambian in Western Divisions in the Gambia; IBS, Iberian Population in Spain; ITU, Indian Telugu from the UK; JPT, Japanese in Tokyo, Japan; KHV, Kinh in Ho Chi Minh City, Vietnam; LWK, Luhya in Webuye, Kenya; MSL, Mende in Sierra Leone; MXL, Mexican Ancestry from Los Angeles USA; PEL, Peruvians from Lima, Peru; PJL, Punjabi from Lahore, Pakistan; PUR, Puerto Ricans from Puerto Rico; SAS, South Asian; STU, Sri Lankan Tamil from the UK; TSI, Toscani in Italia; YRI, Yoruba in Ibadan, Nigeria.

**S13 Table.** Pairwise linkage disequilibrium (R^2^) for rs7194886 and rs2111234 NOD2 SNPs.

| **Population** | **N** | **rs7194886 Allele Freq** | **rs2111234 Allele Freq** | **R^2^** |
| --- | --- | --- | --- | --- |
| ALL | 2504 | C: 74.36%, T: 25.64% | G: 48.7%, A: 51.3% | 0.1844 |
| AFR | 661 | C: 74.21%, T: 25.79% | G: 33.96%, A: 66.04% | 0.1355 |
| YRI | 108 | C: 77.31%, T: 22.69% | G: 35.65%, A: 64.35% | 0.1274 |
| LWK | 99 | C: 77.78%, T: 22.22% | G: 37.37%, A: 62.63% | 0.114 |
| GWD | 113 | C: 72.12%, T: 27.88% | G: 29.65%, A: 70.35% | 0.1459 |
| MSL | 85 | C: 78.24%, T: 21.76% | G: 36.47%, A: 63.53% | 0.1597 |
| ESN | 99 | C: 72.73%, T: 27.27% | G: 31.82%, A: 68.18% | 0.088 |
| ASW | 61 | C: 72.95%, T: 27.05% | G: 34.43%, A: 65.57% | 0.1054 |
| ACB | 96 | C: 68.23%, T: 31.77% | G: 33.33%, A: 66.67% | 0.2105 |
| AMR | 347 | C: 69.88%, T: 30.12% | G: 48.41%, A: 51.59% | 0.2668 |
| MXL | 64 | C: 76.56%, T: 23.44% | G: 58.59%, A: 41.41% | 0.2979 |
| PUR | 104 | C: 63.46%, T: 36.54% | G: 36.54%, A: 63.46% | 0.1854 |
| CLM | 94 | C: 68.62%, T: 31.38% | G: 42.02%, A: 57.98% | 0.1521 |
| PEL | 85 | C: 74.12%, T: 25.88% | G: 62.35%, A: 37.65% | 0.537 |
| EAS | 504 | C: 90.48%, T: 9.52% | G: 76.49%, A: 23.51% | 0 |
| CHB | 103 | C: 89.81%, T: 10.19% | G: 72.33%, A: 27.67% | 0.0008 |
| JPT | 104 | C: 90.87%, T: 9.13% | G: 73.08%, A: 26.92% | 0 |
| CHS | 105 | C: 88.57%, T: 11.43% | G: 78.57%, A: 21.43% | 0.0017 |
| CDX | 93 | C: 91.4%, T: 8.6% | G: 76.88%, A: 23.12% | 0.001 |
| KHV | 99 | C: 91.92%, T: 8.08% | G: 81.82%, A: 18.18% | 0.0101 |
| EUR | 503 | C: 55.57%, T: 44.43% | G: 29.22%, A: 70.78% | 0.1807 |
| CEU | 99 | C: 63.64%, T: 36.36% | G: 28.79%, A: 71.21% | 0.169 |
| TSI | 107 | C: 52.34%, T: 47.66% | G: 26.17%, A: 73.83% | 0.2762 |
| FIN | 99 | C: 46.97%, T: 53.03% | G: 31.31%, A: 68.69% | 0.1356 |
| GBR | 91 | C: 56.04%, T: 43.96% | G: 29.12%, A: 70.88% | 0.2211 |
| IBS | 107 | C: 58.88%, T: 41.12% | G: 30.84%, A: 69.16% | 0.1391 |
| SAS | 489 | C: 80.47%, T: 19.53% | G: 60.22%, A: 39.78% | 0.2835 |
| GIH | 103 | C: 78.16%, T: 21.84% | G: 60.68%, A: 39.32% | 0.2878 |
| PJL | 96 | C: 78.12%, T: 21.88% | G: 55.73%, A: 44.27% | 0.2679 |
| BEB | 86 | C: 83.14%, T: 16.86% | G: 62.79%, A: 37.21% | 0.2712 |
| STU | 102 | C: 81.37%, T: 18.63% | G: 60.78%, A: 39.22% | 0.2686 |
| ITU | 102 | C: 81.86%, T: 18.14% | G: 61.27%, A: 38.73% | 0.3203 |

Abbreviations: ACB, African Caribbeans in Barbados; AFR, All African; ALL, All populations; AMR, Ad Mixed American; ASW, Americans of African Ancestry in SW USA; BEB, Bengali from Bangladesh; CDX, Chinese Dai in Xishuangbanna, China; CEU, Utah Residents (CEPH) with Northern and Western European Ancestry; CHB, Han Chinese in Beijing, China; CHS, Southern Han Chinese; CLM, Colombians from Medellin, Colombia; EAS, All East Asian; ESN, Esan in Nigeria; EUR, European; FIN, Finnish in Finland; GBR, British in England and Scotland; GIH, Gujarati Indian from Houston, Texas; GWD, Gambian in Western Divisions in the Gambia; IBS, Iberian Population in Spain; ITU, Indian Telugu from the UK; JPT, Japanese in Tokyo, Japan; KHV, Kinh in Ho Chi Minh City, Vietnam; LWK, Luhya in Webuye, Kenya; MSL, Mende in Sierra Leone; MXL, Mexican Ancestry from Los Angeles USA; PEL, Peruvians from Lima, Peru; PJL, Punjabi from Lahore, Pakistan; PUR, Puerto Ricans from Puerto Rico; SAS, South Asian; STU, Sri Lankan Tamil from the UK; TSI, Toscani in Italia; YRI, Yoruba in Ibadan, Nigeria.

**S14 Table.** Pairwise linkage disequilibrium (R^2^) for rs9302752 and rs2066842 NOD2 SNPs.

| **Population** | **N** | **rs9302752 Allele Freq** | **rs2066842 Allele Freq** | **R^2^** |
| --- | --- | --- | --- | --- |
| ALL | 2504 | T: 50.02%, C: 49.98% | C: 89.8%, T: 10.2% | 0.1033 |
| AFR | 661 | T: 34.95%, C: 65.05% | C: 97.28%, T: 2.72% | 0.015 |
| YRI | 108 | T: 39.81%, C: 60.19% | C: 99.54%, T: 0.46% | 0.0031 |
| LWK | 99 | T: 34.85%, C: 65.15% | C: 98.48%, T: 1.52% | 0.0082 |
| GWD | 113 | T: 31.86%, C: 68.14% | C: 95.13%, T: 4.87% | 0.0239 |
| MSL | 85 | T: 41.76%, C: 58.24% | C: 99.41%, T: 0.59% | 0.0042 |
| ESN | 99 | T: 30.3%, C: 69.7% | C: 99.49%, T: 0.51% | 0.0022 |
| ASW | 61 | T: 35.25%, C: 64.75% | C: 88.52%, T: 11.48% | 0.0706 |
| ACB | 96 | T: 31.77%, C: 68.23% | C: 97.4%, T: 2.6% | 0.0125 |
| AMR | 347 | T: 45.1%, C: 54.9% | C: 83.29%, T: 16.71% | 0.1586 |
| MXL | 64 | T: 57.03%, C: 42.97% | C: 85.16%, T: 14.84% | 0.1906 |
| PUR | 104 | T: 28.37%, C: 71.63% | C: 77.4%, T: 22.6% | 0.1156 |
| CLM | 94 | T: 39.36%, C: 60.64% | C: 78.72%, T: 21.28% | 0.1754 |
| PEL | 85 | T: 62.94%, C: 37.06% | C: 94.12%, T: 5.88% | 0.1062 |
| EAS | 504 | T: 77.88%, C: 22.12% | C: 99.21%, T: 0.79% | 0.0282 |
| CHB | 103 | T: 72.33%, C: 27.67% | C: 100.0%, T: 0.0% | NA |
| JPT | 104 | T: 75.96%, C: 24.04% | C: 100.0%, T: 0.0% | NA |
| CHS | 105 | T: 76.19%, C: 23.81% | C: 99.52%, T: 0.48% | 0.0153 |
| CDX | 93 | T: 82.26%, C: 17.74% | C: 98.92%, T: 1.08% | 0.0504 |
| KHV | 99 | T: 83.33%, C: 16.67% | C: 97.47%, T: 2.53% | 0.1295 |
| EUR | 503 | T: 25.45%, C: 74.55% | C: 75.35%, T: 24.65% | 0.1047 |
| CEU | 99 | T: 27.78%, C: 72.22% | C: 68.18%, T: 31.82% | 0.1408 |
| TSI | 107 | T: 24.3%, C: 75.7% | C: 76.17%, T: 23.83% | 0.1004 |
| FIN | 99 | T: 23.74%, C: 76.26% | C: 85.86%, T: 14.14% | 0.0513 |
| GBR | 91 | T: 25.27%, C: 74.73% | C: 75.27%, T: 24.73% | 0.1111 |
| IBS | 107 | T: 26.17%, C: 73.83% | C: 71.5%, T: 28.5% | 0.1413 |
| SAS | 489 | T: 70.45%, C: 29.55% | C: 89.47%, T: 10.53% | 0.2153 |
| GIH | 103 | T: 70.39%, C: 29.61% | C: 92.23%, T: 7.77% | 0.1354 |
| PJL | 96 | T: 63.54%, C: 36.46% | C: 86.46%, T: 13.54% | 0.273 |
| BEB | 86 | T: 71.51%, C: 28.49% | C: 91.28%, T: 8.72% | 0.2398 |
| STU | 102 | T: 74.51%, C: 25.49% | C: 88.73%, T: 11.27% | 0.1863 |
| ITU | 102 | T: 72.06%, C: 27.94% | C: 88.73%, T: 11.27% | 0.2534 |

Abbreviations: ACB, African Caribbeans in Barbados; AFR, All African; ALL, All populations; AMR, Ad Mixed American; ASW, Americans of African Ancestry in SW USA; BEB, Bengali from Bangladesh; CDX, Chinese Dai in Xishuangbanna, China; CEU, Utah Residents (CEPH) with Northern and Western European Ancestry; CHB, Han Chinese in Beijing, China; CHS, Southern Han Chinese; CLM, Colombians from Medellin, Colombia; EAS, All East Asian; ESN, Esan in Nigeria; EUR, European; FIN, Finnish in Finland; GBR, British in England and Scotland; GIH, Gujarati Indian from Houston, Texas; GWD, Gambian in Western Divisions in the Gambia; IBS, Iberian Population in Spain; ITU, Indian Telugu from the UK; JPT, Japanese in Tokyo, Japan; KHV, Kinh in Ho Chi Minh City, Vietnam; LWK, Luhya in Webuye, Kenya; MSL, Mende in Sierra Leone; MXL, Mexican Ancestry from Los Angeles USA; PEL, Peruvians from Lima, Peru; PJL, Punjabi from Lahore, Pakistan; PUR, Puerto Ricans from Puerto Rico; SAS, South Asian; STU, Sri Lankan Tamil from the UK; TSI, Toscani in Italia; YRI, Yoruba in Ibadan, Nigeria.

**S15 Table.** Pairwise linkage disequilibrium (R^2^) for rs9302752 and rs2066844 NOD2 SNPs.

| **Population** | **N** | **rs9302752 Allele Freq** | **rs2066844 Allele Freq** | **R^2^** |
| --- | --- | --- | --- | --- |
| ALL | 2504 | T: 50.02%, C: 49.98% | C: 98.56%, T: 1.44% | 0.0138 |
| AFR | 661 | T: 34.95%, C: 65.05% | C: 99.77%, T: 0.23% | 0.0012 |
| YRI | 108 | T: 39.81%, C: 60.19% | C: 100.0%, T: 0.0% | NA |
| LWK | 99 | T: 34.85%, C: 65.15% | C: 100.0%, T: 0.0% | NA |
| GWD | 113 | T: 31.86%, C: 68.14% | C: 100.0%, T: 0.0% | NA |
| MSL | 85 | T: 41.76%, C: 58.24% | C: 100.0%, T: 0.0% | NA |
| ESN | 99 | T: 30.3%, C: 69.7% | C: 100.0%, T: 0.0% | NA |
| ASW | 61 | T: 35.25%, C: 64.75% | C: 98.36%, T: 1.64% | 0.0091 |
| ACB | 96 | T: 31.77%, C: 68.23% | C: 99.48%, T: 0.52% | 0.0024 |
| AMR | 347 | T: 45.1%, C: 54.9% | C: 97.55%, T: 2.45% | 0.0206 |
| MXL | 64 | T: 57.03%, C: 42.97% | C: 100.0%, T: 0.0% | NA |
| PUR | 104 | T: 28.37%, C: 71.63% | C: 97.12%, T: 2.88% | 0.0118 |
| CLM | 94 | T: 39.36%, C: 60.64% | C: 94.68%, T: 5.32% | 0.0365 |
| PEL | 85 | T: 62.94%, C: 37.06% | C: 99.41%, T: 0.59% | 0.01 |
| EAS | 504 | T: 77.88%, C: 22.12% | C: 100.0%, T: 0.0% | NA |
| CHB | 103 | T: 72.33%, C: 27.67% | C: 100.0%, T: 0.0% | NA |
| JPT | 104 | T: 75.96%, C: 24.04% | C: 100.0%, T: 0.0% | NA |
| CHS | 105 | T: 76.19%, C: 23.81% | C: 100.0%, T: 0.0% | NA |
| CDX | 93 | T: 82.26%, C: 17.74% | C: 100.0%, T: 0.0% | NA |
| KHV | 99 | T: 83.33%, C: 16.67% | C: 100.0%, T: 0.0% | NA |
| EUR | 503 | T: 25.45%, C: 74.55% | C: 94.93%, T: 5.07% | 0.0155 |
| CEU | 99 | T: 27.78%, C: 72.22% | C: 92.93%, T: 7.07% | 0.0161 |
| TSI | 107 | T: 24.3%, C: 75.7% | C: 95.33%, T: 4.67% | 0.0157 |
| FIN | 99 | T: 23.74%, C: 76.26% | C: 96.97%, T: 3.03% | 0.0097 |
| GBR | 91 | T: 25.27%, C: 74.73% | C: 95.05%, T: 4.95% | 0.0176 |
| IBS | 107 | T: 26.17%, C: 73.83% | C: 94.39%, T: 5.61% | 0.0211 |
| SAS | 489 | T: 70.45%, C: 29.55% | C: 99.9%, T: 0.1% | 0.0024 |
| GIH | 103 | T: 70.39%, C: 29.61% | C: 100.0%, T: 0.0% | NA |
| PJL | 96 | T: 63.54%, C: 36.46% | C: 100.0%, T: 0.0% | NA |
| BEB | 86 | T: 71.51%, C: 28.49% | C: 99.42%, T: 0.58% | 0.0147 |
| STU | 102 | T: 74.51%, C: 25.49% | C: 100.0%, T: 0.0% | NA |
| ITU | 102 | T: 72.06%, C: 27.94% | C: 100.0%, T: 0.0% | NA |

Abbreviations: ACB, African Caribbeans in Barbados; AFR, All African; ALL, All populations; AMR, Ad Mixed American; ASW, Americans of African Ancestry in SW USA; BEB, Bengali from Bangladesh; CDX, Chinese Dai in Xishuangbanna, China; CEU, Utah Residents (CEPH) with Northern and Western European Ancestry; CHB, Han Chinese in Beijing, China; CHS, Southern Han Chinese; CLM, Colombians from Medellin, Colombia; EAS, All East Asian; ESN, Esan in Nigeria; EUR, European; FIN, Finnish in Finland; GBR, British in England and Scotland; GIH, Gujarati Indian from Houston, Texas; GWD, Gambian in Western Divisions in the Gambia; IBS, Iberian Population in Spain; ITU, Indian Telugu from the UK; JPT, Japanese in Tokyo, Japan; KHV, Kinh in Ho Chi Minh City, Vietnam; LWK, Luhya in Webuye, Kenya; MSL, Mende in Sierra Leone; MXL, Mexican Ancestry from Los Angeles USA; PEL, Peruvians from Lima, Peru; PJL, Punjabi from Lahore, Pakistan; PUR, Puerto Ricans from Puerto Rico; SAS, South Asian; STU, Sri Lankan Tamil from the UK; TSI, Toscani in Italia; YRI, Yoruba in Ibadan, Nigeria.

**S16 Table.** Pairwise linkage disequilibrium (R^2^) for rs9302752 and rs5743278 NOD2 SNPs.

| **Population** | **N** | **rs9302752 Allele Freq** | **rs5743278 Allele Freq** | **R^2^** |
| --- | --- | --- | --- | --- |
| ALL | 2504 | T: 50.02%, C: 49.98% | C: 98.16%, G: 1.84% | 0.0171 |
| AFR | 661 | T: 34.95%, C: 65.05% | C: 93.27%, G: 6.73% | 0.034 |
| YRI | 108 | T: 39.81%, C: 60.19% | C: 93.06%, G: 6.94% | 0.0494 |
| LWK | 99 | T: 34.85%, C: 65.15% | C: 88.89%, G: 11.11% | 0.0669 |
| GWD | 113 | T: 31.86%, C: 68.14% | C: 95.58%, G: 4.42% | 0.0216 |
| MSL | 85 | T: 41.76%, C: 58.24% | C: 93.53%, G: 6.47% | 0.0496 |
| ESN | 99 | T: 30.3%, C: 69.7% | C: 93.43%, G: 6.57% | 0.017 |
| ASW | 61 | T: 35.25%, C: 64.75% | C: 93.44%, G: 6.56% | 0.0382 |
| ACB | 96 | T: 31.77%, C: 68.23% | C: 94.79%, G: 5.21% | 0.012 |
| AMR | 347 | T: 45.1%, C: 54.9% | C: 99.57%, G: 0.43% | 0.0036 |
| MXL | 64 | T: 57.03%, C: 42.97% | C: 100.0%, G: 0.0% | NA |
| PUR | 104 | T: 28.37%, C: 71.63% | C: 99.52%, G: 0.48% | 0.0019 |
| CLM | 94 | T: 39.36%, C: 60.64% | C: 99.47%, G: 0.53% | 0.0035 |
| PEL | 85 | T: 62.94%, C: 37.06% | C: 99.41%, G: 0.59% | 0.01 |
| EAS | 504 | T: 77.88%, C: 22.12% | C: 100.0%, G: 0.0% | NA |
| CHB | 103 | T: 72.33%, C: 27.67% | C: 100.0%, G: 0.0% | NA |
| JPT | 104 | T: 75.96%, C: 24.04% | C: 100.0%, G: 0.0% | NA |
| CHS | 105 | T: 76.19%, C: 23.81% | C: 100.0%, G: 0.0% | NA |
| CDX | 93 | T: 82.26%, C: 17.74% | C: 100.0%, G: 0.0% | NA |
| KHV | 99 | T: 83.33%, C: 16.67% | C: 100.0%, G: 0.0% | NA |
| EUR | 503 | T: 25.45%, C: 74.55% | C: 100.0%, G: 0.0% | NA |
| CEU | 99 | T: 27.78%, C: 72.22% | C: 100.0%, G: 0.0% | NA |
| TSI | 107 | T: 24.3%, C: 75.7% | C: 100.0%, G: 0.0% | NA |
| FIN | 99 | T: 23.74%, C: 76.26% | C: 100.0%, G: 0.0% | NA |
| GBR | 91 | T: 25.27%, C: 74.73% | C: 100.0%, G: 0.0% | NA |
| IBS | 107 | T: 26.17%, C: 73.83% | C: 100.0%, G: 0.0% | NA |
| SAS | 489 | T: 70.45%, C: 29.55% | C: 100.0%, G: 0.0% | NA |
| GIH | 103 | T: 70.39%, C: 29.61% | C: 100.0%, G: 0.0% | NA |
| PJL | 96 | T: 63.54%, C: 36.46% | C: 100.0%, G: 0.0% | NA |
| BEB | 86 | T: 71.51%, C: 28.49% | C: 100.0%, G: 0.0% | NA |
| STU | 102 | T: 74.51%, C: 25.49% | C: 100.0%, G: 0.0% | NA |
| ITU | 102 | T: 72.06%, C: 27.94% | C: 100.0%, G: 0.0% | NA |

Abbreviations: ACB, African Caribbeans in Barbados; AFR, All African; ALL, All populations; AMR, Ad Mixed American; ASW, Americans of African Ancestry in SW USA; BEB, Bengali from Bangladesh; CDX, Chinese Dai in Xishuangbanna, China; CEU, Utah Residents (CEPH) with Northern and Western European Ancestry; CHB, Han Chinese in Beijing, China; CHS, Southern Han Chinese; CLM, Colombians from Medellin, Colombia; EAS, All East Asian; ESN, Esan in Nigeria; EUR, European; FIN, Finnish in Finland; GBR, British in England and Scotland; GIH, Gujarati Indian from Houston, Texas; GWD, Gambian in Western Divisions in the Gambia; IBS, Iberian Population in Spain; ITU, Indian Telugu from the UK; JPT, Japanese in Tokyo, Japan; KHV, Kinh in Ho Chi Minh City, Vietnam; LWK, Luhya in Webuye, Kenya; MSL, Mende in Sierra Leone; MXL, Mexican Ancestry from Los Angeles USA; PEL, Peruvians from Lima, Peru; PJL, Punjabi from Lahore, Pakistan; PUR, Puerto Ricans from Puerto Rico; SAS, South Asian; STU, Sri Lankan Tamil from the UK; TSI, Toscani in Italia; YRI, Yoruba in Ibadan, Nigeria.

**S17 Table.** Pairwise linkage disequilibrium (R^2^) for rs9302752 and rs6500328 NOD2 SNPs.

| **Population** | **N** | **rs9302752 Allele Freq** | **rs6500328 Allele Freq** | **R^2^** |
| --- | --- | --- | --- | --- |
| ALL | 2504 | T: 50.02%, C: 49.98% | A: 67.25%, G: 32.75% | 0.3196 |
| AFR | 661 | T: 34.95%, C: 65.05% | A: 52.95%, G: 47.05% | 0.1665 |
| YRI | 108 | T: 39.81%, C: 60.19% | A: 53.24%, G: 46.76% | 0.1468 |
| LWK | 99 | T: 34.85%, C: 65.15% | A: 46.46%, G: 53.54% | 0.2809 |
| GWD | 113 | T: 31.86%, C: 68.14% | A: 57.52%, G: 42.48% | 0.1015 |
| MSL | 85 | T: 41.76%, C: 58.24% | A: 53.53%, G: 46.47% | 0.2063 |
| ESN | 99 | T: 30.3%, C: 69.7% | A: 50.51%, G: 49.49% | 0.1689 |
| ASW | 61 | T: 35.25%, C: 64.75% | A: 60.66%, G: 39.34% | 0.147 |
| ACB | 96 | T: 31.77%, C: 68.23% | A: 51.04%, G: 48.96% | 0.1598 |
| AMR | 347 | T: 45.1%, C: 54.9% | A: 67.72%, G: 32.28% | 0.3464 |
| MXL | 64 | T: 57.03%, C: 42.97% | A: 74.22%, G: 25.78% | 0.4611 |
| PUR | 104 | T: 28.37%, C: 71.63% | A: 64.42%, G: 35.58% | 0.1983 |
| CLM | 94 | T: 39.36%, C: 60.64% | A: 68.62%, G: 31.38% | 0.248 |
| PEL | 85 | T: 62.94%, C: 37.06% | A: 65.88%, G: 34.12% | 0.7409 |
| EAS | 504 | T: 77.88%, C: 22.12% | A: 82.34%, G: 17.66% | 0.3517 |
| CHB | 103 | T: 72.33%, C: 27.67% | A: 75.24%, G: 24.76% | 0.457 |
| JPT | 104 | T: 75.96%, C: 24.04% | A: 79.33%, G: 20.67% | 0.4322 |
| CHS | 105 | T: 76.19%, C: 23.81% | A: 80.95%, G: 19.05% | 0.2767 |
| CDX | 93 | T: 82.26%, C: 17.74% | A: 86.02%, G: 13.98% | 0.2527 |
| KHV | 99 | T: 83.33%, C: 16.67% | A: 90.91%, G: 9.09% | 0.2689 |
| EUR | 503 | T: 25.45%, C: 74.55% | A: 57.06%, G: 42.94% | 0.2522 |
| CEU | 99 | T: 27.78%, C: 72.22% | A: 63.64%, G: 36.36% | 0.2198 |
| TSI | 107 | T: 24.3%, C: 75.7% | A: 50.93%, G: 49.07% | 0.2854 |
| FIN | 99 | T: 23.74%, C: 76.26% | A: 51.52%, G: 48.48% | 0.2929 |
| GBR | 91 | T: 25.27%, C: 74.73% | A: 58.24%, G: 41.76% | 0.2425 |
| IBS | 107 | T: 26.17%, C: 73.83% | A: 61.21%, G: 38.79% | 0.2246 |
| SAS | 489 | T: 70.45%, C: 29.55% | A: 81.19%, G: 18.81% | 0.4245 |
| GIH | 103 | T: 70.39%, C: 29.61% | A: 81.07%, G: 18.93% | 0.5154 |
| PJL | 96 | T: 63.54%, C: 36.46% | A: 79.17%, G: 20.83% | 0.3893 |
| BEB | 86 | T: 71.51%, C: 28.49% | A: 83.14%, G: 16.86% | 0.4612 |
| STU | 102 | T: 74.51%, C: 25.49% | A: 81.86%, G: 18.14% | 0.3964 |
| ITU | 102 | T: 72.06%, C: 27.94% | A: 80.88%, G: 19.12% | 0.3771 |

Abbreviations: ACB, African Caribbeans in Barbados; AFR, All African; ALL, All populations; AMR, Ad Mixed American; ASW, Americans of African Ancestry in SW USA; BEB, Bengali from Bangladesh; CDX, Chinese Dai in Xishuangbanna, China; CEU, Utah Residents (CEPH) with Northern and Western European Ancestry; CHB, Han Chinese in Beijing, China; CHS, Southern Han Chinese; CLM, Colombians from Medellin, Colombia; EAS, All East Asian; ESN, Esan in Nigeria; EUR, European; FIN, Finnish in Finland; GBR, British in England and Scotland; GIH, Gujarati Indian from Houston, Texas; GWD, Gambian in Western Divisions in the Gambia; IBS, Iberian Population in Spain; ITU, Indian Telugu from the UK; JPT, Japanese in Tokyo, Japan; KHV, Kinh in Ho Chi Minh City, Vietnam; LWK, Luhya in Webuye, Kenya; MSL, Mende in Sierra Leone; MXL, Mexican Ancestry from Los Angeles USA; PEL, Peruvians from Lima, Peru; PJL, Punjabi from Lahore, Pakistan; PUR, Puerto Ricans from Puerto Rico; SAS, South Asian; STU, Sri Lankan Tamil from the UK; TSI, Toscani in Italia; YRI, Yoruba in Ibadan, Nigeria.

**S18 Table.** Pairwise linkage disequilibrium (R^2^) for rs9302752 and rs2111234 NOD2 SNPs.

| **Population** | **N** | **rs9302752 Allele Freq** | **rs2111234 Allele Freq** | **R^2^** |
| --- | --- | --- | --- | --- |
| ALL | 2504 | T: 50.02%, C: 49.98% | G: 48.7%, A: 51.3% | 0.5498 |
| AFR | 661 | T: 34.95%, C: 65.05% | G: 33.96%, A: 66.04% | 0.3844 |
| YRI | 108 | T: 39.81%, C: 60.19% | G: 35.65%, A: 64.35% | 0.3132 |
| LWK | 99 | T: 34.85%, C: 65.15% | G: 37.37%, A: 62.63% | 0.3298 |
| GWD | 113 | T: 31.86%, C: 68.14% | G: 29.65%, A: 70.35% | 0.581 |
| MSL | 85 | T: 41.76%, C: 58.24% | G: 36.47%, A: 63.53% | 0.4185 |
| ESN | 99 | T: 30.3%, C: 69.7% | G: 31.82%, A: 68.18% | 0.2207 |
| ASW | 61 | T: 35.25%, C: 64.75% | G: 34.43%, A: 65.57% | 0.5319 |
| ACB | 96 | T: 31.77%, C: 68.23% | G: 33.33%, A: 66.67% | 0.4005 |
| AMR | 347 | T: 45.1%, C: 54.9% | G: 48.41%, A: 51.59% | 0.7702 |
| MXL | 64 | T: 57.03%, C: 42.97% | G: 58.59%, A: 41.41% | 0.8769 |
| PUR | 104 | T: 28.37%, C: 71.63% | G: 36.54%, A: 63.46% | 0.6162 |
| CLM | 94 | T: 39.36%, C: 60.64% | G: 42.02%, A: 57.98% | 0.7364 |
| PEL | 85 | T: 62.94%, C: 37.06% | G: 62.35%, A: 37.65% | 0.8784 |
| EAS | 504 | T: 77.88%, C: 22.12% | G: 76.49%, A: 23.51% | 0.2272 |
| CHB | 103 | T: 72.33%, C: 27.67% | G: 72.33%, A: 27.67% | 0.2907 |
| JPT | 104 | T: 75.96%, C: 24.04% | G: 73.08%, A: 26.92% | 0.2714 |
| CHS | 105 | T: 76.19%, C: 23.81% | G: 78.57%, A: 21.43% | 0.1969 |
| CDX | 93 | T: 82.26%, C: 17.74% | G: 76.88%, A: 23.12% | 0.1441 |
| KHV | 99 | T: 83.33%, C: 16.67% | G: 81.82%, A: 18.18% | 0.2086 |
| EUR | 503 | T: 25.45%, C: 74.55% | G: 29.22%, A: 70.78% | 0.7379 |
| CEU | 99 | T: 27.78%, C: 72.22% | G: 28.79%, A: 71.21% | 0.8567 |
| TSI | 107 | T: 24.3%, C: 75.7% | G: 26.17%, A: 73.83% | 0.8591 |
| FIN | 99 | T: 23.74%, C: 76.26% | G: 31.31%, A: 68.69% | 0.4188 |
| GBR | 91 | T: 25.27%, C: 74.73% | G: 29.12%, A: 70.88% | 0.8233 |
| IBS | 107 | T: 26.17%, C: 73.83% | G: 30.84%, A: 69.16% | 0.7948 |
| SAS | 489 | T: 70.45%, C: 29.55% | G: 60.22%, A: 39.78% | 0.5171 |
| GIH | 103 | T: 70.39%, C: 29.61% | G: 60.68%, A: 39.32% | 0.4558 |
| PJL | 96 | T: 63.54%, C: 36.46% | G: 55.73%, A: 44.27% | 0.6501 |
| BEB | 86 | T: 71.51%, C: 28.49% | G: 62.79%, A: 37.21% | 0.4715 |
| STU | 102 | T: 74.51%, C: 25.49% | G: 60.78%, A: 39.22% | 0.4344 |
| ITU | 102 | T: 72.06%, C: 27.94% | G: 61.27%, A: 38.73% | 0.5789 |

Abbreviations: ACB, African Caribbeans in Barbados; AFR, All African; ALL, All populations; AMR, Ad Mixed American; ASW, Americans of African Ancestry in SW USA; BEB, Bengali from Bangladesh; CDX, Chinese Dai in Xishuangbanna, China; CEU, Utah Residents (CEPH) with Northern and Western European Ancestry; CHB, Han Chinese in Beijing, China; CHS, Southern Han Chinese; CLM, Colombians from Medellin, Colombia; EAS, All East Asian; ESN, Esan in Nigeria; EUR, European; FIN, Finnish in Finland; GBR, British in England and Scotland; GIH, Gujarati Indian from Houston, Texas; GWD, Gambian in Western Divisions in the Gambia; IBS, Iberian Population in Spain; ITU, Indian Telugu from the UK; JPT, Japanese in Tokyo, Japan; KHV, Kinh in Ho Chi Minh City, Vietnam; LWK, Luhya in Webuye, Kenya; MSL, Mende in Sierra Leone; MXL, Mexican Ancestry from Los Angeles USA; PEL, Peruvians from Lima, Peru; PJL, Punjabi from Lahore, Pakistan; PUR, Puerto Ricans from Puerto Rico; SAS, South Asian; STU, Sri Lankan Tamil from the UK; TSI, Toscani in Italia; YRI, Yoruba in Ibadan, Nigeria.

**S19 Table.** Pairwise linkage disequilibrium (R^2^) for rs2066842 and rs2066844 NOD2 SNPs.

| **Population** | **N** | **rs2066842 Allele Freq** | **rs2066844 Allele Freq** | **R^2^** |
| --- | --- | --- | --- | --- |
| ALL | 2504 | C: 89.8%, T: 10.2% | C: 98.56%, T: 1.44% | 0.1284 |
| AFR | 661 | C: 97.28%, T: 2.72% | C: 99.77%, T: 0.23% | 0.0812 |
| YRI | 108 | C: 99.54%, T: 0.46% | C: 100.0%, T: 0.0% | NA |
| LWK | 99 | C: 98.48%, T: 1.52% | C: 100.0%, T: 0.0% | NA |
| GWD | 113 | C: 95.13%, T: 4.87% | C: 100.0%, T: 0.0% | NA |
| MSL | 85 | C: 99.41%, T: 0.59% | C: 100.0%, T: 0.0% | NA |
| ESN | 99 | C: 99.49%, T: 0.51% | C: 100.0%, T: 0.0% | NA |
| ASW | 61 | C: 88.52%, T: 11.48% | C: 98.36%, T: 1.64% | 0.1286 |
| ACB | 96 | C: 97.4%, T: 2.6% | C: 99.48%, T: 0.52% | 0.1958 |
| AMR | 347 | C: 83.29%, T: 16.71% | C: 97.55%, T: 2.45% | 0.1251 |
| MXL | 64 | C: 85.16%, T: 14.84% | C: 100.0%, T: 0.0% | NA |
| PUR | 104 | C: 77.4%, T: 22.6% | C: 97.12%, T: 2.88% | 0.1017 |
| CLM | 94 | C: 78.72%, T: 21.28% | C: 94.68%, T: 5.32% | 0.2079 |
| PEL | 85 | C: 94.12%, T: 5.88% | C: 99.41%, T: 0.59% | 0.0947 |
| EAS | 504 | C: 99.21%, T: 0.79% | C: 100.0%, T: 0.0% | NA |
| CHB | 103 | C: 100.0%, T: 0.0% | C: 100.0%, T: 0.0% | NA |
| JPT | 104 | C: 100.0%, T: 0.0% | C: 100.0%, T: 0.0% | NA |
| CHS | 105 | C: 99.52%, T: 0.48% | C: 100.0%, T: 0.0% | NA |
| CDX | 93 | C: 98.92%, T: 1.08% | C: 100.0%, T: 0.0% | NA |
| KHV | 99 | C: 97.47%, T: 2.53% | C: 100.0%, T: 0.0% | NA |
| EUR | 503 | C: 75.35%, T: 24.65% | C: 94.93%, T: 5.07% | 0.1632 |
| CEU | 99 | C: 68.18%, T: 31.82% | C: 92.93%, T: 7.07% | 0.163 |
| TSI | 107 | C: 76.17%, T: 23.83% | C: 95.33%, T: 4.67% | 0.1567 |
| FIN | 99 | C: 85.86%, T: 14.14% | C: 96.97%, T: 3.03% | 0.1897 |
| GBR | 91 | C: 75.27%, T: 24.73% | C: 95.05%, T: 4.95% | 0.1584 |
| IBS | 107 | C: 71.5%, T: 28.5% | C: 94.39%, T: 5.61% | 0.149 |
| SAS | 489 | C: 89.47%, T: 10.53% | C: 99.9%, T: 0.1% | 0.0087 |
| GIH | 103 | C: 92.23%, T: 7.77% | C: 100.0%, T: 0.0% | NA |
| PJL | 96 | C: 86.46%, T: 13.54% | C: 100.0%, T: 0.0% | NA |
| BEB | 86 | C: 91.28%, T: 8.72% | C: 99.42%, T: 0.58% | 0.0612 |
| STU | 102 | C: 88.73%, T: 11.27% | C: 100.0%, T: 0.0% | NA |
| ITU | 102 | C: 88.73%, T: 11.27% | C: 100.0%, T: 0.0% | NA |

Abbreviations: ACB, African Caribbeans in Barbados; AFR, All African; ALL, All populations; AMR, Ad Mixed American; ASW, Americans of African Ancestry in SW USA; BEB, Bengali from Bangladesh; CDX, Chinese Dai in Xishuangbanna, China; CEU, Utah Residents (CEPH) with Northern and Western European Ancestry; CHB, Han Chinese in Beijing, China; CHS, Southern Han Chinese; CLM, Colombians from Medellin, Colombia; EAS, All East Asian; ESN, Esan in Nigeria; EUR, European; FIN, Finnish in Finland; GBR, British in England and Scotland; GIH, Gujarati Indian from Houston, Texas; GWD, Gambian in Western Divisions in the Gambia; IBS, Iberian Population in Spain; ITU, Indian Telugu from the UK; JPT, Japanese in Tokyo, Japan; KHV, Kinh in Ho Chi Minh City, Vietnam; LWK, Luhya in Webuye, Kenya; MSL, Mende in Sierra Leone; MXL, Mexican Ancestry from Los Angeles USA; PEL, Peruvians from Lima, Peru; PJL, Punjabi from Lahore, Pakistan; PUR, Puerto Ricans from Puerto Rico; SAS, South Asian; STU, Sri Lankan Tamil from the UK; TSI, Toscani in Italia; YRI, Yoruba in Ibadan, Nigeria.

**S20 Table.** Pairwise linkage disequilibrium (R^2^) for rs2066842 and rs5743278 NOD2 SNPs.

| **Population** | **N** | **rs2066842 Allele Freq** | **rs5743278 Allele Freq** | **R^2^** |
| --- | --- | --- | --- | --- |
| ALL | 2504 | C: 89.8%, T: 10.2% | C: 98.16%, G: 1.84% | 0.0021 |
| AFR | 661 | C: 97.28%, T: 2.72% | C: 93.27%, G: 6.73% | 0.002 |
| YRI | 108 | C: 99.54%, T: 0.46% | C: 93.06%, G: 6.94% | 0.0003 |
| LWK | 99 | C: 98.48%, T: 1.52% | C: 88.89%, G: 11.11% | 0.0019 |
| GWD | 113 | C: 95.13%, T: 4.87% | C: 95.58%, G: 4.42% | 0.0024 |
| MSL | 85 | C: 99.41%, T: 0.59% | C: 93.53%, G: 6.47% | 0.0004 |
| ESN | 99 | C: 99.49%, T: 0.51% | C: 93.43%, G: 6.57% | 0.0004 |
| ASW | 61 | C: 88.52%, T: 11.48% | C: 93.44%, G: 6.56% | 0.0091 |
| ACB | 96 | C: 97.4%, T: 2.6% | C: 94.79%, G: 5.21% | 0.0015 |
| AMR | 347 | C: 83.29%, T: 16.71% | C: 99.57%, G: 0.43% | 0.0009 |
| MXL | 64 | C: 85.16%, T: 14.84% | C: 100.0%, G: 0.0% | NA |
| PUR | 104 | C: 77.4%, T: 22.6% | C: 99.52%, G: 0.48% | 0.0014 |
| CLM | 94 | C: 78.72%, T: 21.28% | C: 99.47%, G: 0.53% | 0.0014 |
| PEL | 85 | C: 94.12%, T: 5.88% | C: 99.41%, G: 0.59% | 0.0004 |
| EAS | 504 | C: 99.21%, T: 0.79% | C: 100.0%, G: 0.0% | NA |
| CHB | 103 | C: 100.0%, T: 0.0% | C: 100.0%, G: 0.0% | NA |
| JPT | 104 | C: 100.0%, T: 0.0% | C: 100.0%, G: 0.0% | NA |
| CHS | 105 | C: 99.52%, T: 0.48% | C: 100.0%, G: 0.0% | NA |
| CDX | 93 | C: 98.92%, T: 1.08% | C: 100.0%, G: 0.0% | NA |
| KHV | 99 | C: 97.47%, T: 2.53% | C: 100.0%, G: 0.0% | NA |
| EUR | 503 | C: 75.35%, T: 24.65% | C: 100.0%, G: 0.0% | NA |
| CEU | 99 | C: 68.18%, T: 31.82% | C: 100.0%, G: 0.0% | NA |
| TSI | 107 | C: 76.17%, T: 23.83% | C: 100.0%, G: 0.0% | NA |
| FIN | 99 | C: 85.86%, T: 14.14% | C: 100.0%, G: 0.0% | NA |
| GBR | 91 | C: 75.27%, T: 24.73% | C: 100.0%, G: 0.0% | NA |
| IBS | 107 | C: 71.5%, T: 28.5% | C: 100.0%, G: 0.0% | NA |
| SAS | 489 | C: 89.47%, T: 10.53% | C: 100.0%, G: 0.0% | NA |
| GIH | 103 | C: 92.23%, T: 7.77% | C: 100.0%, G: 0.0% | NA |
| PJL | 96 | C: 86.46%, T: 13.54% | C: 100.0%, G: 0.0% | NA |
| BEB | 86 | C: 91.28%, T: 8.72% | C: 100.0%, G: 0.0% | NA |
| STU | 102 | C: 88.73%, T: 11.27% | C: 100.0%, G: 0.0% | NA |
| ITU | 102 | C: 88.73%, T: 11.27% | C: 100.0%, G: 0.0% | NA |

Abbreviations: ACB, African Caribbeans in Barbados; AFR, All African; ALL, All populations; AMR, Ad Mixed American; ASW, Americans of African Ancestry in SW USA; BEB, Bengali from Bangladesh; CDX, Chinese Dai in Xishuangbanna, China; CEU, Utah Residents (CEPH) with Northern and Western European Ancestry; CHB, Han Chinese in Beijing, China; CHS, Southern Han Chinese; CLM, Colombians from Medellin, Colombia; EAS, All East Asian; ESN, Esan in Nigeria; EUR, European; FIN, Finnish in Finland; GBR, British in England and Scotland; GIH, Gujarati Indian from Houston, Texas; GWD, Gambian in Western Divisions in the Gambia; IBS, Iberian Population in Spain; ITU, Indian Telugu from the UK; JPT, Japanese in Tokyo, Japan; KHV, Kinh in Ho Chi Minh City, Vietnam; LWK, Luhya in Webuye, Kenya; MSL, Mende in Sierra Leone; MXL, Mexican Ancestry from Los Angeles USA; PEL, Peruvians from Lima, Peru; PJL, Punjabi from Lahore, Pakistan; PUR, Puerto Ricans from Puerto Rico; SAS, South Asian; STU, Sri Lankan Tamil from the UK; TSI, Toscani in Italia; YRI, Yoruba in Ibadan, Nigeria.

**S21 Table.** Pairwise linkage disequilibrium (R^2^) for rs2066842 and rs6500328 NOD2 SNPs.

| **Population** | **N** | **rs2066842 Allele Freq** | **rs6500328 Allele Freq** | **R^2^** |
| --- | --- | --- | --- | --- |
| ALL | 2504 | C: 89.8%, T: 10.2% | A: 67.25%, G: 32.75% | 0.0514 |
| AFR | 661 | C: 97.28%, T: 2.72% | A: 52.95%, G: 47.05% | 0.0249 |
| YRI | 108 | C: 99.54%, T: 0.46% | A: 53.24%, G: 46.76% | 0.0041 |
| LWK | 99 | C: 98.48%, T: 1.52% | A: 46.46%, G: 53.54% | 0.0177 |
| GWD | 113 | C: 95.13%, T: 4.87% | A: 57.52%, G: 42.48% | 0.0378 |
| MSL | 85 | C: 99.41%, T: 0.59% | A: 53.53%, G: 46.47% | 0.0051 |
| ESN | 99 | C: 99.49%, T: 0.51% | A: 50.51%, G: 49.49% | 0.005 |
| ASW | 61 | C: 88.52%, T: 11.48% | A: 60.66%, G: 39.34% | 0.0841 |
| ACB | 96 | C: 97.4%, T: 2.6% | A: 51.04%, G: 48.96% | 0.0256 |
| AMR | 347 | C: 83.29%, T: 16.71% | A: 67.72%, G: 32.28% | 0.0763 |
| MXL | 64 | C: 85.16%, T: 14.84% | A: 74.22%, G: 25.78% | 0.0606 |
| PUR | 104 | C: 77.4%, T: 22.6% | A: 64.42%, G: 35.58% | 0.1425 |
| CLM | 94 | C: 78.72%, T: 21.28% | A: 68.62%, G: 31.38% | 0.1236 |
| PEL | 85 | C: 94.12%, T: 5.88% | A: 65.88%, G: 34.12% | 0.0005 |
| EAS | 504 | C: 99.21%, T: 0.79% | A: 82.34%, G: 17.66% | 0.0017 |
| CHB | 103 | C: 100.0%, T: 0.0% | A: 75.24%, G: 24.76% | NA |
| JPT | 104 | C: 100.0%, T: 0.0% | A: 79.33%, G: 20.67% | NA |
| CHS | 105 | C: 99.52%, T: 0.48% | A: 80.95%, G: 19.05% | 0.0011 |
| CDX | 93 | C: 98.92%, T: 1.08% | A: 86.02%, G: 13.98% | 0.0018 |
| KHV | 99 | C: 97.47%, T: 2.53% | A: 90.91%, G: 9.09% | 0.0026 |
| EUR | 503 | C: 75.35%, T: 24.65% | A: 57.06%, G: 42.94% | 0.2371 |
| CEU | 99 | C: 68.18%, T: 31.82% | A: 63.64%, G: 36.36% | 0.2667 |
| TSI | 107 | C: 76.17%, T: 23.83% | A: 50.93%, G: 49.07% | 0.2778 |
| FIN | 99 | C: 85.86%, T: 14.14% | A: 51.52%, G: 48.48% | 0.155 |
| GBR | 91 | C: 75.27%, T: 24.73% | A: 58.24%, G: 41.76% | 0.2355 |
| IBS | 107 | C: 71.5%, T: 28.5% | A: 61.21%, G: 38.79% | 0.2317 |
| SAS | 489 | C: 89.47%, T: 10.53% | A: 81.19%, G: 18.81% | 0.0273 |
| GIH | 103 | C: 92.23%, T: 7.77% | A: 81.07%, G: 18.93% | 0.0197 |
| PJL | 96 | C: 86.46%, T: 13.54% | A: 79.17%, G: 20.83% | 0.0412 |
| BEB | 86 | C: 91.28%, T: 8.72% | A: 83.14%, G: 16.86% | 0.0194 |
| STU | 102 | C: 88.73%, T: 11.27% | A: 81.86%, G: 18.14% | 0.0282 |
| ITU | 102 | C: 88.73%, T: 11.27% | A: 80.88%, G: 19.12% | 0.03 |

Abbreviations: ACB, African Caribbeans in Barbados; AFR, All African; ALL, All populations; AMR, Ad Mixed American; ASW, Americans of African Ancestry in SW USA; BEB, Bengali from Bangladesh; CDX, Chinese Dai in Xishuangbanna, China; CEU, Utah Residents (CEPH) with Northern and Western European Ancestry; CHB, Han Chinese in Beijing, China; CHS, Southern Han Chinese; CLM, Colombians from Medellin, Colombia; EAS, All East Asian; ESN, Esan in Nigeria; EUR, European; FIN, Finnish in Finland; GBR, British in England and Scotland; GIH, Gujarati Indian from Houston, Texas; GWD, Gambian in Western Divisions in the Gambia; IBS, Iberian Population in Spain; ITU, Indian Telugu from the UK; JPT, Japanese in Tokyo, Japan; KHV, Kinh in Ho Chi Minh City, Vietnam; LWK, Luhya in Webuye, Kenya; MSL, Mende in Sierra Leone; MXL, Mexican Ancestry from Los Angeles USA; PEL, Peruvians from Lima, Peru; PJL, Punjabi from Lahore, Pakistan; PUR, Puerto Ricans from Puerto Rico; SAS, South Asian; STU, Sri Lankan Tamil from the UK; TSI, Toscani in Italia; YRI, Yoruba in Ibadan, Nigeria.

**S22 Table.** Pairwise linkage disequilibrium (R^2^) for rs2066842 and rs2111234 NOD2 SNPs.

| **Population** | **N** | **rs2066842 Allele Freq** | **rs2111234 Allele Freq** | **R^2^** |
| --- | --- | --- | --- | --- |
| ALL | 2504 | C: 89.8%, T: 10.2% | G: 48.7%, A: 51.3% | 0.107 |
| AFR | 661 | C: 97.28%, T: 2.72% | G: 33.96%, A: 66.04% | 0.0144 |
| YRI | 108 | C: 99.54%, T: 0.46% | G: 35.65%, A: 64.35% | 0.0026 |
| LWK | 99 | C: 98.48%, T: 1.52% | G: 37.37%, A: 62.63% | 0.0092 |
| GWD | 113 | C: 95.13%, T: 4.87% | G: 29.65%, A: 70.35% | 0.0216 |
| MSL | 85 | C: 99.41%, T: 0.59% | G: 36.47%, A: 63.53% | 0.0034 |
| ESN | 99 | C: 99.49%, T: 0.51% | G: 31.82%, A: 68.18% | 0.0024 |
| ASW | 61 | C: 88.52%, T: 11.48% | G: 34.43%, A: 65.57% | 0.0681 |
| ACB | 96 | C: 97.4%, T: 2.6% | G: 33.33%, A: 66.67% | 0.0134 |
| AMR | 347 | C: 83.29%, T: 16.71% | G: 48.41%, A: 51.59% | 0.1817 |
| MXL | 64 | C: 85.16%, T: 14.84% | G: 58.59%, A: 41.41% | 0.2467 |
| PUR | 104 | C: 77.4%, T: 22.6% | G: 36.54%, A: 63.46% | 0.1491 |
| CLM | 94 | C: 78.72%, T: 21.28% | G: 42.02%, A: 57.98% | 0.1959 |
| PEL | 85 | C: 94.12%, T: 5.88% | G: 62.35%, A: 37.65% | 0.1035 |
| EAS | 504 | C: 99.21%, T: 0.79% | G: 76.49%, A: 23.51% | 0.026 |
| CHB | 103 | C: 100.0%, T: 0.0% | G: 72.33%, A: 27.67% | NA |
| JPT | 104 | C: 100.0%, T: 0.0% | G: 73.08%, A: 26.92% | NA |
| CHS | 105 | C: 99.52%, T: 0.48% | G: 78.57%, A: 21.43% | 0.0175 |
| CDX | 93 | C: 98.92%, T: 1.08% | G: 76.88%, A: 23.12% | 0.0361 |
| KHV | 99 | C: 97.47%, T: 2.53% | G: 81.82%, A: 18.18% | 0.1166 |
| EUR | 503 | C: 75.35%, T: 24.65% | G: 29.22%, A: 70.78% | 0.1351 |
| CEU | 99 | C: 68.18%, T: 31.82% | G: 28.79%, A: 71.21% | 0.1887 |
| TSI | 107 | C: 76.17%, T: 23.83% | G: 26.17%, A: 73.83% | 0.1109 |
| FIN | 99 | C: 85.86%, T: 14.14% | G: 31.31%, A: 68.69% | 0.0751 |
| GBR | 91 | C: 75.27%, T: 24.73% | G: 29.12%, A: 70.88% | 0.135 |
| IBS | 107 | C: 71.5%, T: 28.5% | G: 30.84%, A: 69.16% | 0.1778 |
| SAS | 489 | C: 89.47%, T: 10.53% | G: 60.22%, A: 39.78% | 0.1782 |
| GIH | 103 | C: 92.23%, T: 7.77% | G: 60.68%, A: 39.32% | 0.13 |
| PJL | 96 | C: 86.46%, T: 13.54% | G: 55.73%, A: 44.27% | 0.1972 |
| BEB | 86 | C: 91.28%, T: 8.72% | G: 62.79%, A: 37.21% | 0.1612 |
| STU | 102 | C: 88.73%, T: 11.27% | G: 60.78%, A: 39.22% | 0.197 |
| ITU | 102 | C: 88.73%, T: 11.27% | G: 61.27%, A: 38.73% | 0.2011 |

Abbreviations: ACB, African Caribbeans in Barbados; AFR, All African; ALL, All populations; AMR, Ad Mixed American; ASW, Americans of African Ancestry in SW USA; BEB, Bengali from Bangladesh; CDX, Chinese Dai in Xishuangbanna, China; CEU, Utah Residents (CEPH) with Northern and Western European Ancestry; CHB, Han Chinese in Beijing, China; CHS, Southern Han Chinese; CLM, Colombians from Medellin, Colombia; EAS, All East Asian; ESN, Esan in Nigeria; EUR, European; FIN, Finnish in Finland; GBR, British in England and Scotland; GIH, Gujarati Indian from Houston, Texas; GWD, Gambian in Western Divisions in the Gambia; IBS, Iberian Population in Spain; ITU, Indian Telugu from the UK; JPT, Japanese in Tokyo, Japan; KHV, Kinh in Ho Chi Minh City, Vietnam; LWK, Luhya in Webuye, Kenya; MSL, Mende in Sierra Leone; MXL, Mexican Ancestry from Los Angeles USA; PEL, Peruvians from Lima, Peru; PJL, Punjabi from Lahore, Pakistan; PUR, Puerto Ricans from Puerto Rico; SAS, South Asian; STU, Sri Lankan Tamil from the UK; TSI, Toscani in Italia; YRI, Yoruba in Ibadan, Nigeria.

**S23 Table.** Pairwise linkage disequilibrium (R^2^) for rs2066844 and rs5743278 NOD2 SNPs.

| **Population** | **N** | **rs2066844 Allele Freq** | **rs5743278 Allele Freq** | **R^2^** |
| --- | --- | --- | --- | --- |
| ALL | 2504 | C: 98.56%, T: 1.44% | C: 98.16%, G: 1.84% | 0.0003 |
| AFR | 661 | C: 99.77%, T: 0.23% | C: 93.27%, G: 6.73% | 0.0002 |
| YRI | 108 | C: 100.0%, T: 0.0% | C: 93.06%, G: 6.94% | NA |
| LWK | 99 | C: 100.0%, T: 0.0% | C: 88.89%, G: 11.11% | NA |
| GWD | 113 | C: 100.0%, T: 0.0% | C: 95.58%, G: 4.42% | NA |
| MSL | 85 | C: 100.0%, T: 0.0% | C: 93.53%, G: 6.47% | NA |
| ESN | 99 | C: 100.0%, T: 0.0% | C: 93.43%, G: 6.57% | NA |
| ASW | 61 | C: 98.36%, T: 1.64% | C: 93.44%, G: 6.56% | 0.0012 |
| ACB | 96 | C: 99.48%, T: 0.52% | C: 94.79%, G: 5.21% | 0.0003 |
| AMR | 347 | C: 97.55%, T: 2.45% | C: 99.57%, G: 0.43% | 0.0001 |
| MXL | 64 | C: 100.0%, T: 0.0% | C: 100.0%, G: 0.0% | NA |
| PUR | 104 | C: 97.12%, T: 2.88% | C: 99.52%, G: 0.48% | 0.0001 |
| CLM | 94 | C: 94.68%, T: 5.32% | C: 99.47%, G: 0.53% | 0.0003 |
| PEL | 85 | C: 99.41%, T: 0.59% | C: 99.41%, G: 0.59% | 0 |
| EAS | 504 | C: 100.0%, T: 0.0% | C: 100.0%, G: 0.0% | NA |
| CHB | 103 | C: 100.0%, T: 0.0% | C: 100.0%, G: 0.0% | NA |
| JPT | 104 | C: 100.0%, T: 0.0% | C: 100.0%, G: 0.0% | NA |
| CHS | 105 | C: 100.0%, T: 0.0% | C: 100.0%, G: 0.0% | NA |
| CDX | 93 | C: 100.0%, T: 0.0% | C: 100.0%, G: 0.0% | NA |
| KHV | 99 | C: 100.0%, T: 0.0% | C: 100.0%, G: 0.0% | NA |
| EUR | 503 | C: 94.93%, T: 5.07% | C: 100.0%, G: 0.0% | NA |
| CEU | 99 | C: 92.93%, T: 7.07% | C: 100.0%, G: 0.0% | NA |
| TSI | 107 | C: 95.33%, T: 4.67% | C: 100.0%, G: 0.0% | NA |
| FIN | 99 | C: 96.97%, T: 3.03% | C: 100.0%, G: 0.0% | NA |
| GBR | 91 | C: 95.05%, T: 4.95% | C: 100.0%, G: 0.0% | NA |
| IBS | 107 | C: 94.39%, T: 5.61% | C: 100.0%, G: 0.0% | NA |
| SAS | 489 | C: 99.9%, T: 0.1% | C: 100.0%, G: 0.0% | NA |
| GIH | 103 | C: 100.0%, T: 0.0% | C: 100.0%, G: 0.0% | NA |
| PJL | 96 | C: 100.0%, T: 0.0% | C: 100.0%, G: 0.0% | NA |
| BEB | 86 | C: 99.42%, T: 0.58% | C: 100.0%, G: 0.0% | NA |
| STU | 102 | C: 100.0%, T: 0.0% | C: 100.0%, G: 0.0% | NA |
| ITU | 102 | C: 100.0%, T: 0.0% | C: 100.0%, G: 0.0% | NA |

Abbreviations: ACB, African Caribbeans in Barbados; AFR, All African; ALL, All populations; AMR, Ad Mixed American; ASW, Americans of African Ancestry in SW USA; BEB, Bengali from Bangladesh; CDX, Chinese Dai in Xishuangbanna, China; CEU, Utah Residents (CEPH) with Northern and Western European Ancestry; CHB, Han Chinese in Beijing, China; CHS, Southern Han Chinese; CLM, Colombians from Medellin, Colombia; EAS, All East Asian; ESN, Esan in Nigeria; EUR, European; FIN, Finnish in Finland; GBR, British in England and Scotland; GIH, Gujarati Indian from Houston, Texas; GWD, Gambian in Western Divisions in the Gambia; IBS, Iberian Population in Spain; ITU, Indian Telugu from the UK; JPT, Japanese in Tokyo, Japan; KHV, Kinh in Ho Chi Minh City, Vietnam; LWK, Luhya in Webuye, Kenya; MSL, Mende in Sierra Leone; MXL, Mexican Ancestry from Los Angeles USA; PEL, Peruvians from Lima, Peru; PJL, Punjabi from Lahore, Pakistan; PUR, Puerto Ricans from Puerto Rico; SAS, South Asian; STU, Sri Lankan Tamil from the UK; TSI, Toscani in Italia; YRI, Yoruba in Ibadan, Nigeria.

**S24 Table.** Pairwise linkage disequilibrium (R^2^) for rs2066844 and rs6500328 NOD2 SNPs.

| **Population** | **N** | **rs2066844 Allele Freq** | **rs6500328 Allele Freq** | **R^2^** |
| --- | --- | --- | --- | --- |
| ALL | 2504 | C: 98.56%, T: 1.44% | A: 67.25%, G: 32.75% | 0.0065 |
| AFR | 661 | C: 99.77%, T: 0.23% | A: 52.95%, G: 47.05% | 0.002 |
| YRI | 108 | C: 100.0%, T: 0.0% | A: 53.24%, G: 46.76% | NA |
| LWK | 99 | C: 100.0%, T: 0.0% | A: 46.46%, G: 53.54% | NA |
| GWD | 113 | C: 100.0%, T: 0.0% | A: 57.52%, G: 42.48% | NA |
| MSL | 85 | C: 100.0%, T: 0.0% | A: 53.53%, G: 46.47% | NA |
| ESN | 99 | C: 100.0%, T: 0.0% | A: 50.51%, G: 49.49% | NA |
| ASW | 61 | C: 98.36%, T: 1.64% | A: 60.66%, G: 39.34% | 0.0108 |
| ACB | 96 | C: 99.48%, T: 0.52% | A: 51.04%, G: 48.96% | 0.005 |
| AMR | 347 | C: 97.55%, T: 2.45% | A: 67.72%, G: 32.28% | 0.012 |
| MXL | 64 | C: 100.0%, T: 0.0% | A: 74.22%, G: 25.78% | NA |
| PUR | 104 | C: 97.12%, T: 2.88% | A: 64.42%, G: 35.58% | 0.0164 |
| CLM | 94 | C: 94.68%, T: 5.32% | A: 68.62%, G: 31.38% | 0.0257 |
| PEL | 85 | C: 99.41%, T: 0.59% | A: 65.88%, G: 34.12% | 0.0031 |
| EAS | 504 | C: 100.0%, T: 0.0% | A: 82.34%, G: 17.66% | NA |
| CHB | 103 | C: 100.0%, T: 0.0% | A: 75.24%, G: 24.76% | NA |
| JPT | 104 | C: 100.0%, T: 0.0% | A: 79.33%, G: 20.67% | NA |
| CHS | 105 | C: 100.0%, T: 0.0% | A: 80.95%, G: 19.05% | NA |
| CDX | 93 | C: 100.0%, T: 0.0% | A: 86.02%, G: 13.98% | NA |
| KHV | 99 | C: 100.0%, T: 0.0% | A: 90.91%, G: 9.09% | NA |
| EUR | 503 | C: 94.93%, T: 5.07% | A: 57.06%, G: 42.94% | 0.0366 |
| CEU | 99 | C: 92.93%, T: 7.07% | A: 63.64%, G: 36.36% | 0.0435 |
| TSI | 107 | C: 95.33%, T: 4.67% | A: 50.93%, G: 49.07% | 0.0472 |
| FIN | 99 | C: 96.97%, T: 3.03% | A: 51.52%, G: 48.48% | 0.0294 |
| GBR | 91 | C: 95.05%, T: 4.95% | A: 58.24%, G: 41.76% | 0.0373 |
| IBS | 107 | C: 94.39%, T: 5.61% | A: 61.21%, G: 38.79% | 0.0232 |
| SAS | 489 | C: 99.9%, T: 0.1% | A: 81.19%, G: 18.81% | 0.0002 |
| GIH | 103 | C: 100.0%, T: 0.0% | A: 81.07%, G: 18.93% | NA |
| PJL | 96 | C: 100.0%, T: 0.0% | A: 79.17%, G: 20.83% | NA |
| BEB | 86 | C: 99.42%, T: 0.58% | A: 83.14%, G: 16.86% | 0.0012 |
| STU | 102 | C: 100.0%, T: 0.0% | A: 81.86%, G: 18.14% | NA |
| ITU | 102 | C: 100.0%, T: 0.0% | A: 80.88%, G: 19.12% | NA |

Abbreviations: ACB, African Caribbeans in Barbados; AFR, All African; ALL, All populations; AMR, Ad Mixed American; ASW, Americans of African Ancestry in SW USA; BEB, Bengali from Bangladesh; CDX, Chinese Dai in Xishuangbanna, China; CEU, Utah Residents (CEPH) with Northern and Western European Ancestry; CHB, Han Chinese in Beijing, China; CHS, Southern Han Chinese; CLM, Colombians from Medellin, Colombia; EAS, All East Asian; ESN, Esan in Nigeria; EUR, European; FIN, Finnish in Finland; GBR, British in England and Scotland; GIH, Gujarati Indian from Houston, Texas; GWD, Gambian in Western Divisions in the Gambia; IBS, Iberian Population in Spain; ITU, Indian Telugu from the UK; JPT, Japanese in Tokyo, Japan; KHV, Kinh in Ho Chi Minh City, Vietnam; LWK, Luhya in Webuye, Kenya; MSL, Mende in Sierra Leone; MXL, Mexican Ancestry from Los Angeles USA; PEL, Peruvians from Lima, Peru; PJL, Punjabi from Lahore, Pakistan; PUR, Puerto Ricans from Puerto Rico; SAS, South Asian; STU, Sri Lankan Tamil from the UK; TSI, Toscani in Italia; YRI, Yoruba in Ibadan, Nigeria.

**S25 Table.** Pairwise linkage disequilibrium (R^2^) for rs2066844 and rs2111234 NOD2 SNPs.

| **Population** | **N** | **rs2066844 Allele Freq** | **rs2111234 Allele Freq** | **R^2^** |
| --- | --- | --- | --- | --- |
| ALL | 2504 | C: 98.56%, T: 1.44% | G: 48.7%, A: 51.3% | 0.0138 |
| AFR | 661 | C: 99.77%, T: 0.23% | G: 33.96%, A: 66.04% | 0.0012 |
| YRI | 108 | C: 100.0%, T: 0.0% | G: 35.65%, A: 64.35% | NA |
| LWK | 99 | C: 100.0%, T: 0.0% | G: 37.37%, A: 62.63% | NA |
| GWD | 113 | C: 100.0%, T: 0.0% | G: 29.65%, A: 70.35% | NA |
| MSL | 85 | C: 100.0%, T: 0.0% | G: 36.47%, A: 63.53% | NA |
| ESN | 99 | C: 100.0%, T: 0.0% | G: 31.82%, A: 68.18% | NA |
| ASW | 61 | C: 98.36%, T: 1.64% | G: 34.43%, A: 65.57% | 0.0088 |
| ACB | 96 | C: 99.48%, T: 0.52% | G: 33.33%, A: 66.67% | 0.0026 |
| AMR | 347 | C: 97.55%, T: 2.45% | G: 48.41%, A: 51.59% | 0.0236 |
| MXL | 64 | C: 100.0%, T: 0.0% | G: 58.59%, A: 41.41% | NA |
| PUR | 104 | C: 97.12%, T: 2.88% | G: 36.54%, A: 63.46% | 0.0171 |
| CLM | 94 | C: 94.68%, T: 5.32% | G: 42.02%, A: 57.98% | 0.0407 |
| PEL | 85 | C: 99.41%, T: 0.59% | G: 62.35%, A: 37.65% | 0.0098 |
| EAS | 504 | C: 100.0%, T: 0.0% | G: 76.49%, A: 23.51% | NA |
| CHB | 103 | C: 100.0%, T: 0.0% | G: 72.33%, A: 27.67% | NA |
| JPT | 104 | C: 100.0%, T: 0.0% | G: 73.08%, A: 26.92% | NA |
| CHS | 105 | C: 100.0%, T: 0.0% | G: 78.57%, A: 21.43% | NA |
| CDX | 93 | C: 100.0%, T: 0.0% | G: 76.88%, A: 23.12% | NA |
| KHV | 99 | C: 100.0%, T: 0.0% | G: 81.82%, A: 18.18% | NA |
| EUR | 503 | C: 94.93%, T: 5.07% | G: 29.22%, A: 70.78% | 0.0221 |
| CEU | 99 | C: 92.93%, T: 7.07% | G: 28.79%, A: 71.21% | 0.0308 |
| TSI | 107 | C: 95.33%, T: 4.67% | G: 26.17%, A: 73.83% | 0.0174 |
| FIN | 99 | C: 96.97%, T: 3.03% | G: 31.31%, A: 68.69% | 0.0142 |
| GBR | 91 | C: 95.05%, T: 4.95% | G: 29.12%, A: 70.88% | 0.0214 |
| IBS | 107 | C: 94.39%, T: 5.61% | G: 30.84%, A: 69.16% | 0.0265 |
| SAS | 489 | C: 99.9%, T: 0.1% | G: 60.22%, A: 39.78% | 0.0015 |
| GIH | 103 | C: 100.0%, T: 0.0% | G: 60.68%, A: 39.32% | NA |
| PJL | 96 | C: 100.0%, T: 0.0% | G: 55.73%, A: 44.27% | NA |
| BEB | 86 | C: 99.42%, T: 0.58% | G: 62.79%, A: 37.21% | 0.0099 |
| STU | 102 | C: 100.0%, T: 0.0% | G: 60.78%, A: 39.22% | NA |
| ITU | 102 | C: 100.0%, T: 0.0% | G: 61.27%, A: 38.73% | NA |

Abbreviations: ACB, African Caribbeans in Barbados; AFR, All African; ALL, All populations; AMR, Ad Mixed American; ASW, Americans of African Ancestry in SW USA; BEB, Bengali from Bangladesh; CDX, Chinese Dai in Xishuangbanna, China; CEU, Utah Residents (CEPH) with Northern and Western European Ancestry; CHB, Han Chinese in Beijing, China; CHS, Southern Han Chinese; CLM, Colombians from Medellin, Colombia; EAS, All East Asian; ESN, Esan in Nigeria; EUR, European; FIN, Finnish in Finland; GBR, British in England and Scotland; GIH, Gujarati Indian from Houston, Texas; GWD, Gambian in Western Divisions in the Gambia; IBS, Iberian Population in Spain; ITU, Indian Telugu from the UK; JPT, Japanese in Tokyo, Japan; KHV, Kinh in Ho Chi Minh City, Vietnam; LWK, Luhya in Webuye, Kenya; MSL, Mende in Sierra Leone; MXL, Mexican Ancestry from Los Angeles USA; PEL, Peruvians from Lima, Peru; PJL, Punjabi from Lahore, Pakistan; PUR, Puerto Ricans from Puerto Rico; SAS, South Asian; STU, Sri Lankan Tamil from the UK; TSI, Toscani in Italia; YRI, Yoruba in Ibadan, Nigeria.

**S26 Table.** Pairwise linkage disequilibrium (R^2^) for rs5743278 and rs6500328 NOD2 SNPs.

| **Population** | **N** | **rs5743278 Allele Freq** | **rs6500328 Allele Freq** | **R^2^** |
| --- | --- | --- | --- | --- |
| ALL | 2504 | C: 98.16%, G: 1.84% | A: 67.25%, G: 32.75% | 0.036 |
| AFR | 661 | C: 93.27%, G: 6.73% | A: 52.95%, G: 47.05% | 0.0745 |
| YRI | 108 | C: 93.06%, G: 6.94% | A: 53.24%, G: 46.76% | 0.085 |
| LWK | 99 | C: 88.89%, G: 11.11% | A: 46.46%, G: 53.54% | 0.1085 |
| GWD | 113 | C: 95.58%, G: 4.42% | A: 57.52%, G: 42.48% | 0.0627 |
| MSL | 85 | C: 93.53%, G: 6.47% | A: 53.53%, G: 46.47% | 0.0797 |
| ESN | 99 | C: 93.43%, G: 6.57% | A: 50.51%, G: 49.49% | 0.0515 |
| ASW | 61 | C: 93.44%, G: 6.56% | A: 60.66%, G: 39.34% | 0.1082 |
| ACB | 96 | C: 94.79%, G: 5.21% | A: 51.04%, G: 48.96% | 0.037 |
| AMR | 347 | C: 99.57%, G: 0.43% | A: 67.72%, G: 32.28% | 0.0091 |
| MXL | 64 | C: 100.0%, G: 0.0% | A: 74.22%, G: 25.78% | NA |
| PUR | 104 | C: 99.52%, G: 0.48% | A: 64.42%, G: 35.58% | 0.0087 |
| CLM | 94 | C: 99.47%, G: 0.53% | A: 68.62%, G: 31.38% | 0.0117 |
| PEL | 85 | C: 99.41%, G: 0.59% | A: 65.88%, G: 34.12% | 0.0114 |
| EAS | 504 | C: 100.0%, G: 0.0% | A: 82.34%, G: 17.66% | NA |
| CHB | 103 | C: 100.0%, G: 0.0% | A: 75.24%, G: 24.76% | NA |
| JPT | 104 | C: 100.0%, G: 0.0% | A: 79.33%, G: 20.67% | NA |
| CHS | 105 | C: 100.0%, G: 0.0% | A: 80.95%, G: 19.05% | NA |
| CDX | 93 | C: 100.0%, G: 0.0% | A: 86.02%, G: 13.98% | NA |
| KHV | 99 | C: 100.0%, G: 0.0% | A: 90.91%, G: 9.09% | NA |
| EUR | 503 | C: 100.0%, G: 0.0% | A: 57.06%, G: 42.94% | NA |
| CEU | 99 | C: 100.0%, G: 0.0% | A: 63.64%, G: 36.36% | NA |
| TSI | 107 | C: 100.0%, G: 0.0% | A: 50.93%, G: 49.07% | NA |
| FIN | 99 | C: 100.0%, G: 0.0% | A: 51.52%, G: 48.48% | NA |
| GBR | 91 | C: 100.0%, G: 0.0% | A: 58.24%, G: 41.76% | NA |
| IBS | 107 | C: 100.0%, G: 0.0% | A: 61.21%, G: 38.79% | NA |
| SAS | 489 | C: 100.0%, G: 0.0% | A: 81.19%, G: 18.81% | NA |
| GIH | 103 | C: 100.0%, G: 0.0% | A: 81.07%, G: 18.93% | NA |
| PJL | 96 | C: 100.0%, G: 0.0% | A: 79.17%, G: 20.83% | NA |
| BEB | 86 | C: 100.0%, G: 0.0% | A: 83.14%, G: 16.86% | NA |
| STU | 102 | C: 100.0%, G: 0.0% | A: 81.86%, G: 18.14% | NA |
| ITU | 102 | C: 100.0%, G: 0.0% | A: 80.88%, G: 19.12% | NA |

Abbreviations: ACB, African Caribbeans in Barbados; AFR, All African; ALL, All populations; AMR, Ad Mixed American; ASW, Americans of African Ancestry in SW USA; BEB, Bengali from Bangladesh; CDX, Chinese Dai in Xishuangbanna, China; CEU, Utah Residents (CEPH) with Northern and Western European Ancestry; CHB, Han Chinese in Beijing, China; CHS, Southern Han Chinese; CLM, Colombians from Medellin, Colombia; EAS, All East Asian; ESN, Esan in Nigeria; EUR, European; FIN, Finnish in Finland; GBR, British in England and Scotland; GIH, Gujarati Indian from Houston, Texas; GWD, Gambian in Western Divisions in the Gambia; IBS, Iberian Population in Spain; ITU, Indian Telugu from the UK; JPT, Japanese in Tokyo, Japan; KHV, Kinh in Ho Chi Minh City, Vietnam; LWK, Luhya in Webuye, Kenya; MSL, Mende in Sierra Leone; MXL, Mexican Ancestry from Los Angeles USA; PEL, Peruvians from Lima, Peru; PJL, Punjabi from Lahore, Pakistan; PUR, Puerto Ricans from Puerto Rico; SAS, South Asian; STU, Sri Lankan Tamil from the UK; TSI, Toscani in Italia; YRI, Yoruba in Ibadan, Nigeria.

**S27 Table.** Pairwise linkage disequilibrium (R^2^) for rs5743278 and rs2111234 NOD2 SNPs.

| **Population** | **N** | **rs5743278 Allele Freq** | **rs2111234 Allele Freq** | **R^2^** |
| --- | --- | --- | --- | --- |
| ALL | 2504 | C: 98.16%, G: 1.84% | G: 48.7%, A: 51.3% | 0.0101 |
| AFR | 661 | C: 93.27%, G: 6.73% | G: 33.96%, A: 66.04% | 0.015 |
| YRI | 108 | C: 93.06%, G: 6.94% | G: 35.65%, A: 64.35% | 0.0413 |
| LWK | 99 | C: 88.89%, G: 11.11% | G: 37.37%, A: 62.63% | 0.0197 |
| GWD | 113 | C: 95.58%, G: 4.42% | G: 29.65%, A: 70.35% | 0.0086 |
| MSL | 85 | C: 93.53%, G: 6.47% | G: 36.47%, A: 63.53% | 0 |
| ESN | 99 | C: 93.43%, G: 6.57% | G: 31.82%, A: 68.18% | 0.0189 |
| ASW | 61 | C: 93.44%, G: 6.56% | G: 34.43%, A: 65.57% | 0.0368 |
| ACB | 96 | C: 94.79%, G: 5.21% | G: 33.33%, A: 66.67% | 0.0135 |
| AMR | 347 | C: 99.57%, G: 0.43% | G: 48.41%, A: 51.59% | 0.0041 |
| MXL | 64 | C: 100.0%, G: 0.0% | G: 58.59%, A: 41.41% | NA |
| PUR | 104 | C: 99.52%, G: 0.48% | G: 36.54%, A: 63.46% | 0.0028 |
| CLM | 94 | C: 99.47%, G: 0.53% | G: 42.02%, A: 57.98% | 0.0039 |
| PEL | 85 | C: 99.41%, G: 0.59% | G: 62.35%, A: 37.65% | 0.0098 |
| EAS | 504 | C: 100.0%, G: 0.0% | G: 76.49%, A: 23.51% | NA |
| CHB | 103 | C: 100.0%, G: 0.0% | G: 72.33%, A: 27.67% | NA |
| JPT | 104 | C: 100.0%, G: 0.0% | G: 73.08%, A: 26.92% | NA |
| CHS | 105 | C: 100.0%, G: 0.0% | G: 78.57%, A: 21.43% | NA |
| CDX | 93 | C: 100.0%, G: 0.0% | G: 76.88%, A: 23.12% | NA |
| KHV | 99 | C: 100.0%, G: 0.0% | G: 81.82%, A: 18.18% | NA |
| EUR | 503 | C: 100.0%, G: 0.0% | G: 29.22%, A: 70.78% | NA |
| CEU | 99 | C: 100.0%, G: 0.0% | G: 28.79%, A: 71.21% | NA |
| TSI | 107 | C: 100.0%, G: 0.0% | G: 26.17%, A: 73.83% | NA |
| FIN | 99 | C: 100.0%, G: 0.0% | G: 31.31%, A: 68.69% | NA |
| GBR | 91 | C: 100.0%, G: 0.0% | G: 29.12%, A: 70.88% | NA |
| IBS | 107 | C: 100.0%, G: 0.0% | G: 30.84%, A: 69.16% | NA |
| SAS | 489 | C: 100.0%, G: 0.0% | G: 60.22%, A: 39.78% | NA |
| GIH | 103 | C: 100.0%, G: 0.0% | G: 60.68%, A: 39.32% | NA |
| PJL | 96 | C: 100.0%, G: 0.0% | G: 55.73%, A: 44.27% | NA |
| BEB | 86 | C: 100.0%, G: 0.0% | G: 62.79%, A: 37.21% | NA |
| STU | 102 | C: 100.0%, G: 0.0% | G: 60.78%, A: 39.22% | NA |
| ITU | 102 | C: 100.0%, G: 0.0% | G: 61.27%, A: 38.73% | NA |

Abbreviations: ACB, African Caribbeans in Barbados; AFR, All African; ALL, All populations; AMR, Ad Mixed American; ASW, Americans of African Ancestry in SW USA; BEB, Bengali from Bangladesh; CDX, Chinese Dai in Xishuangbanna, China; CEU, Utah Residents (CEPH) with Northern and Western European Ancestry; CHB, Han Chinese in Beijing, China; CHS, Southern Han Chinese; CLM, Colombians from Medellin, Colombia; EAS, All East Asian; ESN, Esan in Nigeria; EUR, European; FIN, Finnish in Finland; GBR, British in England and Scotland; GIH, Gujarati Indian from Houston, Texas; GWD, Gambian in Western Divisions in the Gambia; IBS, Iberian Population in Spain; ITU, Indian Telugu from the UK; JPT, Japanese in Tokyo, Japan; KHV, Kinh in Ho Chi Minh City, Vietnam; LWK, Luhya in Webuye, Kenya; MSL, Mende in Sierra Leone; MXL, Mexican Ancestry from Los Angeles USA; PEL, Peruvians from Lima, Peru; PJL, Punjabi from Lahore, Pakistan; PUR, Puerto Ricans from Puerto Rico; SAS, South Asian; STU, Sri Lankan Tamil from the UK; TSI, Toscani in Italia; YRI, Yoruba in Ibadan, Nigeria.

**S28 Table.** Pairwise linkage disequilibrium (R^2^) for rs6500328 and rs2111234 NOD2 SNPs.

| **Population** | **N** | **rs6500328 Allele Freq** | **rs2111234 Allele Freq** | **R^2^** |
| --- | --- | --- | --- | --- |
| ALL | 2504 | A: 67.25%, G: 32.75% | G: 48.7%, A: 51.3% | 0.4139 |
| AFR | 661 | A: 52.95%, G: 47.05% | G: 33.96%, A: 66.04% | 0.3984 |
| YRI | 108 | A: 53.24%, G: 46.76% | G: 35.65%, A: 64.35% | 0.4599 |
| LWK | 99 | A: 46.46%, G: 53.54% | G: 37.37%, A: 62.63% | 0.4951 |
| GWD | 113 | A: 57.52%, G: 42.48% | G: 29.65%, A: 70.35% | 0.249 |
| MSL | 85 | A: 53.53%, G: 46.47% | G: 36.47%, A: 63.53% | 0.3696 |
| ESN | 99 | A: 50.51%, G: 49.49% | G: 31.82%, A: 68.18% | 0.4573 |
| ASW | 61 | A: 60.66%, G: 39.34% | G: 34.43%, A: 65.57% | 0.3405 |
| ACB | 96 | A: 51.04%, G: 48.96% | G: 33.33%, A: 66.67% | 0.4796 |
| AMR | 347 | A: 67.72%, G: 32.28% | G: 48.41%, A: 51.59% | 0.4391 |
| MXL | 64 | A: 74.22%, G: 25.78% | G: 58.59%, A: 41.41% | 0.4916 |
| PUR | 104 | A: 64.42%, G: 35.58% | G: 36.54%, A: 63.46% | 0.318 |
| CLM | 94 | A: 68.62%, G: 31.38% | G: 42.02%, A: 57.98% | 0.3315 |
| PEL | 85 | A: 65.88%, G: 34.12% | G: 62.35%, A: 37.65% | 0.8109 |
| EAS | 504 | A: 82.34%, G: 17.66% | G: 76.49%, A: 23.51% | 0.5616 |
| CHB | 103 | A: 75.24%, G: 24.76% | G: 72.33%, A: 27.67% | 0.6837 |
| JPT | 104 | A: 79.33%, G: 20.67% | G: 73.08%, A: 26.92% | 0.5387 |
| CHS | 105 | A: 80.95%, G: 19.05% | G: 78.57%, A: 21.43% | 0.6571 |
| CDX | 93 | A: 86.02%, G: 13.98% | G: 76.88%, A: 23.12% | 0.4877 |
| KHV | 99 | A: 90.91%, G: 9.09% | G: 81.82%, A: 18.18% | 0.391 |
| EUR | 503 | A: 57.06%, G: 42.94% | G: 29.22%, A: 70.78% | 0.2773 |
| CEU | 99 | A: 63.64%, G: 36.36% | G: 28.79%, A: 71.21% | 0.231 |
| TSI | 107 | A: 50.93%, G: 49.07% | G: 26.17%, A: 73.83% | 0.3414 |
| FIN | 99 | A: 51.52%, G: 48.48% | G: 31.31%, A: 68.69% | 0.2749 |
| GBR | 91 | A: 58.24%, G: 41.76% | G: 29.12%, A: 70.88% | 0.2946 |
| IBS | 107 | A: 61.21%, G: 38.79% | G: 30.84%, A: 69.16% | 0.2609 |
| SAS | 489 | A: 81.19%, G: 18.81% | G: 60.22%, A: 39.78% | 0.308 |
| GIH | 103 | A: 81.07%, G: 18.93% | G: 60.68%, A: 39.32% | 0.3604 |
| PJL | 96 | A: 79.17%, G: 20.83% | G: 55.73%, A: 44.27% | 0.3022 |
| BEB | 86 | A: 83.14%, G: 16.86% | G: 62.79%, A: 37.21% | 0.2712 |
| STU | 102 | A: 81.86%, G: 18.14% | G: 60.78%, A: 39.22% | 0.3136 |
| ITU | 102 | A: 80.88%, G: 19.12% | G: 61.27%, A: 38.73% | 0.286 |

Abbreviations: ACB, African Caribbeans in Barbados; AFR, All African; ALL, All populations; AMR, Ad Mixed American; ASW, Americans of African Ancestry in SW USA; BEB, Bengali from Bangladesh; CDX, Chinese Dai in Xishuangbanna, China; CEU, Utah Residents (CEPH) with Northern and Western European Ancestry; CHB, Han Chinese in Beijing, China; CHS, Southern Han Chinese; CLM, Colombians from Medellin, Colombia; EAS, All East Asian; ESN, Esan in Nigeria; EUR, European; FIN, Finnish in Finland; GBR, British in England and Scotland; GIH, Gujarati Indian from Houston, Texas; GWD, Gambian in Western Divisions in the Gambia; IBS, Iberian Population in Spain; ITU, Indian Telugu from the UK; JPT, Japanese in Tokyo, Japan; KHV, Kinh in Ho Chi Minh City, Vietnam; LWK, Luhya in Webuye, Kenya; MSL, Mende in Sierra Leone; MXL, Mexican Ancestry from Los Angeles USA; PEL, Peruvians from Lima, Peru; PJL, Punjabi from Lahore, Pakistan; PUR, Puerto Ricans from Puerto Rico; SAS, South Asian; STU, Sri Lankan Tamil from the UK; TSI, Toscani in Italia; YRI, Yoruba in Ibadan, Nigeria.
